# Linking Reductions in Alcohol and Birth Control Use Risk Behavior to Prevention of Alcohol‐Exposed Pregnancy: A Population‐Level Simulation of Preconceptual Prevention Programs

**DOI:** 10.1111/acer.70379

**Published:** 2026-07-22

**Authors:** Arielle R. Deutsch, Ali Akhavan, Mohammad S. Jalali

**Affiliations:** ^1^ Avera Research Institute, Avera Health Sioux Falls South Dakota USA; ^2^ Sanford School of Medicine, University of South Dakota Vermillion South Dakota USA; ^3^ Center for Health Technology Assessment Massachusetts General Hospital, Harvard Medical School Boston Massachusetts USA; ^4^ Sloan School of Management, Massachusetts Institute of Technology Cambridge Massachusetts USA

**Keywords:** alcohol prevention programing, alcohol‐exposed pregnancy, simulation study

## Abstract

**Background:**

The most popular alcohol‐exposed pregnancy (AEP) prevention program (AEP‐P) is a brief intervention that dually promotes reducing alcohol use and increasing birth control use. The primary outcome variable used to evaluate AEP‐P involves aggregating behavioral change into a binary variable, despite differences in how AEP‐P impacts each behavior. This study examined if all three possible AEP‐P behavior‐change outcomes (alcohol only (AO), birth control only (BCO), or both behaviors [BB]) are equally effective in preventing subsequent AEP itself.

**Methods:**

We simulated implementing an AEP‐P in a United States small metropolitan area under 252 different scenarios (policy tests), varying the (1) percent of the eligible population who changed AEP risk behavior; (2) proportion of AEP‐P participants in AO, BCO, and BB groups; and (3) ratio of participants changing alcohol use to non‐risky drinking or abstinence. We calculated the percent difference in next‐year new cases of total AEP, high‐risk AEP, and low‐risk AEP for each policy test compared to a “no implementation” baseline.

**Results:**

Policies with higher BB proportions had larger reductions for total and high‐risk AEP, with the 100% BB policy providing the highest reductions (5.58%–17.13% and 16.27%– 42.43% total and high‐risk AEP, respectively). All policies increased low‐risk AEP, except for the 100% BCO policy. Policies with higher BCO proportions resulted in lower increases in low‐risk AEP, but lower reductions to high‐risk AEP. Policy increases in low‐risk AEP were driven by the fraction of people transitioning to abstention compared to non‐risky drinking.

**Conclusions:**

Study results suggested that different constructs of behavioral changes made during AEP‐P confer different levels of preventing AEP itself. Using a binary AEP risk aggregate variable may bias our understanding of AEP‐P preventative efficacy and inhibit further understanding of how to improve AEP‐P.

## Graphical abstract

A simulation model of an alcohol‐exposed pregnancy (AEP) preconceptual prevention program (AEP‐P) examined how low‐risk and high‐risk AEP prevention could be impacted by the percent of participants changing alcohol use only (AO), birth control use only (BCO), or both behaviors (BB). Results indicated that BB and BCO change were associated with the highest and lowest reductions of high‐risk AEP, respectively. The study highlights the limitations of using a binary AEP risk aggregate to examine AEP‐P programmatic efficacy for preventing AEP.

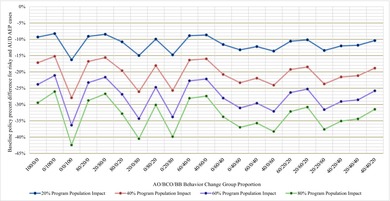

## 1 Introduction

Alcohol consumption during pregnancy (alcohol‐exposed pregnancy; AEP) is associated with a wide range of negative outcomes for birth and long‐term offspring health (Popova and Dozet 2023; Dejong et al. 2019). Approximately 14% and 5% of pregnant people in the United States report any drinking and binge drinking, respectively (Howard et al. 2022; Gosdin et al. 2022). AEP risk estimates for non‐pregnant women of childbearing age are approximately 1.2%–3.4% (Roberts and Thompson 2019; Green 2016). As AEP is most common in the first trimester (Alshaarawy et al. 2016) prior to pregnancy awareness (Hutchinson et al. 2022), preconceptual prevention (Reid et al. 2021) is especially important. The most popular preconceptual AEP prevention program (AEP‐P) involves a brief intervention that dually targets alcohol and birth control use risk behavior. The initial program, Project CHOICES (Floyd et al. 2007, Group* 2003), utilized the transtheoretical model framework to guide participants to choose the behavior(s) they are motivated to change (Group* 2003). Subsequent CHOICES‐based programs have examined various dosages (e.g., 1–6 sessions), modalities (Ceperich and Ingersoll 2011; Wilton et al. 2013), settings (Floyd et al. 2007; Velasquez et al. 2017), and populations (Hanson et al. 2017; Rendall‐Mkosi et al. 2013).

Across these variations, most AEP‐Ps share a strategy for measuring AEP risk, which determines participant eligibility and evaluates program efficacy. This measure is a binary variable that aggregates risk thresholds for alcohol (e.g., past 90‐day history of binge or heavy drinking; 4+ / 7+ standard drinks per session/week (National Institute on Alcohol Abuse and Alcoholism n.d.)) and birth control (e.g., past 90‐day history of penile‐vaginal intercourse with inconsistent, ineffective, or no use of birth control). Fertile women of childbearing age are eligible for AEP‐P participation if they meet both risk thresholds. Programmatic efficacy (reducing AEP risk) is defined as changing alcohol *and/or* birth control use behavior to a below‐risk threshold.

There is clear evidence that AEP‐Ps effectively reduce AEP risk behavior, especially if the aggregate AEP risk variable is used as the outcome (Farrell‐Carnahan et al. 2013; Letourneau et al. 2017). However, many studies report that this AEP risk reduction is driven by participants changing birth control compared to alcohol use risk behavior (Hanson et al. 2017; Ingersoll et al. 2018; Wilton et al. 2013; Tenkku et al. 2011; Rendall‐Mkosi et al. 2013). Differences in the percent of participants who change alcohol use risk behavior compared to birth control use range from less than 10% (e.g., 49% alcohol, 56% birth control (Floyd et al. 2007)) to more than 30% (34% alcohol, 69% birth control (Ceperich and Ingersoll 2011)). These differences are contingent upon the proportion of AEP‐P participants who change alcohol use only (AO), birth control use only (BCO), or both behaviors (BB) (e.g., “behavior‐change group”). The few studies that report behavior‐change groups indicate that both BB and BCO are popular options. The original CHOICES pilot study reported 47% BB, 18% AO, and 34% BCO at 6 months (Group* 2003), while the randomized clinical trial reported 47% BB, 33% AO, and 19% BCO at 9 months (Floyd et al. 2007). Other studies report that BCO is the largest group (e.g., 45%–60% (Hutton et al. 2014, Hanson et al. 2017)) or the second largest group after BB (e.g., 36% BCO, 40% BB (Velasquez et al. 2017)).

### 1.1 Deconstructing AEP Risk Effects on Preventing AEP Outcomes

Although AEP‐Ps can reduce AEP risk behavior, this evidence is largely based on the aggregate AEP risk variable. However, this variable includes an assumption that all risk reduction changes (AO, BCO, or BB) equally prevent AEP. Testing this assumption is important for evaluating AEP‐P preventative efficacy, given differences in risk behavior change. Although AEP risk behavior changes have shown to be stable over 6–12 months (Velasquez et al. 2017; Hanson et al. 2013), reduced risk of AEP for BCO participants may be less sustainable. As heavier drinking reduces consistent or effective birth control use (Cho and Yang 2023; Simons et al. 2018) and impairs sexual decision‐making (Garcia et al. 2019; Stevely et al. 2020), vulnerability to AEP risk for BCO participants may depend on which birth control they use. AEP‐P participants often select types of birth control that require more effortful control (e.g., condoms, hormonal birth control pills, or sexual abstinence (Hanson et al. 2017, Hutton et al. 2014)). Birth control failure rates (Trussell 2011) are also uniquely important for the BCO group.

The AEP‐P alcohol risk threshold aggregates low‐risk drinking (below threshold) and abstention together, given that CHOICES uses a controlled drinking approach (participants can choose either abstinence or low‐risk drinking). Controlled drinking programs are beneficial; low‐risk goals can be more easily achieved (Votaw and Witkiewitz 2023); and controlled drinking programs mitigate treatment resistance (Henssler et al. 2021). However, abstinence‐related goals/changes are related to lower episodes of risky drinking up to 3 years later (Witkiewitz et al. 2018). As AEP‐P participants are more likely to change their alcohol use behavior to below‐threshold drinking levels than abstention (Farrell‐Carnahan et al. 2013; Ceperich and Ingersoll 2011), risk reduction may be less stable for low‐risk drinking change. Although research on the relation between low‐risk drinking and negative pregnancy and offspring outcomes remains inconclusive, low‐risk drinking has been linked to negative birthing (Sundermann et al. 2019) and offspring (Easey et al. 2019) outcomes.

Few studies examine how AEP‐P impacts AEP directly, given limited follow‐up time frames and attrition (attrition ranging from 2% (Letourneau et al. 2017) to 49% (Hanson et al. 2017) for a 6‐month follow‐up). One strategy to overcome these challenges is to use computer‐based, mathematical simulation modeling approaches. When properly grounded in data, literature, and theory, simulation models provide a powerful way to examine population‐level program effects. One prior simulation model (Yaesoubi et al. 2022) demonstrated that increasing effective birth control use decreased AEP odds by 51%, abstaining from drinking decreased odds by 71%, and engaging in both behaviors decreased odds by 80%. However, this study did not specifically consider behavioral change and risk as operationalized by AEP‐P.

### 1.2 Current Study

The purpose of this study is to test an assumption that underlies both the development and evaluations of AEP‐Ps; specifically, we examine whether risk reduction via AO, BCO, or BB results in equal levels of AEP prevention. We use a system dynamics simulation (Sterman 2018) to examine how the number of annual AEP cases may vary as a function of behavior‐change group proportions for a single year AEP‐P “implementation” within a population. Simulations involved comparing differences in next‐year new AEP cases between a baseline simulation representing the “status quo” (no AEP‐P implementation) and 84 different implementation scenarios (policy tests). AEP‐P policy tests varied in (1) the overall percent of participants who change AEP risk behavior, (2) behavior‐change group proportions, and (3) the ratio of people who changed alcohol use to either non‐risky drinking or abstinence. The current model was built on a previously developed simulation model (Deutsch, Motobar, et al. 2022), as part of a larger community‐based system dynamics project (Hovmand 2014) focusing on alcohol and substance use during pregnancy within a Northern Plains United States small metropolitan area (Deutsch, Lustfield, and Jalali 2022).

## 2 Methods

### 2.1 Systems Modeling

System dynamics is a simulation‐based computational method that can be used to examine complex public health issues such as alcohol use behavior and related consequences (Naumann et al. 2022). System dynamics models examine macro‐level nonlinear dynamic change in system behaviors through key feedback loops and variable interactions (Sterman 2018). Through this approach, our model does not focus on individual (participant) level effects and outcomes, but rather community‐level change based on the proportion of community members who enroll and successfully change their behavior. Quantitative models involve a series of ordinary differential equations that represent dynamic change over time through stock and flow model structures. Stocks represent accumulation or depletion of a quantitative entity over time (e.g., population of people in a city). Inflows and outflows drive change in stocks (e.g., people moving into and out of a city per year). Multiple stocks that represent different states of an entity can connect to each other to represent transitions (e.g., separate stocks for single and married people connected by separate “getting married” and “getting divorced” flows).

### 2.2 Model Development

**FIGURE 1:**
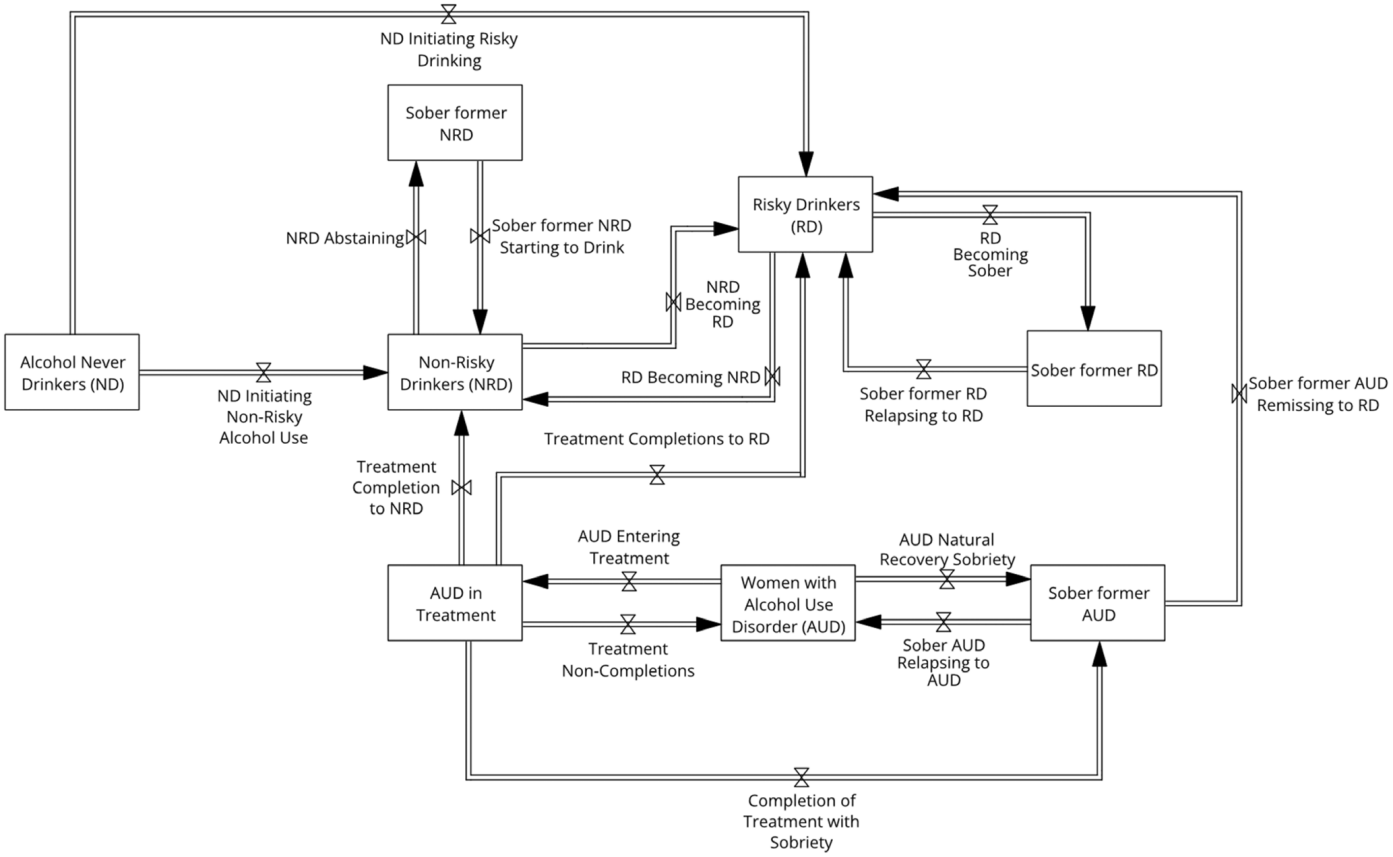
Alcohol‐specific model structures, representing stages (stocks) and transitions (flows). Population dynamic flows (e.g., aging in/out, death, and population mobility) not shown. Full model with all variables available in the online supporting information.

**FIGURE 2:**
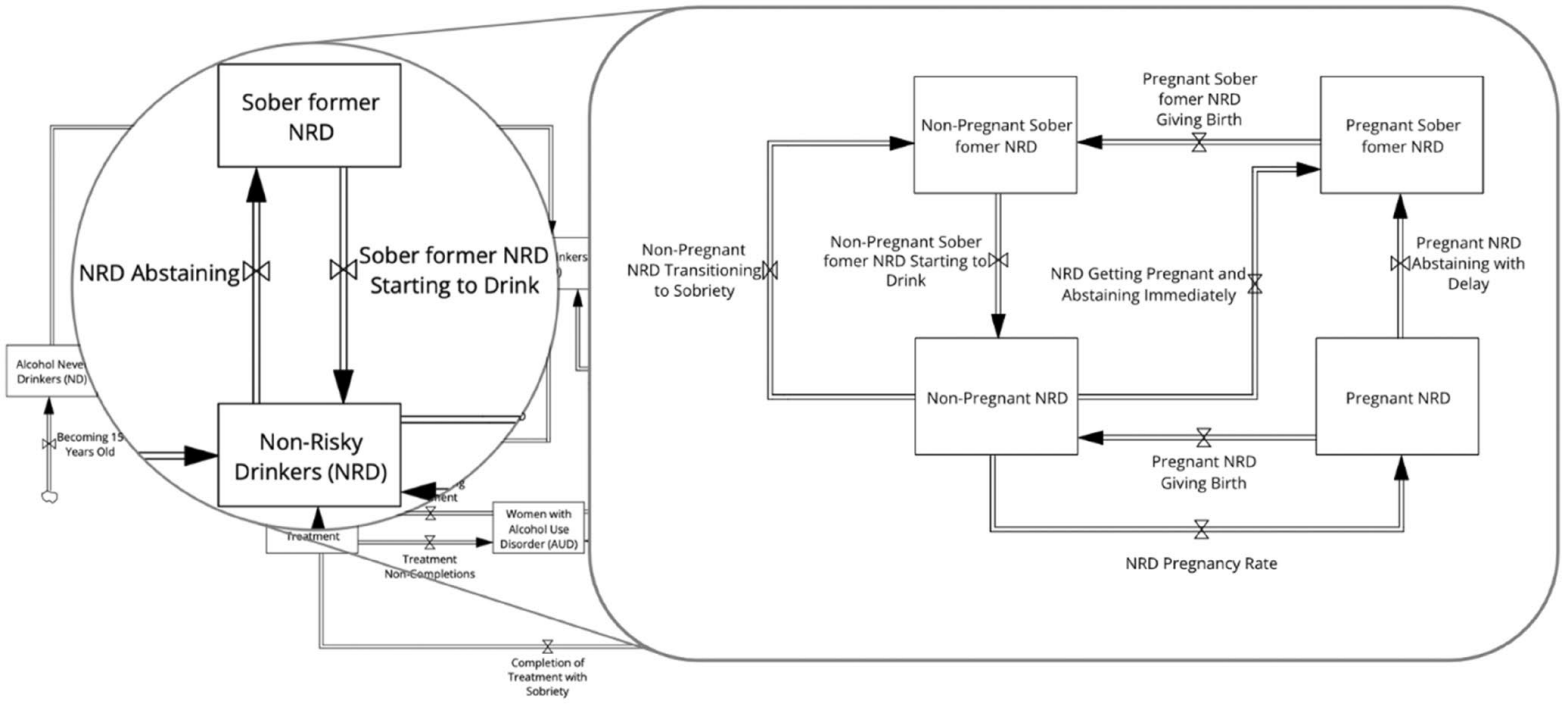
Pregnancy‐specific model structure example with non‐risky drinking alcohol use stage (stock) example, highlighting flow into pregnancy stock (getting pregnant), flow to sobriety stock (stopping drinking during pregnancy), and giving birth outflows. Miscarriage flows not shown for simplicity. Full model diagram and equations are available in the supporting information.

**FIGURE 3:**
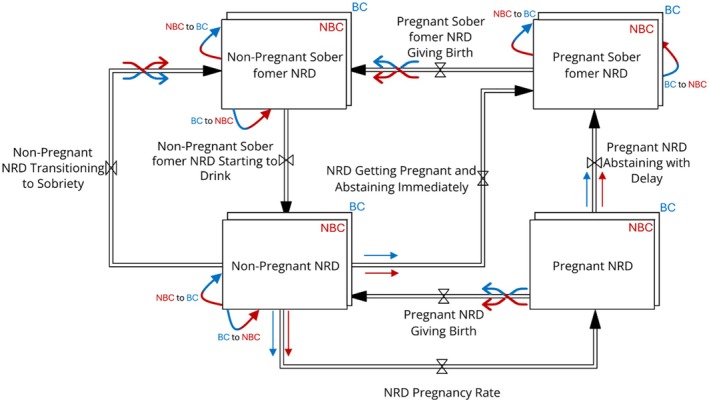
Birth control use‐specific model structures, demonstrating transitions (flows) between birth control use (BC) and non‐birth control use (NBC) stocks, using non‐risky drinking stage (stocks) as an example.

Our model represented population‐level change in pregnancy, alcohol use, and birth control use for women of childbearing age (15–44) within a small metropolitan Northern Plains United States city (the project community site). Historical parameterization was based on data from 2016 to 2019, and the model was simulated until 2028. Each stock in the simulated model represented a specific alcohol use, birth control use, and pregnancy status simultaneously (e.g., a stock representing the number of non‐pregnant, risky drinking women aged 15–44 who do not use birth control). Alcohol use status (Figure 1) included never drinkers, non‐risky drinkers, risky drinkers, and alcohol use disorder (AUD). The model also included corresponding abstaining (former drinking) status (e.g., abstaining non‐risky drinkers, abstaining risky drinkers, abstaining AUD) that represented changes to abstention based on the previous year's drinking behavior, and an AUD treatment status. Birth control use status (Figure 2) was dichotomized to reduce computational complexity and represented either “Birth Control Use” (people consistently using effective birth control or abstaining from penile‐vaginal heterosexual intercourse) or “Non‐Birth Control Use” (people engaging in penile‐vaginal heterosexual intercourse without consistently using effective birth control). An additional model structure represented pregnancy and birth rates (Figure 3), including changes in alcohol use during pregnancy and birth control use after pregnancy.

Our model allowed us to flexibly represent population‐level behavioral change across individual or multiple statuses. For example, we could represent people changing only alcohol use behavior through a flow between stocks with alcohol use statuses and the same birth control use status (e.g., a flow from a risky drinking, non‐birth control use stock to a non‐risky drinking, non‐birth control use stock). A flow between two stocks with different alcohol use and birth control use statuses represented people changing both behaviors. The model also included population dynamic flows (aging in/out, mobility, and mortality). Model structure details, including instructions for replication, can be found in Supporting Information A. The computational model and historical data used can also be accessed from the Open Science Framework in Vensim and Python formats (Deutsch 2025).

### 2.3 Model Data and Quantification

System dynamics models use a combination of varying methods and information to parameterize variables. This can include data from multiple levels of granularity (e.g., individual‐level data such as surveys, community‐level data such as public health aggregates), prior literature, expert opinions, and model calibration. We utilized three different sources of data to parameterize the model, representing within‐person, between‐person, and within‐community change over time. Most stocks were parametrized through an integration of 2016–2019 historical data from relevant national/state datasets on population statistics, behavioral surveillance, or epidemiology. Most flows were parameterized through either alcohol use research (e.g., studies on change in drinking stages over time) or data calibration. Details for historical data calculations, including the specific datasets, variables, and literature used, can be found in Supporting Information A3.

For calibration, we estimated parameters using a Gaussian pseudo log‐likelihood, a widely used working model that focuses on aligning the model's mean trajectories with the data while allowing flexible variance scaling (White 1982; Gourieroux et al. 1984). The objective is to maximize the pseudo log‐likelihood function given the model specifications and target data, which includes 32 time series corresponding to each alcohol drinking stage, pregnancy, and birth control use behavior. Historical data were used to inform data for the model simulations (generated through posterior samples against the target data estimates) and to perform validity and sensitivity tests to build confidence in the simulation (e.g., ensuring that simulated 2016–2019 values match historical data trends). Further detail on calibration approaches can be found in Supporting Information B, including summary statistics on estimated parameters, posterior samples, and posterior predictive checks and goodness‐of‐fit statistics for additional sensitivity tests.

### 2.4 Policy Test Simulations

Policy tests were simulated to reflect a single‐year AEP‐P implementation. Tests involved adding an additional fraction to existing outflows to non‐pregnant risky drinking or AUD non‐birth control use stocks in the year 2020. These fractions represented the additional number of non‐pregnant people who would move from a “high AEP risk” stock (AUD/risky drinking, non‐birth control use stocks) to a “low AEP risk” stock due to AEP‐P participation. BCO change involved increasing outflows from non‐birth control stocks to birth control use stocks (remaining in a risky drinking/AUD stock). AO change involved increasing outflows from AUD or risky drinking stocks to the abstainer or non‐risky drinking stock (remaining in a non‐birth control use stock). BB change involved increasing outflows to both a birth control use stock and a lower risk drinking stock (abstaining or non‐risky drinking).

We calculated 84 policy tests (the fraction added to outflows) conditional upon the total percent of a population changing any behavior (program‐population impact), and which behaviors were changed (behavior‐change group proportions). The “program‐population impact” condition represented the percent of people in the non‐pregnant, non‐birth control use, risky drinking, or AUD stocks who “enrolled” in an AEP‐P and changed their behavior to a low‐risk status. In other words, this condition represented the percent of people who would be considered to have reduced AEP risk based on the aggregate score outcome. Four impacts were examined based on low, moderate, or high program efficacy (20%, 40%, 60%, 80%). The behavior‐change group proportion condition represented the proportion of successful participants in AO, BCO, and BB groups. Twenty‐one behavior‐change group conditions were examined by testing every combination of varying proportions (0–100%, in multiples of 20) for each group from 0% to 100%. This condition represented only the percentage of people who would have changed their behavior. Through this strategy, we were able to examine behavior change outcomes in two different ways. First, the impact of behavior change group proportion distributions (e.g., the distribution of participants in AO, BCO, and BB groups). Second, the total percent change for alcohol use and birth control use behavior across all AEP‐P participants. For example, a policy with AO, BCO, and BB proportions of 20%, 40%, and 40% will result in 60% of all AEP‐P participants changing their alcohol use (AO + BB proportions) and 80% of all AEP‐P participants changing their birth control (BCO + BB proportions). Table 1 provides details on the total percent of change in alcohol use and birth control use for each policy test, breaking down the total percent of alcohol and/or birth control use behavior change (a) within the AEP‐P sample and (b) across the full eligible population within the community. Supporting Information C provides further detail on policy test calculations.

**TABLE 1:** Policy test breakdowns by behavior‐change group proportion and overall program‐population impact on percent change in reducing alcohol and birth control risk behavior for (1) total AEP‐P participant sample and (2) total program‐eligible community population.

Out of total sample of AEP‐P participants successfully reducing AEP risk behavior					Out of total population of people eligible for an AEP‐P within the community							
AO, BCO, and BB proportions (behavior‐change group)			Total % of alcohol and birth control behavior change		Total % of AEP‐P eligible community population changing each behavior (Program‐population impact ☼)							
					20% Impact		40% Impact		60% Impact		80% Impact	
AO%	BCO%	BB%	% ↓Alc	% ↑BC	% ↓Alc	% ↑BC	% ↓Alc	% ↑BC	% ↓Alc	% ↑BC	% ↓Alc	% ↑BC
100%	0%	0%	100%	0%	20%	0%	40%	0%	60%	0%	80%	0%
0%	100%	0%	0%	100%	0%	20%	0%	40%	0%	60%	0%	80%
0%	0%	100%	100%	100%	20%	20%	40%	40%	60%	60%	80%	80%
80%	20%	0%	80%	20%	16%	4%	32%	8%	48%	12%	64%	16%
20%	80%	0%	20%	80%	4%	16%	8%	32%	12%	48%	16%	64%
80%	0%	20%	100%	20%	20%	4%	40%	8%	60%	12%	80%	16%
20%	0%	80%	100%	80%	20%	16%	40%	32%	60%	48%	80%	64%
0%	80%	20%	20%	100%	4%	20%	8%	40%	12%	60%	16%	80%
0%	20%	80%	80%	100%	16%	20%	32%	40%	48%	60%	64%	80%
60%	40%	0%	60%	40%	12%	8%	24%	16%	36%	24%	48%	32%
40%	60%	0%	40%	60%	8%	12%	16%	24%	24%	36%	32%	48%
0%	60%	40%	40%	100%	8%	20%	16%	40%	24%	60%	32%	80%
0%	40%	60%	60%	100%	12%	20%	24%	40%	36%	60%	48%	80%
60%	0%	40%	100%	40%	20%	8%	40%	16%	60%	24%	80%	32%
40%	0%	60%	100%	60%	20%	12%	40%	24%	60%	36%	80%	48%
60%	20%	20%	80%	40%	16%	8%	32%	16%	48%	24%	64%	32%
20%	60%	20%	40%	80%	8%	16%	16%	32%	24%	48%	32%	64%
20%	20%	60%	80%	80%	16%	16%	32%	32%	48%	48%	64%	64%
40%	20%	40%	80%	60%	16%	12%	32%	24%	48%	36%	64%	48%
20%	40%	40%	60%	80%	12%	16%	24%	32%	36%	48%	48%	64%
40%	40%	20%	60%	60%	12%	12%	24%	24%	36%	36%	48%	48%

Abbreviation: ☼ Program‐population impact, percent of people who changed any behavior within total number of the population eligible for the AEP prevention program.

We accounted for differences in alcohol use behavior change as transitions to either non‐risky drinking or alcohol abstention by including a fixed ratio of people moving into either non‐risky drinking or abstainer stocks (75% non‐risky drinking/25% abstainer ratio for risky drinkers and 80%/20% for AUD). This ratio was held constant across all policy tests. Few AEP‐P studies provide information on alcohol use behavior change beyond the risk/non‐risk threshold (Ingersoll et al. 2018; Farrell‐Carnahan et al. 2013) or pretreatment alcohol consumption or AUD as a potential moderator (Sobell et al. 2017). We therefore considered broader research on alcohol use brief intervention outcomes (Tanner‐Smith and Lipsey 2015; Knox et al. 2019) for non‐risky drinking/abstainer ratios. We further tested if this ratio influenced outcomes by comparing the original tests to two alternative policy simulation sets with different non‐risk drinking/abstainer ratios. These tests were performed both to explore the impact of changing alcohol use to either low‐risk drinking or abstinence on AEP prevention and to evaluate the ratio as a confounding variable (e.g., that results were driven more by the ratio than by behavior‐change proportions). Alternative sets included a 50%/50% ratio and a reversed ratio (25%/75% non‐risky drinking/abstainer for risky drinking and 80%/20% for AUD) holding all other policy test components constant. In total, this resulted in 252 policy tests.

### 2.5 Policy Test Outcomes

We evaluated policy tests by calculating the percent difference of new AEP “cases” between each policy test and a baseline simulation in which no AEP‐P was implemented. The data we used (AEP cases) were the number of people in drinking stocks who became pregnant in the year 2021 (flows from nonpregnant to pregnant NRD, RD, and AUD stocks, summing both birth control and non‐birth control use substocks). Percent differences were calculated by subtracting the baseline simulation value from each policy test simulation value, divided by the baseline simulation value. This difference represented the percentage that implementing an AEP‐P (the specific policy test) would increase (positive value) or decrease (negative value) the number of next‐year AEP cases within the community. Three AEP outcomes were evaluated: total AEP cases (people in AUD, risky drinking, and non‐risky drinking stocks who became pregnant), high‐risk AEP cases (people in risky drinking and AUD stocks who became pregnant combined), and low‐risk AEP cases (people in the non‐risky drinking stock who became pregnant). We compared alternative policy tests to the no‐intervention baseline and to the original policy set tests through calculating percent differences.

## 3 Results

### 3.1 Simulated Community Demographic Data

The community represented in our simulation had a population of ~12.5 K to 14 K women aged 15–44 between 2016 and 2019. Simulation output indicated that 68% of the total pregnancies across 2016 and 2019 would be AEP cases (749 out of 1102 pregnancies; see Supporting Information D for outputs), with 56% of these pregnancies from the non‐risky drinking stock. We estimated that approximately 30% of all pregnancies between 2016 and 2019 would be from risky drinkers or people who had AUD. Simulation models estimated that, in 2020, 26% and 34% of non‐pregnant 15‐ to 44‐year‐old women would have reached alcohol and birth control use risk thresholds, respectively. Together, 6% of non‐pregnant women aged 15–44 in the total population would have been eligible for AEP‐P participation in 2020 (approximately 763 people).

### 3.2 AEP‐P Policy Test Performance on Total AEP Cases

Table 2 provides comparisons between all 84 AEP‐P policy test scenarios to a baseline no‐implementation scenario. Values represent the percent difference that each scenario increased or decreased the number of total past‐year drinkers who became pregnant (total AEP cases) 1 year after implementing an AEP‐P. Visual comparisons of all policies can be found in Supporting Information E Figure S1. Compared to baseline, all AEP‐P policy test scenarios resulted in a reduction of the next‐year total AEP. On average (across all behavior‐change proportions), total AEP cases were reduced by −3.65% (SD = 1.05), −6.7% (SD = 1.84), −9.27% (SD = 2.49), and −11.45% (SD = 3.03) for 20%, 40%, 60%, and 80% program‐population impacts, respectively. There was greater variability across behavior‐change proportions at higher levels of program‐population impact. For example, the absolute difference between the highest and lowest performing policies was 4% at 20% impact and 12.08% at 80% impact.

**TABLE 2:** Percent difference comparing simulation outputs for baseline status‐quo and each AEP‐P policy test for the number of total drinkers becoming pregnant (total AEP cases) in 2021 (e.g., percent decrease or increase in the number of all drinkers getting pregnant for each AEP‐P policy implemented compared to baseline).

AO/BCO/BB proportion			20% Program population impact	40% Program population impact	60% Program population impact	80% Program population impact	Total % of participants changing risky alcohol and birth control use	
AO%	BCO%	BB%					Alc. Use	BC. Use
100%	0%	0%	**−1.59**	**−2.94**	**−4.08**	**−5.05**	100%	0%
0%	100%	0%	−3.75	−6.93	−9.61	−11.89	0%	100%
0%	0%	100%	**−5.58**	**−10.15**	**−13.94**	**−17.13**	100%	100%
80%	20%	0%	*−2.01*	*−3.72*	*−5.21*	*−6.43*	80%	20%
20%	80%	0%	−3.33	−6.15	−8.49	−10.51	20%	80%
80%	0%	20%	−2.44	−4.56	−6.45	−8.09	100%	20%
20%	0%	80%	*−4.83*	*−8.89*	*−12.31*	*−15.25*	100%	80%
0%	80%	20%	−4.10	−7.47	−10.22	−12.53	20%	100%
0%	20%	80%	*−5.17*	*−9.39*	*−12.87*	*−15.76*	80%	100%
60%	40%	0%	*−2.43*	*−4.55*	*−6.29*	*−7.81*	60%	40%
40%	60%	0%	−2.91	−5.32	−7.41	−9.14	40%	60%
0%	60%	40%	−4.45	−8.04	−11.00	−13.42	40%	100%
0%	40%	60%	−4.78	−8.70	−11.88	−14.52	60%	100%
60%	0%	40%	−3.26	−6.11	−8.58	−10.78	100%	40%
40%	0%	60%	−4.06	−7.54	−10.54	−13.14	100%	60%
60%	20%	20%	−2.84	−5.27	−7.41	−9.21	80%	40%
20%	60%	20%	−3.70	−6.72	−9.30	−11.41	40%	80%
20%	20%	60%	−4.42	−8.10	−11.23	−13.83	80%	80%
40%	20%	40%	−3.64	−6.74	−9.40	−11.67	80%	60%
20%	40%	40%	−4.02	−7.41	−10.18	−12.56	60%	80%
40%	40%	20%	−3.24	−6.02	−8.34	−10.33	60%	60%
Mean Difference (SD)			−3.65 (1.02)	−6.70 (1.84)	−9.27 (2.49)	−11.45 (3.03)		
Policy performance absolute difference			3.99	7.21	9.86	12.08		

*Note:* Top three worst policies: Bold red numbers indicate worst performing policy; italicized red numbers indicate second and third worst policy. Top three best policies: Bold green numbers indicate worst performing policy; italicized green numbers indicate second and third worst policy. “Above average” (better) performing policies indicate policies that have values that are more negative than the mean (AEP‐P provides greater reduction in AEP compared to baseline).

High‐BB proportion policies fared the best (greatest reductions). The 100% BB policy was the best performing policy, and all policies with at least 60% BB proportions resulted in higher than average reductions. Comparatively, high‐AO proportion policies fared the worst (lowest reductions). The 100% AO policy was the worst performing policy, and all policies with at least 60% AO resulted in lower than average reductions. For example, 60% BB proportion policies at 40% program‐population impact reduced a higher than average amount of AEP cases (an average of −6.7%) regardless of behavior‐change proportions for the remaining 40% of participants. However, 60% BB policies fared the best if remaining participants were all BCO (40% BCO, −8.7%) compared to equally distributed (20% AO and BCO, at −8.1%) or if they were all AO (40% AO, −7.41%).

The 100% BCO policy resulted in higher than average reductions; however, reductions for high‐BCO policies depended upon the behavior‐change group proportions of the remaining distribution. For example, consider an AEP‐P program with a 40% program‐population impact in which 80% of participants changed only their birth control use behavior (80% BCO). If the remaining 20% of participants changed both behaviors (20% BB), next‐year AEP cases would be 7.47% lower than if no program was implemented (higher than the average 40% program‐population impact policy). However, if the remaining 20% of participants changed only alcohol use (20% AO), next year AEP cases would only be lower by 6.15% (lower than the average 40% program‐population impact policy). The importance of changing birth control use behavior, compared to alcohol use behavior, was also seen in the impact of policies on the total percent of behavior changed. Policy tests in which 80–100% of total AEP‐P participants changed birth control use almost always resulted in a higher than average reduction, even if the total percent of AEP‐P participants who changed alcohol use was relatively low (e.g., low BB proportion). However, policy tests in which 80–100% of total AEP‐P participants changed alcohol use did not result in higher than average reductions unless the total percent of people who changed birth control use was also high (at least 60%, indicating a higher BB proportion).

There was overlap between higher and lower performing policies at different impact levels, such that higher performing policies at lower rates of impact had similar reductions as lower performing policies at higher rates of impact. For example, efforts to reduce new cases of AEP‐P by at least 10% would require the best performing policy (100% BB) if 40% of eligible community members enrolled in an AEP‐P and successfully changed AEP‐risk behavior. However, reducing AEP by 10% could be achieved with 9 or 15 policies if the percent of eligible community members enrolled in an AEP‐P and successfully changed AEP‐risk behavior increased to 60% or 80%, respectively.

### 3.3 AEP‐P Policy Test Performance on AEP Cases by Risk Threshold

#### 3.3.1 High‐Risk AEP: Risky Drinking People or People With AUD Becoming Pregnant

Table 3 values represent the percent increase or decrease in the number of risky drinkers and AUD drinkers who would become pregnant (high‐risk AEP) if an AEP‐P was implemented. All policy tests reduced risky AEP cases. Supporting Information E Figure S2 provides visual comparisons. On average, reductions at 20%, 40%, 60%, and 80% program‐population impacts were −11.36% (SD = 2.28), −20.37% (SD = 3.63), −29.53% (SD = 4.36), and −33.27% (SD = 4.68), respectively. Similar to the total AEP outcomes, policy performance ranges were larger at higher program‐population impact levels. High‐BB policies also performed similar to the total AEP outcomes, such that the 100% BB policy test resulted in the highest reductions. There was also overlap in high‐risk AEP reduction for higher and lower performing policies at different impact levels. For example, efforts to reduce high‐risk AEP by 25% would require a top‐three performing program at a 40% program‐population impact, but could be achieved with 14 policies at 60% impact, and all 21 policies at 80% impact.

**TABLE 3:** Percent difference comparing simulation outputs for baseline status‐quo and each AEP‐P policy test for the number of risky drinking and women with AUD becoming pregnant (risky/AUD AEP cases) in 2021 (e.g., percent decrease or increase in the number of risky drinking and AUD people getting pregnant for each AEP‐P policy implemented compared to baseline).

AO/BCO/BB proportion			20% Program population impact	40% Program population impact	60% Program population impact	80% Program population impact	Total % of participants changing risky alcohol and birth control use	
AO%	BCO%	BB%					Alc. Use	BC. Use
100%	0%	0%	−9.30	−17.17	−23.83	−29.46	100%	0%
0%	100%	0%	**−8.26**	**−15.23**	**−21.10**	**−26.06**	0%	100%
0%	0%	100%	**−16.27**	**−27.95**	**−36.35**	**−42.43**	100%	100%
80%	20%	0%	−9.10	−16.79	−23.28	−28.78	80%	20%
20%	80%	0%	*−8.47*	*−15.61*	*−21.65*	*−26.74*	20%	80%
80%	0%	20%	−10.80	−19.63	−26.88	−32.82	100%	20%
20%	0%	80%	*−14.97*	*−26.08*	*−34.33*	*−40.50*	100%	80%
0%	80%	20%	−9.96	−18.09	−24.73	−30.15	20%	100%
0%	20%	80%	*−14.77*	*−25.71*	*−33.82*	*−39.88*	80%	100%
60%	40%	0%	−8.89	−16.39	−22.74	−28.10	60%	40%
40%	60%	0%	*−8.67*	*−16.01*	*−22.19*	*−27.42*	40%	60%
0%	60%	40%	−11.61	−20.79	−28.04	−33.79	40%	100%
0%	40%	60%	−13.22	−23.32	−31.07	−37.01	60%	100%
60%	0%	40%	−12.24	−21.93	−29.62	−35.74	100%	40%
40%	0%	60%	−13.63	−24.08	−32.10	−38.28	100%	60%
60%	20%	20%	−10.59	−19.25	−26.33	−32.15	80%	40%
20%	60%	20%	−10.17	−18.48	−25.26	−30.82	40%	80%
20%	20%	60%	−13.43	−23.71	−31.58	−37.64	80%	80%
40%	20%	40%	−12.03	−21.56	−29.09	−35.08	80%	60%
20%	40%	40%	−11.83	−21.17	−28.57	−34.43	60%	80%
40%	40%	20%	−10.39	−18.86	−25.80	−31.48	60%	60%
Mean difference (SD)			−11.36 (2.28)	−20.37 (3.63)	−29.53 (4.36)	−33.27 (4.68)		
Policy performance absolute difference			8.01	12.72	14.94	15.96		

*Note:* Top three worst policies: Bold red numbers indicate worst performing policy; italicized red numbers indicate second and third worst policy. Top three best policies: Bold green numbers indicate worst performing policy; italicized green numbers indicate second and third worst policy. “Above average” (better) performing policies indicate policies that have values that are more negative than the mean (AEP‐P provides greater reduction in AEP compared to baseline).

There were two key differences in policy tests performance for high‐risk AEP, compared to total AEP cases. First, the BCO behavior‐change group was associated with the lowest levels of high‐risk AEP reduction (e.g., the 100% BCO policy resulted in the lowest reduction). Second, for the high‐risk AEP outcome, high‐AO and high‐BCO policies both performed comparatively worse than high‐BB policies. All policies in which proportions of AO or BCO were higher than BB resulted in below‐average reductions. Although some policies with 20% or lower BB resulted in higher‐than‐average reductions for total AEP, policies only resulted in above‐average reductions of high‐risk AEP if the policy included at least a 40% BB group. These differences were also reflected when examining the total percent of people changing each behavior. Above‐average reductions for total AEP were possible for multiple policies in which the total percent of alcohol use behavior change was low (0%–40%), as long as these policies involved a high percentage of total participants changing birth control use (e.g., 80%–100%). However, policies would only have a higher than average reduction of high‐risk AEP if the total sum of all behavior change was 140% or higher (e.g., if the total sample changed at least 60% of one behavior and at least 80% of the other behavior, through a combination of AO, BCO, and BB proportions).

#### 3.3.2 Low‐Risk AEP: Non‐Risky Drinkers Becoming Pregnant

Table 4 values represent the percent increase or decrease in the number of non‐risky drinkers (low‐risk AEP) who would become pregnant if an AEP‐P was implemented. Supporting Information E Figure S3 provides visual comparisons. Results indicated that policies performed very differently for the ways in which they impacted low‐risk AEP, compared to high‐risk AEP. Every single AEP‐P policy test scenario, except the 100% BCO policy, resulted in an increase for next‐year low‐risk AEP‐P. The 100% AO policy was the worst performing (resulting in the highest increase of non‐risky AEP). Thus, with the exception of the 100% BCO policy, policy performance was based on which policies resulted in the lowest increases. On average, the policy test baseline percent differences at 20%, 40%, 60%, and 80% program‐population impacts were 2.26% (SD = 1.18), 3.76% (SD = 2.08), 4.71% (SD = 2.84), and 5.25% (SD = 3.56).

**TABLE 4:** Percent difference comparing simulation outputs for baseline status‐quo and each AEP‐P policy test for the number of non‐risky drinkers becoming pregnant (non‐risky AEP cases) in 2021 2021 (e.g., percent decrease or increase in the number of non‐risky drinking people getting pregnant for each AEP‐P policy implemented compared to baseline).

AO/BCO/BB proportion			20% Program population impact	40% Program population impact	60% Program population impact	80% Program population impact	Total % of participants changing risky alcohol and birth control use	
AO%	BCO%	BB%					Alc. Use	BC. Use
100%	0%	0%	**4.31**	**7.96**	**11.03**	**13.63**	100%	0%
0%	100%	0%	**−0.31**	**−0.58**	**−0.83**	**−1.05**	0%	100%
0%	0%	100%	2.61	3.47	3.21	2.23	100%	100%
80%	20%	0%	3.41	6.29	8.62	10.68	80%	20%
20%	80%	0%	*0.60*	*1.09*	*1.58*	*1.90*	20%	80%
80%	0%	20%	*3.96*	*6.97*	*9.18*	*10.83*	100%	20%
20%	0%	80%	2.93	4.26	4.54	4.07	100%	80%
0%	80%	20%	*0.39*	*0.65*	*0.88*	*0.95*	20%	100%
0%	20%	80%	2.17	3.10	3.16	2.70	80%	100%
60%	40%	0%	2.51	4.52	6.30	7.72	60%	40%
40%	60%	0%	1.50	2.85	3.90	4.85	40%	60%
0%	60%	40%	1.03	1.72	2.03	2.16	40%	100%
0%	40%	60%	1.68	2.49	2.80	2.70	60%	100%
60%	0%	40%	*3.61*	*6.00*	*7.52*	*8.32*	100%	40%
40%	0%	60%	3.26	5.12	5.95	6.10	100%	60%
60%	20%	20%	3.09	5.44	7.07	8.34	80%	40%
20%	60%	20%	1.26	2.28	2.92	3.44	40%	80%
20%	20%	60%	2.47	3.84	4.35	4.40	80%	80%
40%	20%	40%	2.78	4.60	5.67	6.25	80%	60%
20%	40%	40%	1.96	3.12	3.89	4.18	60%	80%
40%	40%	20%	2.23	3.81	5.03	5.85	60%	60%
Mean Difference (SD)			2.26 (1.18)	3.76 (2.08)	4.71 (2.84)	5.25 (3.56)		
Policy performance absolute difference			4.01	7.38	10.21	12.59		

*Note:* Top three worst policies: Bold red numbers indicate worst performing policy; italicized red numbers indicate second and third worst policy. Top three best policies: Bold green numbers indicate worst performing policy; italicized green numbers indicate second and third worst policy. “Above average” (better) performing policies indicate policies that have values that are more negative than the mean (AEP‐P provides greater reduction in AEP compared to baseline).

Overall, policies performed better (resulted in lower increases for non‐risky AEP) if they included less participants engaging in alcohol‐related behavior change. Policies that were related to the highest increases in non‐risky AEP‐P were those in which 80% of total participants changing their alcohol use, or a larger percent of total AEP‐P participants changed alcohol use compared to birth control use. This held even for policies in which the total percent of people changing birth control was also high (80% or 100%) through higher proportions of people changing both behaviors.

Furthermore, the program‐population impact level appeared uniquely relate to policy performance for low‐risk AEP outcomes. Policies with high percentages of people who changed their birth control use were more likely to “cluster” with each other regardless of impact level (see Supporting Information E Figure S4). For example, the 80% BCO, 20% BB policy (with 100% and 20% of the total sample changing birth control and alcohol use, respectively), has relatively low variance across impact levels with an absolute difference of 0.56%. In addition, the effect of a policy on non‐risky AEP was not always linearly related to program impact. For example, the 100% BB policy had a higher increase in non‐risky AEP, at 40% program‐population impact, compared to 60% or 80%.

### 3.4 Alternative Abstainer/Non‐Risk Drinking Ratios for Reducing Alcohol Use Risk Behavior

Finally, we examined the role of alcohol‐use change participants transitioning to either sobriety or non‐risky drinking by modifying the fixed abstainer/non‐risk ratio (25%/75% abstainer/non‐risk ratio for the risky drinking stock and a 20%/80% abstainer/non‐risk ratio for the AUD stock). The alternative sets changed the abstainer/non‐risk drinking ratio to 50/50 or to a reversed ratio (75/25 abstainer/non‐risk for the risky drinking stock and 80/20 for the AUD stock). Baseline‐policy test percent differences for each policy test set are found Supporting Information E Figure S4a–d, Figure S5a–d, and Figure S6a–d for total AEP, risky AEP, and non‐risky AEP outcomes respectively. Increasing the proportion of AEP‐P who changed their alcohol use to abstinence resulted in lower rates of new cases of non‐risky AEP (but not risky AEP) compared to the original policy tests. For policy tests with high proportions of participants changing alcohol use (especially AO), the alternative ratio tests resulted in much lower increases for next‐year cases of non‐risky AEP. However, most policies (19 of 21) continued to result in increased non‐risky AEP compared to the baseline.

Percent differences between the original alternative policy tests can be found in Supporting Information E Tables S1–S3 (total, high‐risk, and non‐risky drinking AEP, respectively). Negative numbers indicated that the alternative ratio policy tests resulted in a greater reduction of AEP compared to the original policy tests. Policies that had higher birth control use change had much more similar outcomes across all three ratio sets. For example, as seen in Supporting Information E Table S3 (non‐risky AEP outcomes), there was a greater percent difference (with negative numbers indicating a reduction in AEP, or alternatively, a weaker increase in non‐risky AEP) for policies that had higher proportions of either AO or BB.

## 4 Discussion

AEP remains a global public health concern (Popova and Dozet 2023) that can be best addressed through preconceptual prevention, the end goal of AEP‐P. We used the flexibility of a simulation approach to examine the association between behavioral changes that are promoted by AEP‐P and prevention of AEP itself. Specifically, we examined whether the three potential behavior‐change outcomes from AEP‐P changing AO, BCO, or BB to below‐risk threshold (Floyd et al. 2007) equally prevent AEP. As the main outcome variable used to demonstrate AEP‐P efficacy involves aggregating these three outcomes, testing the underlying assumption that all three behavior‐change outcomes equally reduce risk for AEP can inform our understanding of AEP‐P as a prevention program. Our results indicated that this assumption does not hold; AEP‐P performance varied as a function of behavior‐change group proportions. Although all policies resulted in reduced high‐risk AEP (the target prevention outcome), the percent difference in reduction between the lowest and highest performing policies ranged from 8% to 16% (based on program‐population impact).

The BB change was associated with the highest reductions in total and high‐risk AEP, while the BCO group was associated with the lowest high‐risk reductions. Although AO change was associated with the lowest total AEP reductions, this was driven by increases in low‐risk AEP, especially if AO (and BB) participants changed their alcohol use behavior to low‐risk drinking compared to abstinence. Conversely, the BCO group was associated with the lowest increases in low‐risk AEP. It should be noted that, while most policies increased low‐risk AEP, this increase could also be considered a shift in pregnancies from high to low risk AEP (which can reduce (Dejong et al. 2019) but potentially not eliminate (Sundermann et al. 2019) negative AEP effects on pregnancy and infant outcomes). Taken together, BB change (especially when coupled with abstinence‐related alcohol use change) appears to provide the “best” reduction across both high‐ and low‐risk AEP.

Examining current study results within the context of previous AEP‐P study outcomes suggests that the aggregate, binary AEP risk behavior variable may bias estimations of program effects for actual risk reduced and high‐risk AEP prevented. Specifically, this bias may involve overestimation of prevention compared to risk reduction. Prior study effects are likely represented by policy tests that include 40%–80% program‐population impacts (e.g., the total percent of AEP‐P participants reducing AEP risk) (Sobell et al. 2017; Wilton et al. 2013) and 20%–40% BB, 20%–60% BCO (Floyd et al. 2007; Hutton et al. 2014) proportions. Although policies within this range often resulted in overall AEP reductions that were higher than average, they had lower than average reductions for high‐risk AEP. The original CHOICES studies reported higher BB proportions (Floyd et al. 2007, Group* 2003). However, subsequent studies more frequently reported that more participants who reduced AEP risk increased their birth control use compared to reducing alcohol use (Ceperich and Ingersoll 2011; Hanson et al. 2017; Sobell et al. 2017). Studies that report higher BCO proportions therefore may have lower levels of actual AEP prevention.

### 4.1 Limitations

The current study has several limitations. First, comparisons between AEP‐P evaluations and the current study must be done carefully. While AEP‐P research traditionally utilizes a frequentist statistical approach based on a sample within a population, the current simulations use a nonlinear systems approach based on a full population sample. The simulation was grounded in data representing a specific target community, and results may not generalize to other areas. Policy tests did not account for birth control types, which may have impacted results (e.g., long‐acting reversible birth control vs. condoms). Our model used historical data that stopped prior to 2020, given lower availability and accessibility of post‐2020 data from some parameterization data sources (e.g., Behavior Risk Factor Surveillance System family planning module). It is possible that extending the model beyond 2020 would have provided different results. We examined this by comparing 2018–2019 to 2021–2022 and 2022–2023 restricted National Survey on Drug Use and Health datasets for 15‐ to 44‐year‐old non‐pregnant women using the same population criteria (one of our parametrization datasets). We saw minimal change in reporting ever drinking (86%, 85%, 83%), past year drinking (89%, 91%, 89%), and slight increase and decrease in past month binge drinking (37%, 39%, 34%) for this sample. There was also little change in trends for state PRAMS participants between 2018 and 2021 who reported any drinking (67%) or binge drinking (41%–39%) 3 months before pregnancy. Finally, this study only examined a single model structure without reserved validation data, and these limitations may have resulted in overfitting to the target data. Additional simulations (in tandem with future AEP‐P research that improves outcome reporting, see below) can help discern assurance of the current findings.

### 4.2 Future Directions

Although the current study suggests that AEP‐P research can benefit from strategies to strengthen BB outcomes, the first step of this process involves strengthening the research. Identifying strategies to improve AEP‐P will require more comprehensive information from the intervention research studies themselves. Using behavior‐change groups as an additional outcome variable may provide a more rigorous examination of efficacy compared to the binary risk variable or individual behaviors. Furthermore, understanding strategies for improving AEP‐P (e.g., including more sessions, additional modules, or participant support) would need to be balanced with the value that a low‐cost, low‐burden intervention can have for low‐resource communities. Using behavior‐change group proportions as a main outcome for evaluation may help identify cost–benefit balance strategies. For example, results of the current study suggested that higher performing policies would require a smaller number of participants to reduce similar numbers of next‐year AEP cases. Therefore, a more resource‐intensive program that promotes a larger BB proportion may be possible if it is provided to a smaller sample. Another strategy may be to develop “target proportions” for BB change as a metric of efficacy beyond the binary aggregate risk variable. Developing an AEP in which all participants change both behaviors and change their alcohol use to abstinence is unrealistic. However, developing realistic behavior‐change proportion goals (e.g., increasing BB by 10%) could be used as a potential benchmark for incremental improvement.

To build on existing knowledge for further improvement of AEP‐P, future research should examine more comprehensive or detailed behavior change outcome measures. Studies that include outcomes such as what types of birth control people select or different alcohol use behaviors beyond the binary threshold could help interventionists identify strategies to improve AEP‐P curricula; for example, if birth control use change focused on long‐acting reversible contraception, it is likely that participants would have similar prevention outcomes if they changed only birth control or if they changed both behaviors. Studies would also benefit from additional exploratory analyses for moderating factors (and more measurement of additional potential moderators), which could help clarify potential reasons for differences in participant change of birth control or alcohol use behavior. For example, secondary qualitative analyses of AEP‐P interventions have indicated that romantic partners or friends may pose as contextual barriers for alcohol use behavior change (Deutsch et al. 2021; Hernandez et al. 2023). Other comorbid or contextual factors, such as the use of other substances (Wilton et al. 2013), mental health conditions (Johnson et al. 2017), or baseline severity of problematic alcohol use (Sobell et al. 2017), are commonly noted challenges for treatment success across the larger body of alcohol use research. Although some of these variables (especially substance‐related variables, such as AUD or illicit drug use) are often controlled in AEP‐P studies (Ingersoll et al. 2018; Wilton et al. 2013), they are rarely explored as moderators. These additional data could also be used for secondary analyses to examine both changes for each behavior (Johnson et al. 2017, VON Sternberg et al. 2023) and behavior‐change groups. Taken together, more rigorous evaluations of AEP‐P with detailed information on people's behavior change and the broader barriers or facilitators for this change will help interventionists consider specific, targeted strategies to enhance existing programs without greatly expanding the resources required to implement them.

## Funding

This work was supported by the National Institute on Drug Abuse, R01 DA050696.

## Conflicts of Interest

The authors declare no conflicts of interest.

## Supporting information


**Data S1:** acer70379‐sup‐0001‐Supinfo.docx. **Supporting Information E Figure S1**. Percent difference comparing simulation outputs for baseline status‐quo and each AEP‐P policy test for the number of total drinkers becoming pregnant (total AEP cases) in 2021 (e.g., percent decrease or increase in the number of all drinkers getting pregnant for each AEP‐P policy implemented compared to baseline).
**Supporting Information: E Figure S2** Percent difference comparing simulation outputs for baseline status‐quo and each AEP‐P policy test for the number of risky drinking and women with AUD becoming pregnant (Risky/AUD AEP Cases) in 2021 (e.g., percent decrease or increase in the number of risky drinking and AUD people getting pregnant for each AEP‐P policy implemented compared to baseline).
**Supporting Information: E Figure S3** Percent difference comparing simulation outputs for baseline status‐quo and each AEP‐P policy test for the number of non‐risky drinkers becoming pregnant (non‐risky AEP cases) in 2021 (e.g., percent decrease or increase in the number of non‐risky drinking people getting pregnant for each AEP‐P policy implemented compared to baseline).
**Supporting Information: E Figure S4a** Percent difference in simulation outputs for next‐year number of all non‐pregnant women drinkers aged 15–44 becoming pregnant in 2021, comparing baseline non‐intervention (status‐quo) baseline simulation and both original and alternative policy test simulations, at 20% program population impact.
**Supporting Information: E Figure S4b** Percent difference in simulation outputs for next‐year number of all non‐pregnant women drinkers aged 15–44 becoming pregnant in 2021, comparing baseline non‐intervention (status‐quo) baseline simulation and both original and alternative policy test simulations, at 40% program population impact.
**Supporting Information: E Figure S4c** Percent difference in simulation outputs for next‐year number of all non‐pregnant women drinkers aged 15–44 becoming pregnant in 2021, comparing baseline non‐intervention (status‐quo) baseline simulation and both original and alternative policy test simulations, at 60% program population impact.
**Supporting Information: E Figure S4d** Percent difference in simulation outputs for next‐year number of all non‐pregnant women drinkers aged 15–44 becoming pregnant in 2021, comparing baseline non‐intervention (status‐quo) baseline simulation and both original and alternative policy test simulations, at 80% program population impact.
**Supporting Information: E Figure S5a** Percent difference in simulation outputs for next‐year number of all non‐pregnant risky drinking and AUD women aged 15–44 becoming pregnant in 2021, comparing baseline non‐intervention (status‐quo) baseline simulation and both original and alternative policy test simulations, at 20% program population impact.
**Supporting Information: E Figure S5b** Percent difference in simulation outputs for next‐year number of all non‐pregnant risky drinking and AUD women aged 15–44 becoming pregnant in 2021, comparing baseline non‐intervention (status‐quo) baseline simulation and both original and alternative policy test simulations, at 40% program population impact.
**Supporting Information: E Figure S5c** Percent difference in simulation outputs for next‐year number of all non‐pregnant risky drinking and AUD women aged 15–44 becoming pregnant in 2021, comparing baseline non‐intervention (status‐quo) baseline simulation and both original and alternative policy test simulations, at 60% program population impact.
**Supporting Information: E Figure S5d** Percent difference in simulation outputs for next‐year number of all non‐pregnant risky drinking and AUD women aged 15–44 becoming pregnant in 2021, comparing baseline non‐intervention (status‐quo) baseline simulation and both original and alternative policy test simulations, at 80% program population impact.
**Supporting Information: E Figure S6a** Percent difference in simulation outputs for next‐year number of all non‐pregnant non‐risky drinking women aged 15–44 becoming pregnant in 2021, comparing baseline non‐intervention (status‐quo) baseline simulation and both original and alternative policy test simulations, at 20% program population impact.
**Supporting Information: E Figure S6b** Percent difference in simulation outputs for next‐year number of all non‐pregnant non‐risky drinking women aged 15–44 becoming pregnant in 2021, comparing baseline non‐intervention (status‐quo) baseline simulation and both original and alternative policy test simulations, at 40% program population impact.
**Supporting Information: E Figure S6c** Percent difference in simulation outputs for next‐year number of all non‐pregnant non‐risky drinking women aged 15–44 becoming pregnant in 2021, comparing baseline non‐intervention (status‐quo) baseline simulation and both original and alternative policy test simulations, at 60% program population impact.
**Supporting Information: E Figure S6d** Percent difference in simulation outputs for next‐year number of all non‐pregnant non‐risky drinking women aged 15–44 becoming pregnant in 2021, comparing baseline non‐intervention (status‐quo) baseline simulation and both original and alternative policy test simulations, at 80% program population impact.
**Supporting Information: E Table S1** Percent difference comparing simulation outputs for original AEP‐P policy tests (baseline) and alternative sober/non‐risky drinking ratio AEP‐P policy tests for the number of total non‐pregnant drinking women aged 15–44 becoming pregnant (Total AEP Cases) in 2021.
**Supporting Information: E Table S2** Percent difference comparing simulation outputs for original AEP‐P policy tests (baseline) and alternative sober/non‐risky drinking ratio AEP‐P policy tests for the number of non‐pregnant risky drinking and AUD women aged 15–44 becoming pregnant (risky drinking and AUD AEP Cases) in 2021.
**Supporting Information: E Table S3** Percent difference comparing simulation outputs for original AEP‐P policy tests (baseline) and alternative sober/non‐risky drinking ratio AEP‐P policy tests for the number of total non‐pregnant non‐risky drinking women aged 15–44 becoming pregnant (non‐risky AEP cases) in 2021.

**Data S2:** Supporting Information.

**Data S3:** Stock names: NBC: non‐birth control use; BC: Birth control use; RD Risky drinking (alcohol use stock); AUD: Alcohol use disorder (alcohol use stock); NRD: Non‐risky drinking (alcohol use stock).

**Data S4:** Supporting Information.

## Data Availability

Publicly available data sources for model parameterization, model and policy test equations, and simulation output data that supports the findings of this study are available in the supporting information of this article. Data and simulation model are available at https://github.com/MJ%E2%80%90LAB%E2%80%90Harvard/alcohol%E2%80%90exposed%E2%80%90pregnancy%E2%80%90simulation.
