# Supplementary material for: Linking Reductions in Alcohol and Birth Control Use Risk Behavior to Prevention of Alcohol‐Exposed Pregnancy: A Population‐Level Simulation of Preconceptual Prevention Programs
Source: Alcohol Clin Exp Res (Hoboken). 2026 Jul 22;50(7):e70379. doi: 10.1111/acer.70379 (PMC13392302; doi:10.1111/acer.70379)
Supplement: Supplementary file 1 — Data S1: acer70379‐sup‐0001‐Supinfo.docx. Supporting Information E Figure S1. Percent difference comparing simulation outputs for baseline status‐quo and each AEP‐P policy test for the number of total drinkers becoming pregnant (total AEP cases) in 2021 (e.g., percent decrease or increase in the number of all drinkers getting pregnant for each AEP‐P policy implemented compared to baseline). Supporting Information: E Figure S2 Percent difference comparing simulation outputs for baseline status‐quo and each AEP‐P policy test for the number of risky drinking and women with AUD becoming pregnant (Risky/AUD AEP Cases) in 2021 (e.g., percent decrease or increase in the number of risky drinking and AUD people getting pregnant for each AEP‐P policy implemented compared to baseline). Supporting Information: E Figure S3 Percent difference comparing simulation outputs for baseline status‐quo and each AEP‐P policy test for the number of non‐risky drinkers becoming pregnant (non‐risky AEP cases) in 2021 (e.g., percent decrease or increase in the number of non‐risky drinking people getting pregnant for each AEP‐P policy implemented compared to baseline). Supporting Information: E Figure S4a Percent difference in simulation outputs for next‐year number of all non‐pregnant women drinkers aged 15–44 becoming pregnant in 2021, comparing baseline non‐intervention (status‐quo) baseline simulation and both original and alternative policy test simulations, at 20% program population impact. Supporting Information: E Figure S4b Percent difference in simulation outputs for next‐year number of all non‐pregnant women drinkers aged 15–44 becoming pregnant in 2021, comparing baseline non‐intervention (status‐quo) baseline simulation and both original and alternative policy test simulations, at 40% program population impact. Supporting Information: E Figure S4c Percent difference in simulation outputs for next‐year number of all non‐pregnant women drinkers aged 15–44 becoming pregnant in 2021, c [file ACER-50-0-s004.docx]

***Supplements A-B: Methodological Appendix for***

**Linking reductions in alcohol and birth control use risk behavior to prevention of alcohol-exposed pregnancy: A population-level simulation of preconceptual prevention programs**

**Contents**

[Introduction: Overview of Supplementary Materials and Model Documentation 2](#_Toc228106030)

[Supplement A1. Replication Instructions 4](#_Toc228106031)

[Supplement A2. Model Structure 5](#_Toc228106032)

[Supplement A3. Model parameterization 10](#_Toc228106033)

[Dataset-Informed Parameterization 10](#_Toc228106034)

[Literature-Informed Parameterization 16](#_Toc228106035)

[Supplement B1. Model calibration 22](#_Toc228106036)

[Supplement B2. Estimated parameters and posterior samples 27](#_Toc228106037)

[Supplement B3. Posterior predictive checks and fit to data 33](#_Toc228106038)

[References 43](#_Toc228106039)

# Introduction: Overview of Supplementary Materials and Model Documentation

The supplementary materials associated with this study document the model in a way that allows readers to understand how it was parameterized and estimated and how the reported results can be reproduced. These materials are distributed across companion supplement files rather than presented in a single appendix. Read together with the public repository of the model files, they are intended to give readers direct access to the model structure, operational definitions, data inputs, parameter values, estimation outputs, fit statistics, simulation files, and policy results, following the reporting guidelines recommended for system dynamics models for full transparency and reproducibility (Rahmandad & Sternman, 2012). In other words, all model equations, state definitions, transition functions, and parameter values are explicitly reported, allowing the model to be independently reconstructed, interrogated, and extended without reliance on implicit or undocumented processes.

Specifically, the supplementary contents include:

- **Supplement A1**: Replication instructions and model access
- **Supplement A2**: Model structure and stock definitions (Table A2.1)
- **Supplement A3**: Data sources and parameterization (Tables A3.1-A3.4)
- **Supplement B1**: Model calibration and parameter priors (Table B1.1)
- **Supplement B2**: Posterior parameter estimates (Table B2.1; Figure B2.1)
- **Supplement B3**: Model fit and posterior predictive checks (Figure B3.1; Tables B3.1-2)

Additional supplementary materials, not included in this text, are:

- **Supplement C-D**: Simulation outputs for policy experiments
- **Supplement E**: Policy results (Supplemental Tables 1-2; Supplemental Figures E.1-6d)

Supplement A1 provides replication instructions and access to the model in both its original system dynamics form (Vensim software) and a code-based implementation (Python). This section serves as the entry point for readers who want to run the model, inspect variables, and reproduce example outputs.

Supplement A2 documents how the model is constructed and grounded in data. Specifically, it defines the model state variables and transition rates, including pregnancy status, alcohol use stages, abstention states, treatment states, and birth control use (Table A2.1). This provides the conceptual backbone of the model.

Supplement A3 then links these components to data sources and parameterization logic. Table A3.1 lists the datasets used, while Table A3.2 maps specific model elements to the data and calculations used to quantify them. Tables A3.3a and A3.3b extend this by reporting literature-informed transition values used to inform flow ranges, including overall drinking transitions (Table A3.3a) and pregnancy/postpartum transitions (Table A3.3b).

The structural representation of the model is introduced in the main text of the manuscript (Figures 1-3), which present the alcohol use, pregnancy, and birth control components as simplified views of the integrated system; these figures provide a visual reference that corresponds directly to the stock definitions and data linkages documented here. Together, these sections anchor the model in clearly defined states, triangulated data sources, and literature-informed behavioral flows.

Supplement B1 focuses on model calibration and parameterization for the simulated model. It outlines the estimation approach and reports the full set of parameters used in the model, including prior ranges and units (Supplement B1 Table 1). These parameters cover initial population distributions, transition rates across alcohol use states, transitions into and out of sobriety, postpartum dynamics, and interactions with birth control behavior. This section is where the assumptions embedded in the model are made explicit and traceable.

Supplement B2 reports the results of the estimation process. It provides summary statistics of the posterior parameter distributions (Table B2.1), along with examples of joint posterior behavior (Figure B2.1). This section allows readers to see how the parameter space is updated after fitting, rather than relying only on prior specifications.

Supplement B3 evaluates model fit. It presents posterior predictive checks (Figure B3.1), comparisons between observed data and simulated trajectories (Table B3.1), and standard goodness-of-fit metrics such as MAE, RMSE, and normalized RMSE (Table B3.2). Together with B2, this section shows how well the calibrated model reproduces the empirical patterns used for estimation.

Supplement C and Supplement D provide the simulation outputs underlying the policy analyses. These companion files include the full set of scenario results across variations in program reach, behavioral change group composition, and assumptions related to drinking transitions. Their role is to expose the complete set of model runs rather than only selected results.

Supplement E compiles summary tables and figures for these policy experiments. Supplemental Table 1 reports percent differences in total AEP cases across policy configurations, while Supplemental Table 2 reports corresponding outcomes for risky drinking and AUD-related AEP cases. The accompanying figures (Supplemental Figures E.1-6d) visualize comparisons across baseline and policy scenarios, as well as alternative behavioral assumptions, across different levels of program coverage.

# Supplement A1. Replication Instructions

All model equations and files are available on:

<https://github.com/MJ-LAB-Harvard/alcohol-exposed-pregnancy-simulation>

The 'Vensim Models' folder includes all the materials originally used to develop the model. The calibration and other analysis were done using Vensim DSS version 10.3.2, and researchers can review and explore the graphical user interface for model variables (*Alcohol.vpmx*) using the Vensim PLE, which is the free version. In addition, one can open the '*Alcohol.mdl*' file using a text editor to explore the mathematical equations.

To make the model more accessible, we also provide the standalone Python script (under 'Standalone Python' folder) that translates the ordinary differential equations originally developed in Vensim to a code that can be run in a Python environment using PySD package (Martin-Martinez et al., 2022). One may need to install libraries such as PySD using pip install pysd in a conda or miniforge environment and run the pysdalc.py code. A successful run of the code should generate the plot for NRD[Non,BC], shown in Figure A1.1. As indicated in the final lines of the code, one can change the variable name and use different subscripts to plot other variables in the model. See the documentation of PySD (Martin-Martinez et al., 2022) to utilize the full capabilities of the package and to conduct further analysis on the model.


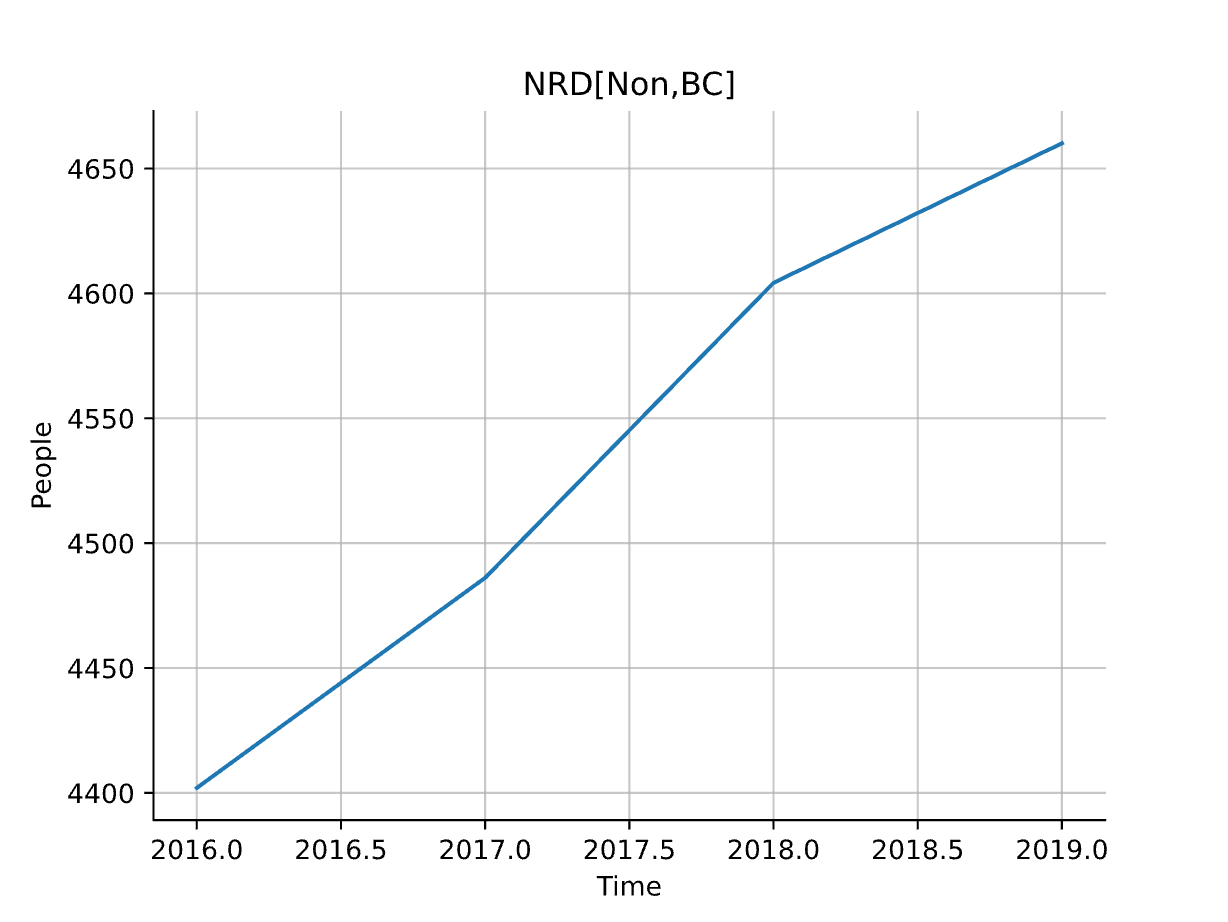


Figure A1.1. Example simulation generated using the standalone Python code

# Supplement A2. Model Structure

**System** dynamics **models represent populations as stocks (state variables) that accumulate over time, and flows (rates) that govern transitions between those states. Each stock changes based on the difference between its inflows and outflows. Formally, for a given stock** $S_{i}(t)$**, where** $S_{i}(t)$ **denotes the number of individuals in the state** $i$ **at time** $t$**:**

$$S_{i}(t)=S_{i}(0)+\int_{0}^{t} (\text{total inflow rate}-\text{total outflow rate})\text{ }d\tau,$$

where $S_{i}(0)$ **is the initial value of that stock,** $\tau$ **denotes simulation time (measured in years), and the inflow and outflow terms represent the total number of individuals moving into and out of that state per unit time. This expression reflects the general structure used throughout the model. Rather than listing each equation separately, all stocks and transitions follow this formulation.**

**The model structure was based on a previously developed system dynamics model, which focused only on alcohol use (Deutsch et al., 2023). This model was grounded in conceptual and empirical alcohol use research related to alcohol use patterns and trajectories over time. The current model builds off of that previously existing model by adding the pregnancy and birth control stocks and flows. Figures for each of these three model constructs can be found in the main manuscript. The full model structure is available for review at:**<https://github.com/MJ-LAB-Harvard/alcohol-exposed-pregnancy-simulation>

**The full set of equations is implemented directly in the Vensim and Python model files, allowing readers to inspect and reproduce the model. The operational definitions of stocks and flows are provided in Supplement A2 Tables 1 and 2, while** the **corresponding transition processes and parameter values are described in Supplements A3 and B.**

It should be noted that feedback loops in this model involve movement through the stock and flow structure (e.g., an increase in the number of people moving from AUD to abstinence may result in an increase in the number of people subsequently relapsing in the next year, holding the percent of relapse constant). All auxiliary variables in the model relate to specific flows (e.g., fraction of people moving from stock 1 to stock 2, or average time until moving from stock 1 to stock). One purpose of this model was to create a structure in which additional system components can be added. For example, considering the loop between AUD abstinence and relapse, the existing model could be further revised to include additional auxiliary variables that account for the role of a “sober community” in sustaining abstinence and reducing relapse rates.

**Population groups in this study are represented as stocks, in which each stock is defined by (1) pregnancy status, (2) alcohol use state, and (3) birth control use.** For example, risky drinkers who are not pregnant and use birth control would be defined in the model as RD[Non, BC].

Flows are structured in such a way that non-pregnant people are able to move between alcohol statuses only (same birth control state), birth control statuses only (same alcohol status), or both. All nonpregnant stocks (all alcohol use states and all birth control use states) also include flows for aging in and out (turning 15 or 45), moving in or out (of the community), and mortality. Each nonpregnant stock also has a flow to the pregnancy stock of the same corresponding alcohol and birth control state.

To account for alcohol use changes during pregnancy, flows are also included from drinking to non-drinking stocks (non-drinking to drinking stocks not included given negligible). We accounted for people who may change behaviors immediately at the beginning of pregnancy (stopping drinking after they learn of their pregnancy), and those who may have delayed a change. We did not include flows that would represent changes from no drinking to drinking over pregnancy, given negligible percentages of this transition in the literature (e.g., Bandoli et al., 2022; Dukes et al., 2017; Fortin et al., 2016; Liu, Mumford & Petras, 2015)

People shifting from non-pregnancy to pregnancy stocks also remained in their birth control use stock until the end of their pregnancy, regardless of alcohol use change during pregnancy, but could change birth control use behavior during a shift from a pregnant to a nonpregnant stock (going on birth control is common after giving birth). Pregnancy stocks also included a miscarriage flow (~15%, divided into 12% first, 2% second, and 1% third trimester). For parsimony (given very low numbers) we did not include aging, mobility, or mortality flows for pregnancy stocks.

Supplement A2 Table 1: Operational definitions for model stocks.

| Stock Status (Model Variable Label) | | Operational Definition |
| --- | --- | --- |
| Pregnancy status | Pregnant (Preg) | Women, aged 15-44, who are pregnant |
|  | Non-Pregnant (Non) | Women, aged 15-44, who are not pregnant |
| Alcohol Use  (one stock for each pregnancy status) | Never-Drinker (ND) | Women aged 15-44 who have never had a drink of alcohol in their lifetime |
|  | Non-Risky Drinker (NRD) | Past-year women aged 15-44 drinkers who have had a drink in the past year, but who have not had past-month binge drinking, past-year AUD, or are in treatment |
|  | Risky Drinker (RD) | Past-month women aged 15-44 binge drinkers ($\geq$4 drinks in one drinking session) who do not have past-year AUD |
|  | Alcohol Use Disorder (AUD) | For NSDUH data: Past-year women aged 15-44 who meet thresholds for DSM 4 alcohol use disorder abuse and/or dependence, who are not currently abstaining or are in treatment.  *Note: some data sources, (BRFFS), and some literature (see below) did not assess AUD or AUD in this way, proxy variables to distinguish non-disordered and disordered risky drinking utilized heavy drinking and binge drinking combined. Heavy drinking is operationalized as drinking 8 or more standard drinks per week* |
|  | Abstainer^⭘^ [NRD, RD, AUD] | Women aged 15-44 who areever-drinkers, but not past-year drinkers. Drinking behavior is specified as the last drinking stock that the person was in before transitioning to abstention |
|  | (AUD) Treatment | Women aged 15-44 ever-drinkers who are in outpatient or inpatient treatment (not detoxification) for AUD |
| Birth Control Use (Sub-stock – one for all pregnant and non-pregnant alcohol stocks | Non- Birth Control User (NBC) | Non-pregnant sexually active◆ women aged 15-44 who report not using contraception the last time they had heterosexual intercourse  *(Note: Birth control use status is maintained in transitions to pregnancy until transition to non-pregnant stock)* |
|  | Birth Control User (BC) | Non-pregnant sexually active◆ women aged 15-44 who report using contraception the last time they had heterosexual intercourse, OR women who report not being sexually active/not having heterosexual intercourse◆  *(Note: Birth control use status is maintained in transitions to pregnancy until transition to non-pregnant stock)* |

^⭘^ Abstainer is equivalent to the “Sober” variable in Python and Vensim model syntax.

◆ sexually active is operationalized as reporting ever/recent penile-vaginal sexual activity

Supplement A2 Table 2: Operational Definitions for Model Flows

| Flow | Inflow Stock | Outflow Stock | Operational Definition |  |
| --- | --- | --- | --- | --- |
| Population Dynamic Flows | | | | |
| People Aging in  (all Non pregnant, for both BC/NBC substocks) | ND Non | NA | Non-pregnant Never-drinkers turning 15 |  |
|  | NRD Non | NA | Non-pregnant non-risky drinkers turning 15 |  |
|  | RD Non | NA | Non-pregnant risky drinkers turning 15 |  |
| People Moving in (all Non pregnant, for both BC/NBC substocks) | ND Non | NA | Non-pregnant never drinkers moving into the community |  |
|  | NRD Non | NA | Non-pregnant non-risky drinkers moving into the community |  |
|  | RD Non | NA | Non-pregnant risky drinkers moving into the community |  |
|  | AUD Non | NA | Non-pregnant people with AUD moving into the community |  |
|  | Abs. NRD Non | NA | Non-pregnant abstaining non-risky drinkers drinkers moving into the community |  |
|  | Abs. RD Non | NA | Non-pregnant abstaining risky drinkers moving into the community |  |
|  | Abs. AUD Non | NA | Non-pregnant abstaining people with former AUD moving into the community |  |
| People Aging Out  (all Non pregnant, for both BC/NBC substocks) | ND Non | NA | Non-pregnant never drinkers turning 45 |  |
|  | NRD Non | NA | Non-pregnant non-risky drinkers turning 45 |  |
|  | RD Non |  | Non-pregnant risky drinkers turning 45 |  |
|  | AUD Non | NA | Non-pregnant people with AUD turning 45 |  |
|  | Abs. NRD Non |  | Non-pregnant abstaining non-risky drinkers drinkers turning 45 |  |
|  | Abs. RD Non | NA | Non-pregnant abstaining risky drinkers turning 45 |  |
|  | Abs. AUD Non | NA | Non-pregnant abstaining people with former AUD turning 45 |  |
| People Moving Out (all Non pregnant, for both BC/NBC substocks) | NA | ND Non | Nonpregnant never-drinkers moving out of the community |  |
|  | NA | NRD Non | Nonpregnant non-risky drinkers moving out of the community |  |
|  | NA | RD Non | Nonpregnant risky-drinkers moving out of the community |  |
|  | NA | AUD Non | Nonpregnant people with AUD moving out of the community |  |
|  | NA | Abs. NRD Non | Nonpregnant abstaining non-risky drinkers moving out of the community |  |
|  | NA | Abs. RD Non | Nonpregnant abstaining risky-drinkers moving out of the community |  |
|  | NA | Abs. AUD Non | Nonpregnant abstaining people with former AUD moving out of the community |  |
| Mortality  (all Non pregnant, for both BC/NBC substocks) | NA | ND Non | Nonpregnant never-drinkers mortality |  |
|  | NA | NRD Non | Nonpregnant non-risky drinkers mortality |  |
|  | NA | RD Non | Nonpregnant risky-drinkers mortality |  |
|  | NA | AUD Non | Nonpregnant people with AUD mortality |  |
|  | NA | Abs. NRD Non | Nonpregnant abstaining non-risky drinkers mortality |  |
|  | NA | Abs. RD Non | Nonpregnant abstaining risky-drinkers mortality |  |
|  | NA | Abs. AUD Non | Nonpregnant abstaining people with former AUD mortality |  |
| Alcohol Use Flows For Non-Pregnant People (ignoring Birth Control Use switches) | | | | |
| Alcohol Use Onset (Non pregnant, for both BC/NBC Substocks) | NRD Non | ND Non | Nonpregnant people starting to drink (non-risky) |  |
|  | RD Non | ND Non | Nonpregnant people starting to drink (risky) |  |
| NRD Non outflows (Non pregnant, for both BC/NBC Substocks) | RD Non | NRD Non | Nonpregnant Non-risky drinkers starting to risk drink |  |
|  | Abs. NRD Non | NRD Non | Nonpregnant Non-risky drinkers starting to abstain |  |
| RD Non outflows (Non pregnant, for both BC/NBC Substocks) | AUD Non | RD Non | Nonpregnant Risky drinkers with onset of AUD |  |
|  | Abs. RD Non | RD Non | Nonpregnant Risky drinkers starting to abstain |  |
|  | NRD Non | RD Non | Nonpregnant Risky drinkers transitioning to non-risky drinking |  |
| AUD Non outflows (Non pregnant, for both BC/NBC Substocks) | AUDtx Non | AUD Non | Nonpregnant People with AUD going into treatment |  |
|  | Abs. AUD Non | AUD Non | Nonpregnant People with AUD becoming abstinent |  |
|  | RD Non | AUD Non | Nonpregnant People with AUD transitioning to risky drinking (no AUD) |  |
|  | NRD Non | AUD Non | Nonpregnant People with AUD transitioning to non-risky drinking |  |
| AUD Treatment outflows (Non pregnant, for both BC/NBC Substocks) | AUD Non | AUDTx Non | Nonpregnant People who did not complete AUD treatment |  |
|  | NRD Non | AUDTx Non | Nonpregnant people who completed AUD treatment and transitioned to NRD |  |
|  | RD Non | AUDTx Non | Nonpregnant people who completed AUD treatment and transitioned to RD |  |
|  | Abs. AUD Non | AUDTx Non | Nonpregnant people who completed AUD treatment and transitioned to Abs. AUD |  |
| Abstainer to Drinker Outflows (Non pregnant, for both BC/NBC Substocks) | NRD Non | Abs. NRD Non | Current abstainers (former non-risky drinkers) starting non-risky drinking ◊ |  |
|  | RD Non | Abs. RD Non | Current abstainers (former nrisky drinkers) starting risky drinking ◊ |  |
|  | AUD Non | Abs. AUD Non | Current abstainers (former people with AUD) relapsing to AUD ◊ |  |
| Birth Control Use Flows for Non-Pregnant People | | | | |
| Birth Control Outflows (Non Pregnant) | BC Non (Same Alcohol Stock) | NBC Non (Same Alcohol Stock) | Nonpregnant people stoping their birth control use, but not changing their alcohol use |  |
|  | BC Non (Alcohol Stock 1) | NBC Non (Alcohol Stock 2) | Nonpregnant people stoping their birth control use, and also changing their alcohol use |  |
| Non Birth Control Outflows (Non Pregnant) | NBC Non (Same Alcohol Stock) | BC Non (Same Alcohol Stock) | Nonpregnant people starting to use birth control, but not changing their alcohol use |  |
|  | NBC Non (Alcohol Stock 1) | BC Non (Alcohol Stock 2) | Nonpregnant people starting to use birth control use, and also changing their alcohol use |  |
| Pregnancy-Related Flows | | | | |
| Transition To pregnancy (Birth control use stock stays the same) | ND Preg | ND Non | Never drinkers becoming pregnant |  |
|  | NRD Preg | NRD Non | Non-Risky drinkers becoming pregnant |  |
|  | RD Preg | RD Non | Risky drinkers becoming pregnant |  |
|  | AUD Preg | AUD Non | People with AUD becoming pregnant |  |
|  | Abs. NRD Preg | Abs. NRD Non | Abstaining non-risky drinkers becoming pregnant |  |
|  | Abs. RD Preg | Abs. RD Non | Abstaining risky drinkers becoming pregnant |  |
|  | Abs. AUD Preg | Abs. AUD Non | Abstaining people with former AUD becoming pregnant |  |
|  | AUD Tx Preg | AUD Tx Non | People in treatment for AUD becoming pregnant |  |
| Changing alcohol use during pregnancy (Reduction /Abstention) | Abs NRD Preg | NRD Preg | Non-risky drinkers who became pregnant and started abstaining  *Includes both immediate transition and delay |  |
|  | Abs. RD Preg | RD Preg | Risky drinkers who became pregnant and started abstaining  *Includes both immediate transition and delay |  |
|  | NRD Preg | RD Preg | Risky drinkers who became pregnant and started reducing their drinking to non-risky levels  *Includes both immediate transition and delay |  |
|  | Abs. AUD Preg | AUD Preg | *Note, given that AUD diagnosis requires past-year symptoms, people with AUD who reduce drinking, but do not become abstinent, are still considered to be in the “AUD” stock, as pregnancy lasts less than 1 year, but we still wanted to distinguish between drinking and abstinence here. |  |
| Treatment Utilization during pregnancy | AUD Preg | AUD Tx Preg | Pregnant people with AUD who initiate treatment during pregnancy |  |
|  | AUD Tx Preg | AUD Preg | Pregnant People who do not complete AUD treatment during pregnancy |  |
|  | AUD Tx Preg | Abs AUD Preg | Pregnant People who complete AUD treatment during pregnancy |  |
| Miscarriage | ND Non | ND Preg | Pregnant never drinkers who miscarry |  |
|  | NRD Non | NRD Preg | Pregnant non-risky drinkers who miscarry |  |
|  | RD Non | RD Preg | Pregnant risky drinkers who miscarry |  |
|  | AUD Non | AUD Preg | Pregnant people with AUD who miscarry |  |
|  | Abs. NRD Non | Abs. NRD Preg | Pregnant abstaining non-risky drinkers who miscarry |  |
|  | Abs. RD Non | Abs. RD Preg | Pregnant abstaining risky drinkers who miscarry |  |
|  | Abs. AUD Non | Abs. AUD Preg | Pregnant abstaining people with former AUD who miscarry |  |
|  | AUD Tx Non | AUD Tx Preg | Pregnant people in treatment for AUD who miscarry |  |
| Giving Birth | ND Non | ND Preg | Pregnant never drinkers who give birth |  |
|  | NRD Non | NRD Preg | Pregnant non-risky drinkers who give birth |  |
|  | RD Non | RD Preg | Pregnant risky drinkers who give birth |  |
|  | AUD Non | AUD Preg | Pregnant people with AUD who give birth |  |
|  | Abs. NRD Non | Abs. NRD Preg | Pregnant abstaining non-risky drinkers who give birth |  |
|  | Abs. RD Non | Abs. RD Preg | Pregnant abstaining risky drinkers who give birth |  |
|  | Abs. AUD Non | Abs. AUD Preg | Pregnant abstaining people with former AUD who give birth |  |
|  | AUD Tx Non | AUD Tx Preg | Pregnant people in treatment for AUD who give birth |  |
| Birth Control Use Transition after birth | NBC Preg (Same Alcohol Stock) | BC Non (Same Alcohol Stock) | People giving birth who did not previously use birth control going on birth control after pregnancy |  |

Note: all references to people relate to women between the ages 15-44.

All flow time units are based on 1 year.

◊ Considers the additional proportion of people who became abstinent during pregnancy and gave birth while in an abstaining stock, who may be more likely to return to drinking – see Supplement A3 Table 5

# Supplement A3. Model parameterization

As discussed in the manuscript, we used three main strategies to parameterize model constructs; information from datasets (raw data, crosstabulations, or annual reports), information from research/literature, and calibration. Below, we detail the data and literature used, the strategies we used for their parameterization, and the stocks and flows that were informed through data and literature.

## Dataset-Informed Parameterization

We used publicly available datasets to inform parameterization for all model stocks (and some flows). Given that we wanted our model to represent the study site as closely as possible, we focused on data that could provide us with local, regional, or state parameters as much as possible. This often required considering multiple sources of data, as there is no single dataset that includes pregnant and non-pregnant people that simultaneously includes a detailed assessment of alcohol use (including never use, past year and previous year use, binge drinking, alcohol use disorder, and alcohol use treatment), alongside recent heterosexual activity, current contraception use, and pre-pregnancy/post-pregnancy contraception use.

Supplement A3 Table 1 provides a list of all datasets, formats (online data dashboards and analysis tools, raw data, and annual reports), specific variables used, and how they were used to parameterize the model. Except for census data, which provided us with population frequencies, the datasets listed in this table were used to find the percent of pregnant and non-pregnant women aged 15/18-44 engaged in specific behaviors, often through cross-tabulating different behaviors. For example, NSDUH could be used to examine the percentage of non-pregnant women aged 15-44, from our study state, who reported past-month binge drinking, and did not meet criteria for past-year AUD.

Dataset-informed parametrization involved triangulating data by applying percentages of behaviors to the number of people within our target population. Supplement A3 Table 2 provides the overall strategies we used for triangulating data to parameterize specific stocks and flows. We used the base census data to attain the number of pregnant and non-pregnant people in the population, and calculated the number of people in each alcohol stock by multiplying the total pregnant and non-pregnant populations with the percentages gained through data cross-tabulations or annual report (PRAMS) numbers. Finally, we calculated the number of people in each birth control specific alcohol stage stock by multiplying the number of people in each pregnant and non-pregnant alcohol stage stock with the percentage of people who use or do not use birth control within each drinking stage and by pregnancy. For example, we discerned the number of people in the non-pregnant, risky drinking, non-birth control use stock through multiplying the population of non-pregnant people in a given year (American Community Survey) by the percent of non-pregnant people who reported past-month binge drinking (NSDUH) in that year, and the percent of past-month binge drinkers who reported engaging in heterosexual activity without using contraception in that given year (BRFSS, YBRFSS).

Supplement A3 Table 1. Datasets and dataset variables used for model parametrization

| Dataset/ Years | Data Type / Analysis | Data Details | Parameterization Use |
| --- | --- | --- | --- |
| 5-year American Community Survey (ACS), 2015 – 2019 | Data Type: Online census dashboard  Data analysis: Exported raw data, which was frequencies and percentages from dashboard output. | Variables: Demographics for fertility (number of women who gave birth, by age), and mobility (number of women and number of people aged 15-44 moving in and out)  Inclusion criteria: women aged 15-44 living within the study area. | Base population numbers of pregnant/non-pregnant w15-44 for clear numbers approximating the number of pregnant/non-pregnant people in each stock and mobility flows |
| National Survey on Drug Use and Health, (NSDUH) 2015-2016 through 2018-2019 | Restricted Data: (state-specific)  Data Type: online data analysis system from SAMHSA  Data Analysis: output from created cross-tabulations  Publicly Available Data: (national)  Data type: Raw data (publicly available online to download)  Data Analysis: weighted cross-tabulations via SAS | Variables: alcohol use (ever used, initiation, past year drinking, past-month drinking, monthly binge drinking, AUD, previous year use, and alcohol use treatment, detailed age group, pregnancy status, trimester of pregnancy, month of pregnancy  Inclusion criteria: women aged 15-44, and (when examining restricted data) the community state and 3 neighboring states in the Northern Plains/Mountain area | Percentages informed alcohol –related stocks and flows for pregnant and non-pregnant stocks |
| Treatment Episode Dataset- Discharges, (TEDS) 2016-2019 | Data type: raw data (publicly available online to download), Data analysis: cross-tabulations via SAS | Variables: inpatient/outpatient treatment for alcohol use/alcoholism, treatment completion proportions, alcohol use behavior at discharge, length of stay time, pregnancy status  Inclusion criteria: women aged 15-44 from state and core-based statistical area of the study site | Percentages informed treatment-related model components, including specific number of pregnant and non-pregnant people in treatment and treatment outflows |
| Behavior Risk Factor Surveillance System, (BRFSS) 2017; 2019 | Data type: Raw data from core dataset and family planning module (publicly available to download)  Data Analysis: weighted cross-tabulations via SAS  Note: as years 2016 and 2018 were missing, we calculated these values as:  yr2018 value = (yr2017 value + yr2019 value)/2  yr2016 value = yr2017 value + (yr2017 value – yr2018 value) | Variables: heterosexual sexual activity and contraception use, past-month drinking (none/any, binge, and heavy drinking (AUD proxy), detailed age group. Note that there is no “ever drinking” question, (lumping never drinking/current abstention together) so discerning ND, abstaining NRD, abstaining RD, and abstaining AUD categories for triangulating NSDUH and BRFSS data were done based on age ranges of affiliated alcohol use stocks from NSDUH data.  Inclusion Criteria: non-pregnant women aged 18-44 from study site and states with comparable demographics | Percentages informed proportions of BC/ NBC per alcohol use behavior for non-pregnant stocks |
| Youth Behavior Risk Factor Surveillance System, (YBRFSS) 2015, 2017, 2019 | Data type: online data analysis tool  Data analysis: Cross tabulations | Variables: heterosexual sexual activity, contraception use, any alcohol consumption, binge drinking (when applicable), grade level  Inclusion Criteria: women in grades 10-12 (proxy for ages 15-18) from the study site state | Percentages informed proportions of birth control/ non-birth control use per alcohol use behavior for non-pregnant stocks |
| Pregnancy Risk Assessment Monitoring System (PRAMS), 2016 - 2019 | Data type: annual reports of study site state.  Data analysis: none. | Variables used: percent of new mothers reporting alcohol use 3 months before pregnancy, who drank alcohol during pregnancy, and who started birth control after giving birth | Percentages informed inflows to pregnant stocks by alcohol use and birth control use, informing post-pregnancy birth control outflows |
| CDC WONDER (2016-2019) | Data type: online data analysis system  Analysis: crosstablulations | Variables Included: number of deaths per year, % of deaths that were w15-44, % of deaths that were alcohol-induced causes  Inclusion Criteria: State of study site | Percentages informed mortality flows for stocks. |

Supplement A3 Table 2 Variables and triangulation calculations used for data-based parameterization

| Category | Variable/ Model component | | Data Source | | Variable | |
| --- | --- | --- | --- | --- | --- | --- |
| Pregnancy status stocks | Pregnant (Preg) | | ACS | | # women aged 15-44 who gave birth $\times$ 1.15 (adding the average miscarriage rate) | |
|  | Non-Pregnant (Non) | | ACS | | # women aged 15-44 in the target community – pregnant women | |
| Alcohol Use Stocks | Never-Drinker (ND) | [Non] | NSDUH | % of nonpregnant w15-44 reporting ever having a drink x # nonpregnant w15-44 | |  |
|  |  | [Preg] | NSDUH | % of pregnant w15-44 reporting ever having a drink | |  |
|  | Non-Risky Drinker  NRD | [Non] | NSDUH | % of the calculated # of w15-44 of past year drinkers - RD, AUD, and treatment (non-pregnant) | |  |
|  |  | [Preg] | NSDUH | % of the calculated # of w15-44 of past *month* drinkers subtracting RD, AUD, and treatment (by trimester when possible)■ | |  |
|  |  |  | PRAMS | % of women reporting drinking 3 months before pregnancy, - women reporting binge drinking 3 months before pregnancy | |  |
|  | Risky Drinker  (RD) | [Non] | NSDUH | % non-pregnant w15-44 who are past-month binge drinkers that do not report AUD | |  |
|  |  | [Preg] | NSDUH | % pregnant w15-44 who are past-month binge drinkers that do not report AUD (by trimester when possible)■ | |  |
|  |  |  | PRAMS | % of women reporting binge drinking 3 months before pregnancy | |  |
|  | Alcohol Use Disorder (AUD) | [Non] | NSDUH | % non-pregnant w15-44 who meet DSM 4 classification of either abuse or dependence (non-pregnant) | |  |
|  |  | [Preg] | NSDUH | % pregnant w15-44 who meet DSM 4 classification of either abuse or dependence (by trimester when possible)■ | |  |
|  | Abstaining [NRD, RD, AUD] | [Non] | NSDUH | % non-pregnant w15-44 Ever-drinkers who did not report past-year drinking, based on estimated proportions of people who will become sober (start abstaining) from their specific drinking category out of all people who become abstinent | |  |
|  |  | [Preg] | NSDUH | % pregnant w15-44 Ever-drinkers who did not report past *month* drinking, based on estimated proportions of people who will become sober (start abstaining) from their specific drinking category out of all people who become abstinent (pregnant, by trimester when possible to consider pregnancy rates for abstaining stocks and “final” stock states based on pregnant people transitioning to abstention over pregnancy) | |  |
|  |  |  | PRAMS | % of women reporting not drinking 3 months before pregnancy out of % of women reporting ever drinking | |  |
|  | AUD Treatment  (AUDTx) | [Non] | NSDUH | % non-pregnant w15-44 who report having alcohol use disorder and also report current treatment $\times$ % non-pregnant | |  |
|  |  |  | TEDS | % non-pregnant w15-44 in inpatient and outpatient treatment for alcohol use $\times$ % non-pregnant | |  |
|  |  | [Preg] | NSDUH | % pregnant w15-44 who report having alcohol use disorder and also report current treatment | |  |
|  |  |  | TEDS | % pregnant w15-44 in inpatient and outpatient treatment for alcohol use | |  |
| Alcohol Use flows | Alcohol Use Onset  ND[Non]→ NRD[Non]  ND[Non] → RD[Non] | | NSDUH | | % w15-44 initiating drinking in past year, cross tabulated with binge drinking status (non-pregnant only) | |
| Alcohol use Flows to Abstinence (Pregnant) | AUD/RD/NRD [Preg] - > Abs AUD/RD/NRD [Preg]  Total Percent of flow | | NSDUH  (required national data from restricted dataset): | | % of pregnant people in the first month of pregnancy who report past-month drinking (NRD: non-binge drinking only, no AUD; RD = binge drinking, no AUD; AUD = any drinking and AUD) - % of pregnant people in the last trimester of pregnancy reporting NRD, RD, and AUD | |
|  |  |  | PRAMS | | % of women who report drinking 3 months before pregnancy (assume in drinking stock when pregnant) - % of women who report drinking in the last 3 months of pregnancy | |
|  | AUD/RD/NRD [Preg] - > Abs AUD/RD/NRD [Preg]  Timing of flow | | NSDUH (required national data from restricted dataset) | | “Immediate change”: % of pregnant people in the first month of pregnancy who report past-month drinking (NRD: non-binge drinking only, no AUD; RD = binge drinking, no AUD; AUD = any drinking and AUD) - % of pregnant people who report past-month drinking in months 2-3.  “Delay change”: % of pregnant people in first month of pregnancy who report past-month drinking (NRD: non-binge drinking only, no AUD; RD = binge drinking, no AUD; AUD = any drinking and AUD) - % of pregnant people who report past-month drinking in the second trimester (months 4-6) - % of pregnant people who report past-month drinking in months 2-3 | |
| Birth Control Use Sub-Stocks | Non-Birth Control Use (NBC) | [Non] | BRFSS (18-44 year olds) | % sexually active◆ women aged 18-44, cross-tabulated by contraception use and drinking status | |  |
|  |  |  | YRBSS (15-18 year olds) | % sexually active◆ 10th, 11th, and 12th grade women, cross-tabulated by contraception use and drinking status | |  |
|  |  | [Preg] | PRAMS | % of women who reported not using birth control prior to pregnancy | |  |
|  | Birth Control Use (BC) | [Non] | BRFSS (18-44 year olds) | % sexually active non-pregnant women, aged 18-44, cross-tabulated by contraception use and drinking status | |  |
|  |  |  | YRBSS (15-18 year olds) | % sexually active 10th, 11th, and 12th grade (e.g., 15-17/18) women cross-tabulated by contraception and drinking status | |  |
|  |  | [Preg] | PRAMS | % of women who reported using birth control prior to pregnancy | |  |
| Birth Control Use Flows | Preg NBC → Nonpreg BC | | PRAMS | | % of women who report starting birth control after giving birth | |
| Population Dynamics Flows | Aging In flows (ND, NRD, and RD, BC and NBC; AUD not included due to low occurrence, Abstaining stocks not included for parsimony (all 15 year olds reporting no drinking in past year were considered never drinkers) [Non] | | # women turning 15 (based on # women aged 14 from the previous year; [ e.g., # 10-14 female population $\div$ 5]) in the target community (ACS) multiplied by:  ND (BC; NBC): proportion of 9^th^ grade women reporting not currently drinking (assume ND if not current drinking) crosstabulated by sexual activity and contraception use (YRBSS)  NRD (BC; NBC): proportion of 9th-grade women reporting current, but not binge drinking, crosstabulated by sexual activity and contraception use (YRBSS)  RD (BC; NBC): proportion of 9^th^-grade women reporting current binge drinking, crosstabulated by sexual activity and contraception use (YRBSS) | | | |
|  | Aging out flows (all [Non] stocks) | | # of women turning 45 (based on # of women aged 44 from previous year, e.g., # 40-44 female population $\div$ 5]) in the target community (ACS), multiplied by:  % of women aged 40-44 in ND, NRD, RD, AUD, and Abstaining (all) categories (NSDUH)  % of women aged 40-44 in each drinking category who are sexually active and using birth control or who are not sexually active (BC) or who are sexually active and not using birth control (NBC) (BRFSS) | | | |
|  | Nonpregnant only; moving in/out flows (all [Non] stocks) | | # of w15-44 moving in and out of target community (ACS) by detailed age (15-17; 18-24; 25-29; 30-34;35-39; 40-44)  Assigned specific numbers based on ACS information by multiplying these numbers by the proportion of w15-44 in the existing population in corresponding age groups at each stock (calculated thorugh NSDUH, BRFSS, and YRBSS age, alcohol use, and birth control use crosstablulations), and the proportion of people in each stock as a percent of the entire w15-44 population. | | | |
|  | Nonpregnant only; Mortality flows (all [Non] stocks) | | % of deaths that were w15-44 CDC WONDER Mortality (State Data). RD and AUD mortality flows were calculated by examining the % of w15-44 deaths that were attributable to alcohol-induced causes, and all other stock mortality flows were calculated by subtracting alcohol-induced cause deaths from all deaths | | | |

◆ Sexually active is operationalized as reporting ever/recent penile-vaginal sexual activity

■ NSDUH restricted data that is accessible online will not provide cross-tabulations that include cell proportions that approach 0%. Some cross-tabulations, especially those that involved examining trimesters, were unable to be calculated, or required more variables than would be allowed in the cross-tabulation. The trimester variable is not available for the public access dataset.

## Literature-Informed Parameterization

We also drew from alcohol use literature that could help us inform alcohol related flows (both pregnant and non-pregnant flows) that were otherwise difficult or unable to be attained based on cross-sectional data. We used longitudinal studies that could provide information on changes or patterns of behavior over time, especially studies that provided means, frequencies, or percentages of behaviors that were assessed in discrete stages of alcohol use.

This information was used to inform a potential range for the flow estimates, rather than considering discrete numbers that could be calculated more precisely for stocks. Tables 3 (nonpregnant drinking) and 4 (Postpartum abstainer to drinker shifts) provide values we utilized from studies used to inform alcohol use flows for pregnant and non-pregnant stocks, including specific pages, tables, or figures in which we located values.

Although there were times that we were able to use the specific values stated within the articles listed below, there were also times in which we attained values through additional calculations using article findings. For example, Koenig et al., 2020, is a longitudinal study that reports transitions of alcohol use stages over time, and includes a table that provides numbers and percentages for the frequencies of the sample transitioning to consecutive alcohol use stages. To attain the percent of people who transition to risky drinking (as well as the percent of non-risky drinkers that may never transition to risky drinking), we could use the values reported for the number of people who ever engage in heavy (binge not specified) drinking divided by the number of people who reported ever taking a drink. We could then divide this attained value (75%, as seen in Table 3) and divide it by the average time specified for transitioning from non-risky drinking to risky drinking in this same manuscript (average 5.3 years), to attain an approximate number for the rate of (never risky) non-risky drinkers who would transition (first onset) to risky drinking per year. Tables 3 and 4 provide the article-based calculations used to derive specific values for literature-informed parameterization.

Supplement 3A Table 4 Literature-Informed Transitions for Flow Ranges – Overall Nonpregnant Drinking

| Flow | Parameter Variable | Value | Reference | Calculations used to derive specific literature-based parameters |
| --- | --- | --- | --- | --- |
| NRD → Abs. NRD (From AUD Remission) | Rate of AUD who are NRD at treatment discharge, becoming Abs. | 0.22 | Kline‐Simon et al., 2013, Tables 2-3, Results Text | Calculations NA: Text |
|  |  |  |  | *Note: Using “Bivariate analysis of drinking status and ASI severity at 12 months" results. Consider 6 month status as "discharge" of those in state "x" at 6 months, the % moving to state Y at 1 yr* |
|  | % of NRD who are former AUD (treated) | 0.1 | Hingson et al., 2017, Append. Table. 1; Fan et al., 2019 Table. 3 | # people reporting only NRD in the past year (Hingson) $\div$ # people with former AUD who received treatment. and report past-year NRD (Fan) |
|  |  |  |  | *Note: Higson and Fan use NESARC III, allowing for comparisons if groups are from the same sample* |
| NRD →RD (Onset) | Rate of never-RD NRD/or ND moving to RD | 0.07 | Lee et al., 2018, Table. 2 | 21% people changing from risky drinking to Abs. or low risk drinking$\div$ 3 yrs |
|  |  |  |  | *Note: Combine probabilities for Wave 1-2 stage transitions weighted by proportion in each age group,* $\div$*by avg. time for follow-up (2 to 4 yrs between waves, so 3)* |
|  | % of NRD ever moving to RD | 0.75 | Koenig et al., 2020 Table. 1 | 1295 people initiating heavy drinking $\div$1738 people taking their first drink |
|  |  |  |  | *Note: All calculations for % of stock changing stages are calculated as # people reaching next drinking stage* $\div$*by # people reaching previous stage* |
|  | Rate of never-RD moving to RD | 0.14 |  | 75 % people initiating heavy drinking $\div$ avg 5.3 yrs between first drink and heavy drinking |
|  |  |  |  | *Note: All calculations for rates between stocks are calculated as % people attaining next stage,* $\div$*by the avg yrs between stages* |
|  | Rate of treated AUD who are NRD at discharge moving to RD | 0.31 | Kline‐Simon et al., 2013, Table.2-3, Results Text | NA - text |
| RD → Abs. RD/NRD (Remission) | % of RD moving from RD | 0.72 | Koenig et al., 2020, Table. 1 | 932 people entering remission from heavy drinking $\div$ 1295 heavy drinkers |
|  | Rate of RD moving to NRD or Abs. | 0.06 |  | 72% people in remission from heavy drinking $\div$ 11.5 avg years between heavy drinking and remission |
|  | Rate of RD moving to NRD or Abs. | 0.07 | Lee et al., 2018, Table. 2 | 21% combined probability for people moving from risky drinking to low-risk drinking or Abs. |
| RD → NRD (Remission) | % of NRD who are former RD | 0.09 | Koenig et al., 2020 Table.e 1 | % people who initiate heavy drinking $\times$ % people who remiss from heavy drinking $\times$ % former heavy drinkers who will relapse |
|  | Rate of RD moving to NRD | 0.34 | Palzes et al., 2022, Results Text | 63.1% heavy drinkers ceasing heavy drinking $\times$ 53.1% of cessation as NRD |
| RD →  Abs. RD (Remission) | Rate of RD moving to Abs. RD | 0.3 | Palzes et al., 2022, Results Text | 63.1% heavy drinkers ceasing heavy drinking $\times$ 46.9% of cessation as Abs. |
|  | Rate of AUD who are RD at treatment discharge moving to Abs. RD | 0.2 | Kline‐Simon et al., 2013, Table. 2 | NA - table |
| Former RD [AEDS]→RD | % of former RD [AEDS] moving to RD | 0.17 | Koenig et al., 2020, Table. 1 | 157 relapsing after HD remission $\div$932 people in remission for heavy drinking |
|  | Rate of former RD [AEDS] moving to RD | 0.02 |  | 17% heavy drinkers in remission moving back to heavy drinking $\div$ 9.3 avg years between remission and relapse |
|  | Rate of Abs. RD moving to RD | 0.33 | Palzes et al., 2022 Table. 3 | 2802 people who transitioned from abstinence back to heavy drinking $\div$ 8561 people moving from heavy drinking to Abs. |
| NRD→RD (Return) | Rate of former RD who are NRD, who move to RD | 0.46 | Palzes et al., 2022 Table. 3 | 4859 people who moved from NRD back to heavy drinking $\div$ 10639 people moving from heavy drinking to NRD. |
| RD→AUD (Onset) | % of RD ever moving to AUD | 0.49 | Koenig et al., 2020, Table. 1 | 641 people onset to AUD $\div$1295 heavy drinkers |
|  | Rate of never-AUD moving from RD to AUD | 0.13 |  | 49% heavy drinkers who onset to AUD $\div$ (9.1 avg. years between first drink and AUD - 5.3 avg. years between first drink and heavy drinking) |
|  | Rate of never-AUD moving from RD to AUD | 0.32 | Sartor et al., 2016 Table. 2 | ((55% youth initiating drinking $\times$ 32% of youth attaining AUD) $\div$ (55% youth initiating drinking $\times$ 62% drinkers initiating intoxication)) $\div$ 1.6 avg. years from intoxication to AUD |
|  |  |  |  | *Note: Using onset of intoxication to AUD timing as proxy for RD* |
| AUD → [AEDS] (Remission) | % of AUD moving to less-risky [AEDS] | 0.82 | Koenig et al., 2020, Table 1 | 524 people who in AUD remission $\div$641 people with AUD |
|  | Rate of AUD moving to less risky [AEDS] | 0.07 |  | 82% AUD who recover $\div$ 11.2 avg. years between AUD and recovery |
|  | Rate of AUD moving to Abs. AUD or NRD | 0.39 | Palzes et al., 2020, page 439 | NA - In Text (person adjustment rates) |
|  | Rate of post-treatment AUD moving to Abs. AUD or NRD | 0.351 | Maisto et al., 2018, Results Text, Fig. 2 | 25.5% people in continuous remission + 9.6% people with one transition to remission |
|  |  |  |  | *Note: % of people in latent profile and past-month drinking one year after treatment* |
|  | Rate of AUD moving from AUD [AEDS] | 0.37 | Seeley et al., 2019 pages 47-48 | 89% women recovering from AUD $\div$ 29 avg months from onset to recovery |
|  |  |  |  | *Note: Taken from results section of text – findings for women only* |
|  | Rate of formal treated AUD moving to RD or NRD | 0.08 | Ilgen et al., 2008 Table 2 | 16% people with AUD who were non-problematic drinkers 1 year later $\times$ 50% of non-problematic formal-treated engaged drinkers |
|  |  |  |  | *Note: "problematic" drinking 1 year later proxy for AUD while "non-problematic" includes RD and NRD* |
| AUD → Abs AUD (Remission) | Rate of AUD becoming Abs. (ever treated) | 0.03 | Fan et al., 2019 Table. 3, Pg 2410 | 12% ever treated. people with AUD moving to Abs. AUD $\div$ 4 years |
|  |  |  |  | *Note: Assume "year 0" is 100% AUD. Divide avg % of group (e.g., symptomatic low-risk drinker, abstainer) by 4 years (first year group)* |
|  | Rate of AUD becoming Abs. (never treated) | 0.01 |  | 4% never treated. people with AUD moving to Abs AUD $\div$ 4 years |
|  | Rate of never formal treated AUD moving to Abs. AUD | 0.09 | Ilgen et al., 2008 Table 2 | 36% of AUD who move to Abs. AUD 1 year later $\times$ 32% of Abs. AUD not receiving formal treatment |
|  |  |  |  | *Note: Consider "formal treatment" to mean beyond only participating in AA* |
|  | Rate of AUD moving to Abs. AUD (natural remission) | 0.03 | Tucker et al., 2020, Results Text; Table. 1, Fig. 2 | 61% of pre-resolution AUD (Global high risk + ADS-DPS risk, ~ 38% of 493 sample) in resolved abstinent group $\div$ 17 avg years duration of problem |
|  |  |  |  | *Note: Pre-resolution drinking profiles proxies for NRD, RD, and AUD based on descriptions. Figure 2 required "eyeballing" percentages. Numbers based on follow-up sample. All percentages* $\div$*by avg duration of problem (indicated by resolution group)* |
| AUD →NRD (Remission) | Rate of AUD moving to NRD (natural remission) | 0.01 | Tucker et al., 2020 | 8% of pre-resolution AUD (Global high risk + ADS-DPS risk, ~ 38% of 493 sample) in resolved non-abstinent group $\div$ 16 average years duration of problem |
|  | Rate of AUD moving to NRD (ever treated) | 0.01 | Fan et al., 2019 Table. 3 | 3% + 1% ever treated AUD moving to asymptomatic or symptomatic low risk drinking $\div$4 years |
|  | Rate of AUD moving to NRD (never treated) | 0.02 |  | 5% + 2% never treated. AUD moving to asymptomatic or symptomatic low-risk drinking $\div$4 years |
| AUD →RD (Remission) | Rate of AUD moving to RD (ever treated) | 0.03 | Fan et al., 2019 Table. 3 | 9% + 3% ever treated. AUD moving to asymptomatic or symptomatic low-risk drinking $\div$ 4 years |
|  | Rate of AUD moving to RD (never treated) | 0.07 |  | 12% + 15% never treated. AUD moving to asymptomatic or symptomatic low-risk drinking $\div$ 4 years |
|  | Rate of AUD moving to RD (natural remission) | 0.01 | Tucker et al., 2020 | 25% of pre-resolution AUD (Global high risk + ADS-DPS risk $\times$ ~ 38% of 493 sample) in unstable resolution group $\div$ 17 avg year duration of problem |
| [AEDS] → AUD (Relapse) | % of former AUD who will relapse | 0.13 | Koenig et al., 2020, Table. 1 | 68 people relapsing to AUD $\div$ 524 AUD people who remiss |
|  | Rate of former AUD [AEDS] moving to AUD | 0.01 |  | % of people relapsing to AUD $\div$ 9.1 avg. years to relapse |
|  | Rate of former AUD [AEDS] moving to AUD | 0.07 | Seeley et al., 2019 pages 47-48 | 31.7% of women in AUD recovery who relapse $\div$ 53 avg. months to relapse |
| Abs. AUD →AUD (Relapse) | Rate of Abs. AUD moving to AUD | 0.01 | Dawson et al., 2007, Table 2 | 2% of Abs. AUD who have AUD 3 years later $\div$ 3 years |
| Abs. AUD → NRD | Rate of Abs. AUD moving to NRD | 0.05 | Dawson et al., 2007, Table 2 | 14% of Abs. AUD who are low-risk drinkers 3 years later $\div$ 3 years |
| Abs. AUD. → RD | Rate of Abs. AUD moving to RD | 0.07 | Kline‐Simon et al., 2013, Table. 2-3, results | NA - table |

*Notes: [AEDS]: [Any Ever-Drinker Stock] Any stock that is connected to the inflowing stock, excluding the Never Drinker stock*

*All rate variables are parameterized as annual, e.g., % of people in stock / 1 year.*

*All percentage variables are parameterized as the percent of people within stock(s) over the total number of people in those stock(s)*

Supplement 3A Table 5 Literature-Informed Transitions related to pregnancy and postpartum

| Flow | Parameter Variable | Param. | Reference | Calculations |
| --- | --- | --- | --- | --- |
| Miscarriage Flows | | | | |
| Miscarraige (general) | % of pregnancies ending in miscarriage | .15 | Quenby et al., 2021, text | NA |
|  | % of miscarraiges in first trimester | .8 | ACOG, 2018  Text | NA |
| Miscarriage  (NRD, RD and AUD) | % risk increase for miscarriage (dose response) per drink / week (cap at 5) | .06 | Sundermann et al., 2021  Results text | NA |
| Postpartum Flows for former drinkers (those who changed to Abstinence during pregnancy) | | | | |
| Abs. [AEDS] → any drinking | % Abs. moving to drinking stock | 0.48 | Board et al., 2023, Results Text, Table. 1 | 53% of people reporting drinking postpartum $\times$ 90% who did not report drinking in the past 3 months of pregnancy (approx based on Table 1) |
|  | Time (yrs) for any move from Abs to drinking | 0.79 | Board et al., 2023 | NA - Based on the timing of PRAMS |
|  | % Abs. moving to any drinking stock | 0.43 | Fortin et al., 2016 Fig 1 | NA - based on the figure |
| Abs. [AEDS]→ NRD | % Abs. who will start to drink NRD | 0.47 | Doyle et al., 2023, Table 3, Fig 1, Text Pg 22 | (102 of low-moderate consumers - 49% low-moderate consumers reporting alcohol consumption during pregnancy) + (219 minimal consumers - 19% reporting alcohol consumption during pregnancy) $\times$ 0.4 drink days per week (assuming 0.6 Abs. pop at 12 months) $\div$ 278 total drinkers who do not report drinking during pregnancy |
|  |  |  |  | *Note: "low-moderate consumer class proxy for NRD, and "minimal consumers" proxy for Abs/ NRD. Minimal consumer final number based on 12 months postpartum* |
|  | Time (yrs) for Abs to move to NRD | 0.30 | Doyle et al. 2023 | (Proportion of NRD movers who are from low-moderate group (47%) $\times$ 1 month (see Fig 2) + Proportion of NRD movers who are from minimal consumer group (53%) $\times$ 6 months) $\div$ 12 months |
|  | % of Abs. (any) who move to NRD | 0.31 | Liu et al., 2015, Table. 1 | 33% postpartum women who drink >1 to 3 drinks a week - 3% of women who drink >1 to 3 drinks a week $\div$ 98% of women who abstain last 3 months of pregnancy |
|  |  |  |  | *Note: 1 drink a week proxy for NRD,* $\geq$*4 drinks a week proxy for RD. Consider behavioral continuity of status between pregnancy and postpartum* |
|  | Time (yrs) for Abs. [AEDS] to move to NRD | 0.75 |  | 9 months $\div$ 1 year |
|  | % of Abs. [AEDS] who move to NRD | 0.57 | Jagodzinski and Fleming, 2007 Fig 1, Results Text | % people reporting drinking postpartum - % of risky drinkers postpartum who are either bingers or binge $\div$ heavy |
|  | Time (yrs) for Abs. [AEDS] to move to NRD | 0.25 |  | 3 months $\div$1 year |
|  |  |  |  | *Note: binge drinking only proxy for RD, and binge drinking + heavy drinking proxy for AUD* |
| Abs. [AEDS])/ NRD→ RD | % of Abs. [AEDS] or NRD who move to RD | 0.12 | Tung et al., 2020, Table 3, Results Text | (# people reporting RD 1 year after pregnancy - # people reporting RD during pregnancy) $\div$ (# people sober during pregnancy) |
|  |  |  |  | *Note: Table 3 risky drinking (#* $\geq$*1x a month - #* $\geq$*11 x a week) proxy for % of RD, while* $\geq$*1x a week proxy for RD/AUD Multiply each percentage by the latent group to get numbers of Abs/NRD, RD and RD/AUD. Assume continuity of status between pregnancy and postpartum* |
| Abs. [AEDS] → RD | % of Abs. [AEDS] who move to RD | 0.18 | Jagodzinski and Fleming, 2007 | % risky drinkers drinking postpartum who are binge only |
|  | Time (yrs) for Abs. [AEDS] to move to RD | 0.25 |  | 3 months $\div$ 1 year |
|  | % of Abs. [AEDS] who move to RD | 0.14 | Doyle et al., 2023 | (57 higher consumers - 49% reporting consumption during pregnancy) $\div$278 total drinkers who do not report drinking during pregnancy |
|  |  |  |  | *Note: "higher consumers latent class proxy for RD* |
|  | Time (yrs) for Abs [AEDS] to move to RD | 0.08 |  | Report drinking by first month |
|  | % of Abs. [AEDS] who move to RD | 0.04 | Liu et al., 2015 | 4% women who drink $\geq$4 drinks a week postpartum - 0.1% women who drink $\geq$4 drinks a week during pregnancy $\div$98% of women who abstain during pregnancy |
|  | Time (yrs) for Abs. [AEDS] to move to RD | 0.75 |  | 9 months $\div$1 year |
| Abs. AUD/ NRD  → AUD | % of Abs. AUD who will move to AUD | 0.51 | Forray et al., 2015 Results text | NA- Text |
|  | Time (yrs) for Abs AUD to move to AUD | 0.35 |  | NA - Text |
|  | % of Abs. [AEDS] or NRD who move to AUD | 0.09 | Tung et al., 2020, | (# people reporting RD/AUD 1 year after pregnancy - # people reporting RD/AUD during pregnancy) $\div$ # people sober during pregnancy |
|  | % of Abs. [AEDS] who move to AUD | 0.18 | Jagodzinski and Fleming, 2007 | % of risky drinkers drinking postpartum who are binge /heavy drinkers |
|  | Time (yrs) for Abs. [AEDS] to move to AUD | 0.25 |  | 3 months $\div$ 1 year |
| NA | % of people who are RD at 1 yr postpartum (no transition considered) | 0.08 | Board et al., 2023 | 53% of people reporting drinking postpartum $\times$ 15% who reported this was binge drinking |
|  | % of people who are NRD at 1 yr postpartum (no transition considered) | 0.45 | Board et al., 2023 | 53% of people reporting drinking postpartum $\times$ 85% who did not report binge drinking |

[AEDS]: [Any Ever-Drinker Stock] Any stock that is connected to the inflowing stock, excluding the Never Drinker stock

# Supplement B1. Model calibration

Our system dynamics model is deterministic, meaning that given parameters $\theta$, it produces a single trajectory $x_{it}\left( \theta\right)$, for time series $i$ at different time points $t$. In this setting, calibration is framed as an optimization problem in which model parameters are selected to align simulated trajectories with observed data. The empirical data ($y_{it}$), however, reflect measurement processes (e.g., reporting error, rounding, sampling variation, aggregation across sites) whose exact probability law is unknown and not available in closed form. Because this “true” observation likelihood is intractable and cannot be explicitly written down, we estimate $\theta$ using a Gaussian pseudo log-likelihood, a widely used working approach that focuses on aligning the model’s trajectories with the data while allowing flexible variance scaling (White, 1982, Gourieroux et al., 1984).

We assume heteroskedastic Gaussian working errors in which the observation standard deviation scales proportionally to the simulated values, so that $\mathrm{SD}\left( y_{it} | \theta,\sigma_{i} \right)=\sigma_{i}x_{it}(\theta)$. Conditional on $\theta$ and $\sigma$, we use working independence assumption across observed series and observation times. Thus, the pseudo-likelihood is the product of individual Gaussian factors, yielding the pseudo log-likelihood:

$$\mathcal{l}\left( \theta, \sigma\right)=-\frac{1}{2}\sum_{i} \sum_{t} \left[ \frac{\left( y_{it}-x_{it}\left( \theta\right) \right)^{2}}{\sigma_{i}^{2}x_{it}^{2}\left( \theta\right)}+2ln (\sigma_{i}x_{it}(\theta)) \right],$$

where $\sigma_{i}>0$ and $x_{it}\left( \theta\right)>0$ for all fitted observations, and $i$ indexes the 32 observed time series and $t$ indexes observation times within each series.

The objective is to maximize the pseudo log-likelihood function given the model specifications and target data, which includes 32 time series corresponding to each alcohol drinking stage, pregnancy, and birth control use behavior. All time series are treated simultaneously within a single likelihood function, so that trade-offs across model components are resolved within a unified estimation framework.

We estimated 110 model parameters ($\theta$) in addition to 32 series-specific relative error scale parameters ($\sigma$). We assigned bounded uniform priors to each parameter. These priors imposed feasible ranges informed by data, literature, and judgement of the interdisciplinary modeling team, which is made up of researchers with system dynamics expertise and those with alcohol use research expertise. Supplement B1 Table 1 shows the parameters and their prior used during estimation. The ranges listed in the prior column were informed by data and/or literature, in which we assumed a percentage value around the reported number. This was especially important for literature-informed flows, in which we often had multiple potential values from different studies (as seen in Supplement 3A Tables 4 and 5).

The search procedure explores the joint parameter space of all estimated parameters (including initial conditions and transition rates) within their specified bounds. A large number of candidate parameter sets are evaluated during this process, and the resulting likelihood values are used to identify the best-fitting parameter regions. In practice, this involves evaluating a very large number of parameter combinations (on the order of millions of model evaluations) to ensure adequate coverage of the parameter space. The search is continued until further evaluations no longer yield meaningful improvements in the objective function, indicating convergence to stable high-likelihood regions.

The final posterior samples are derived from this search and represent parameter combinations that are consistent with the observed data under the specified likelihood function. In other words, the calibration objective is to jointly optimize all model parameters with respect to their ability to reproduce the observed time series across all modeled outcomes, rather than fitting individual components of the model in isolation. This approach ensures that the model behaves consistently as a system, rather than as a set of separately fitted parts.

Supplement B1. Table 1. Model parameters and their prior distributions used for estimation

| Parameter Name | Prior | Unit | Data Source |
| --- | --- | --- | --- |
| Initial ND [Non,BC] | Uniform (1120, 1401) | People | Data |
| Initial ND [Non,NBC] | Uniform (198, 297) | People | Data |
| Initial ND [Preg,BC] | Uniform (17, 25) | People | Data |
| Initial ND [Preg,NBC] | Uniform (83, 125) | People | Data |
| Initial NRD [Non,BC] | Uniform (3801, 4401) | People | Data |
| Initial NRD [Non,NBC] | Uniform (1056, 1584) | People | Data |
| Initial NRD [Preg,BC] | Uniform (17, 19) | People | Data |
| Initial NRD [Preg,NBC] | Uniform (83, 90) | People | Data |
| Initial RD [Non,BC] | Uniform (1462, 2193) | People | Data |
| Initial RD [Non,NBC] | Uniform (414, 622) | People | Data |
| Initial RD [Preg,BC] | Uniform (3, 5) | People | Data |
| Initial RD [Preg,NBC] | Uniform (17, 26) | People | Data |
| Initial AUD [Non,BC] | Uniform (597, 896) | People | Data |
| Initial AUD [Non,NBC] | Uniform (218, 327) | People | Data |
| Initial AUD [Preg,BC] | Uniform (4, 7) | People | Data |
| Initial AUD [Preg,NBC] | Uniform (24, 36) | People | Data |
| Initial Sober NRD [Non,BC] | Uniform (464, 696) | People | Data |
| Initial Sober NRD [Non,NBC] | Uniform (217, 325) | People | Data |
| Initial Sober NRD [Preg,BC] | Uniform (79, 118) | People | Data |
| Initial Sober NRD [Preg,NBC] | Uniform (372, 558) | People | Data |
| Initial Sober RD [Non,BC] | Uniform (87, 130) | People | Data |
| Initial Sober RD [Non,NBC] | Uniform (40, 61) | People | Data |
| Initial Sober RD [Preg,BC] | Uniform (28, 42) | People | Data |
| Initial Sober RD [Preg,NBC] | Uniform (140, 210) | People | Data |
| Initial Sober AUD [Non,BC] | Uniform (29, 43) | People | Data |
| Initial Sober AUD [Non,NBC] | Uniform (13, 20) | People | Data |
| Initial Sober AUD [Preg,BC] | Uniform (8, 13) | People | Data |
| Initial Sober AUD [Preg,NBC] | Uniform (43, 64) | People | Data |
| Initial AUD in Treatment [Non,BC] | Uniform (19, 29) | People | Data |
| Initial AUD in Treatment [Non,NBC] | Uniform (7, 10) | People | Data |
| Initial AUD in Treatment [Preg,BC] | Uniform (0, 1) | People | Data |
| Initial AUD in Treatment [Preg,NBC] | Uniform (4, 7) | People | Data |
| NRD Non to RD Non Fraction[BC] | Uniform (0.07, 0.15) | 1/Year | Literature |
| NRD Non to RD Non Fraction[NBC] | Uniform (0.07, 0.15) | 1/Year | Literature |
| NRD Non to Sober NRD Non Fraction[BC] | Uniform (0.04, 0.1) | 1/Year | Literature, modeling team judgement |
| NRD Non to Sober NRD Non Fraction[NBC] | Uniform (0.04, 0.1) | 1/Year | Literature, modeling team judgement |
| RD Non to NRD Non Fraction[BC] | Uniform (0.01, 0.2) | 1/Year | Literature, modeling team judgement |
| RD Non to NRD Non Fraction[NBC] | Uniform (0.01, 0.2) | 1/Year | Literature, modeling team judgement |
| RD Non to AUD Non Fraction[BC] | Uniform (0.03, 0.13) | 1/Year | Literature |
| RD Non to AUD Non Fraction[NBC] | Uniform (0.03, 0.13) | 1/Year | Literature |
| RD Non to Sober RD Non Fraction[BC] | Uniform (0.02, 0.2) | 1/Year | Literature |
| RD Non to Sober RD Non Fraction[NBC] | Uniform (0.02, 0.2) | 1/Year | Literature |
| AUD Non to NRD Non Fraction[BC] | Uniform (0.015, 0.05) | 1/Year | Literature |
| AUD Non to NRD Non Fraction[NBC] | Uniform (0.015, 0.05) | 1/Year | Literature |
| AUD Non to RD Non Fraction[BC] | Uniform (0.01, 0.066) | 1/Year | Literature |
| AUD Non to RD Non Fraction[NBC] | Uniform (0.01, 0.066) | 1/Year | Literature |
| AUD Non to Sober AUD Non Fraction[BC] | Uniform (0.015, 0.12) | 1/Year | Literature |
| AUD Non to Sober AUD Non Fraction[NBC] | Uniform (0.015, 0.12) | 1/Year | Literature |
| Sober NRD Non to NRD Non Fraction[BC] | Uniform (0.05, 0.25) | 1/Year | Literature, modeling team judgement |
| Sober NRD Non to NRD Non Fraction[NBC] | Uniform (0.05, 0.25) | 1/Year | Literature, modeling team judgement |
| Sober RD Non to NRD Non Fraction[BC] | Uniform (0.05, 0.2) | 1/Year | Literature |
| Sober RD Non to NRD Non Fraction[NBC] | Uniform (0.05, 0.2) | 1/Year | Literature |
| Sober RD Non to RD Non Fraction[BC] | Uniform (0.07, 0.2) | 1/Year | Literature |
| Sober RD Non to RD Non Fraction[NBC] | Uniform (0.07, 0.2) | 1/Year | Literature |
| Sober AUD Non to NRD Non Fraction[BC] | Uniform (0.02, 0.09) | 1/Year | Data, literature, modeling team judgement |
| Sober AUD Non to NRD Non Fraction[NBC] | Uniform (0.02, 0.09) | 1/Year | Data, literature, modeling team judgement |
| Sober AUD Non to RD Non Fraction[BC] | Uniform (0.02, 0.07) | 1/Year | Data, literature, modeling team judgement |
| Sober AUD Non to RD Non Fraction[NBC] | Uniform (0.02, 0.07) | 1/Year | Data, literature, modeling team judgement |
| Sober AUD Non to AUD Non Fraction[BC] | Uniform (0.05, 0.3) | 1/Year | Data, literature |
| Sober AUD Non to AUD Non Fraction[NBC] | Uniform (0.05, 0.3) | 1/Year | Data, literature |
| NRD Non Fraction Returning to Drinking After Birth[BC] | Uniform (0.2, 0.65) | Dimensionless | Literature |
| NRD Non Fraction Returning to Drinking After Birth[NBC] | Uniform (0.2, 0.65) | Dimensionless | Literature |
| RD Non Fraction Returning to Drinking After Birth[BC] | Uniform (0.3, 0.5) | Dimensionless | Literature |
| RD Non Fraction Returning to Drinking After Birth[NBC] | Uniform (0.3, 0.5) | Dimensionless | Literature |
| AUD Non Fraction Returning to Drinking After Birth[BC] | Uniform (0.3, 0.7) | Dimensionless | Literature |
| AUD Non Fraction Returning to Drinking After Birth[NBC] | Uniform (0.3, 0.7) | Dimensionless | Literature |
| NRD Non Time Spent Sober After Giving Birth[BC] | Uniform (0, 1.75) | Year | Literature |
| NRD Non Time Spent Sober After Giving Birth[NBC] | Uniform (0, 1.75) | Year | Literature |
| RD Non Time Spent Sober After Giving Birth[BC] | Uniform (0.25, 0.75) | Year | Literature |
| RD Non Time Spent Sober After Giving Birth[NBC] | Uniform (0.25, 0.75) | Year | Literature |
| AUD Non Time Spent Sober After Giving Birth[BC] | Uniform (0.25, 0.75) | Year | Literature |
| AUD Non Time Spent Sober After Giving Birth[NBC] | Uniform (0.25, 0.75) | Year | Literature |
| ND NBC to BC Fraction after Giving Birth | Uniform (0, 0.85) | Dimensionless | Data |
| NRD NBC to BC Fraction after Giving Birth | Uniform (0.7, 0.85) | Dimensionless | Data |
| RD NBC to BC Fraction after Giving Birth | Uniform (0.7, 0.85) | Dimensionless | Data |
| AUD NBC to BC Fraction after Giving Birth | Uniform (0.7, 0.85) | Dimensionless | Data |
| Sober NRD NBC to BC Fraction after Giving Birth | Uniform (0.7, 0.85) | Dimensionless | Data |
| Sober RD NBC to BC Fraction after Giving Birth | Uniform (0.7, 0.85) | Dimensionless | Data |
| Sober AUD NBC to BC Fraction after Giving Birth | Uniform (0.7, 0.85) | Dimensionless | Data |
| ND BC to NBC Fraction | Uniform (0.01, 0.9) | 1/Year | Data |
| ND NBC to BC Fraction | Uniform (0.05, 0.9) | 1/Year | Data |
| NRD BC to NBC Fraction | Uniform (0.01, 0.1) | 1/Year | Data |
| NRD NBC to BC Fraction | Uniform (0.05, 0.2) | 1/Year | Data |
| RD BC to NBC Fraction | Uniform (0.01, 0.1) | 1/Year | Data |
| RD NBC to BC Fraction | Uniform (0.05, 0.2) | 1/Year | Data |
| AUD BC to NBC Fraction | Uniform (0.01, 0.1) | 1/Year | Data |
| AUD NBC to BC Fraction | Uniform (0.05, 0.2) | 1/Year | Data |
| Sober NRD BC to NBC Fraction | Uniform (0.01, 0.15) | 1/Year | Data |
| Sober NRD NBC to BC Fraction | Uniform (0.05, 0.2) | 1/Year | Data |
| Sober RD BC to NBC Fraction | Uniform (0.01, 0.15) | 1/Year | Data |
| Sober RD NBC to BC Fraction | Uniform (0.05, 0.2) | 1/Year | Data |
| Sober AUD BC to NBC Fraction | Uniform (0.01, 0.15) | 1/Year | Data |
| Sober AUD NBC to BC Fraction | Uniform (0.05, 0.2) | 1/Year | Data |
| NRD Preg to Sober NRD Preg with Delay Initial Correction[BCBehavior] | Uniform (0, 100) | People/Year | Data |
| NRD Preg to Sober NRD Preg Immediately Initial Correction[BCBehavior] | Uniform (0, 100) | People/Year | Data |
| ND Aging or Moving In Uncertainty[BCBehavior] | Uniform (0, 1) | Dimensionless | Data, Modeler Team Judgement |
| ND Aging or Moving Out Uncertainty[BCBehavior] | Uniform (0, 0.5) | Dimensionless | Data, Modeler Team Judgement |
| ND Non to RD Non Uncertainty[BCBehavior] | Uniform (0, 1) | Dimensionless | Data, Modeler Team Judgement |
| ND Non to NRD Non Uncertainty[BCBehavior] | Uniform (0, 1) | Dimensionless | Data, Modeler Team Judgement |
| Sober NRD Aging or Moving Out Uncertainty[BCBehavior] | Uniform (0, 0.5) | Dimensionless | Data, Modeler Team Judgement |
| NRD Aging and Moving In Uncertainty[BCBehavior] | Uniform (0, 0.5) | Dimensionless | Data, Modeler Team Judgement |
| ESM1 | Uniform (0, 1) | Dimensionless | Modeler team judgement |
| ESM2 | Uniform (0, 1) | Dimensionless | Modeler team judgement |
| ESM3 | Uniform (0, 1) | Dimensionless | Modeler team judgement |
| ESM4 | Uniform (0, 1) | Dimensionless | Modeler team judgement |
| ESM5 | Uniform (0, 1) | Dimensionless | Modeler team judgement |
| ESM6 | Uniform (0, 1) | Dimensionless | Modeler team judgement |
| ESM7 | Uniform (0, 1) | Dimensionless | Modeler team judgement |
| ESM8 | Uniform (0, 1) | Dimensionless | Modeler team judgement |
| ESM9 | Uniform (0, 1) | Dimensionless | Modeler team judgement |
| ESM10 | Uniform (0, 1) | Dimensionless | Modeler team judgement |
| ESM11 | Uniform (0, 1) | Dimensionless | Modeler team judgement |
| ESM12 | Uniform (0, 1) | Dimensionless | Modeler team judgement |
| ESM13 | Uniform (0, 1) | Dimensionless | Modeler team judgement |
| ESM14 | Uniform (0, 1) | Dimensionless | Modeler team judgement |
| ESM15 | Uniform (0, 1) | Dimensionless | Modeler team judgement |
| ESM16 | Uniform (0, 1) | Dimensionless | Modeler team judgement |
| ESM17 | Uniform (0, 1) | Dimensionless | Modeler team judgement |
| ESM18 | Uniform (0, 1) | Dimensionless | Modeler team judgement |
| ESM19 | Uniform (0, 1) | Dimensionless | Modeler team judgement |
| ESM20 | Uniform (0, 1) | Dimensionless | Modeler team judgement |
| ESM21 | Uniform (0, 1) | Dimensionless | Modeler team judgement |
| ESM22 | Uniform (0, 1) | Dimensionless | Modeler team judgement |
| ESM23 | Uniform (0, 1) | Dimensionless | Modeler team judgement |
| ESM24 | Uniform (0, 1) | Dimensionless | Modeler team judgement |
| ESM25 | Uniform (0, 1) | Dimensionless | Modeler team judgement |
| ESM26 | Uniform (0, 1) | Dimensionless | Modeler team judgement |
| ESM27 | Uniform (0, 1) | Dimensionless | Modeler team judgement |
| ESM28 | Uniform (0, 1) | Dimensionless | Modeler team judgement |
| ESM29 | Uniform (0, 1) | Dimensionless | Modeler team judgement |
| ESM30 | Uniform (0, 1) | Dimensionless | Modeler team judgement |
| ESM31 | Uniform (0, 1) | Dimensionless | Modeler team judgement |
| ESM32 | Uniform (0, 1) | Dimensionless | Modeler team judgement |

BCBehavior includes both BC and NBC categories. ESM parameters capture the estimated standard deviation of measurement noise for each time series.

# Supplement B2. Estimated parameters and posterior samples

We first used Powell’s derivative-free direction-set method to locate informative regions of the parameter space. To reduce the chance of local optima, the search was run multiple times from diverse starting points. Using the best points identified by Powell, we then performed Markov chain Monte Carlo (MCMC), running two independent chains. We discarded the first 10,000 iterations of each chain as burn-in to allow the chains to move away from their initial values and into the high-density region of the parameter space before sampling. We then collected 10,000 posterior draws for inference. Supplement B2 Table 1 reports the summary statistics of the posterior samples.

Supplement B2. Table 1. Summary statistics of the posterior parameter distributions

| Parameter Name | Mean | Median | MAP | 95% CI |
| --- | --- | --- | --- | --- |
| Initial ND[Non,BC] | 1177.255 | 1177.204 | 1177.195 | (1175.240, 1179.659) |
| Initial ND[Non,NBC] | 253.423 | 253.428 | 253.448 | (252.159, 254.502) |
| Initial ND[Preg,BC] | 24.258 | 24.257 | 24.253 | (24.164, 24.354) |
| Initial ND[Preg,NBC] | 125.179 | 125.198 | 125.214 | (124.722, 125.522) |
| Initial NRD[Non,BC] | 4401.385 | 4401.593 | 4401.904 | (4399.746, 4401.971) |
| Initial NRD[Non,NBC] | 1309.672 | 1309.703 | 1309.768 | (1305.425, 1314.286) |
| Initial NRD[Preg,BC] | 19.553 | 19.553 | 19.554 | (19.548, 19.554) |
| Initial NRD[Preg,NBC] | 90.491 | 90.494 | 90.497 | (90.467, 90.498) |
| Initial RD[Non,BC] | 1815.557 | 1815.585 | 1815.512 | (1807.950, 1822.628) |
| Initial RD[Non,NBC] | 521.340 | 521.404 | 521.528 | (519.131, 523.219) |
| Initial RD[Preg,BC] | 3.700 | 3.700 | 3.700 | (3.681, 3.725) |
| Initial RD[Preg,NBC] | 19.576 | 19.577 | 19.575 | (19.466, 19.668) |
| Initial AUD[Non,BC] | 753.457 | 753.429 | 753.411 | (750.348, 756.732) |
| Initial AUD[Non,NBC] | 277.126 | 277.136 | 277.166 | (276.139, 278.050) |
| Initial AUD[Preg,BC] | 5.839 | 5.839 | 5.838 | (5.819, 5.859) |
| Initial AUD[Preg,NBC] | 28.391 | 28.392 | 28.391 | (28.235, 28.529) |
| Initial Sober NRD[Non,BC] | 464.732 | 464.667 | 464.535 | (464.532, 465.342) |
| Initial Sober NRD[Non,NBC] | 270.596 | 270.600 | 270.618 | (269.632, 271.638) |
| Initial Sober NRD[Preg,BC] | 95.517 | 95.519 | 95.504 | (95.073, 95.913) |
| Initial Sober NRD[Preg,NBC] | 444.897 | 444.880 | 444.853 | (442.721, 447.545) |
| Initial Sober RD[Non,BC] | 95.297 | 95.297 | 95.309 | (94.878, 95.769) |
| Initial Sober RD[Non,NBC] | 50.772 | 50.776 | 50.777 | (50.500, 50.992) |
| Initial Sober RD[Preg,BC] | 29.245 | 29.240 | 29.234 | (29.075, 29.410) |
| Initial Sober RD[Preg,NBC] | 149.598 | 149.608 | 149.579 | (148.688, 150.350) |
| Initial Sober AUD[Non,BC] | 29.044 | 29.040 | 29.038 | (29.033, 29.076) |
| Initial Sober AUD[Non,NBC] | 16.966 | 16.969 | 16.971 | (16.881, 17.031) |
| Initial Sober AUD[Preg,BC] | 11.459 | 11.458 | 11.457 | (11.426, 11.492) |
| Initial Sober AUD[Preg,NBC] | 44.316 | 44.312 | 44.321 | (44.116, 44.513) |
| Initial AUD in Treatment[Non,BC] | 22.748 | 22.750 | 22.747 | (22.658, 22.820) |
| Initial AUD in Treatment[Non,NBC] | 8.975 | 8.975 | 8.976 | (8.919, 9.025) |
| Initial AUD in Treatment[Preg,BC] | 1.143 | 1.142 | 1.142 | (1.137, 1.148) |
| Initial AUD in Treatment[Preg,NBC] | 5.505 | 5.505 | 5.505 | (5.483, 5.536) |
| NRD Non to RD Non Fraction[BC] | 0.070 | 0.070 | 0.070 | (0.070, 0.070) |
| NRD Non to RD Non Fraction[NBC] | 0.070 | 0.070 | 0.070 | (0.070, 0.070) |
| NRD Non to Sober NRD Non Fraction[BC] | 0.049 | 0.049 | 0.049 | (0.048, 0.050) |
| NRD Non to Sober NRD Non Fraction[NBC] | 0.042 | 0.042 | 0.042 | (0.041, 0.043) |
| RD Non to NRD Non Fraction[BC] | 0.200 | 0.200 | 0.200 | (0.199, 0.200) |
| RD Non to NRD Non Fraction[NBC] | 0.200 | 0.200 | 0.200 | (0.199, 0.200) |
| RD Non to AUD Non Fraction[BC] | 0.030 | 0.030 | 0.030 | (0.030, 0.031) |
| RD Non to AUD Non Fraction[NBC] | 0.091 | 0.091 | 0.091 | (0.090, 0.092) |
| RD Non to Sober RD Non Fraction[BC] | 0.026 | 0.026 | 0.026 | (0.024, 0.028) |
| RD Non to Sober RD Non Fraction[NBC] | 0.023 | 0.023 | 0.023 | (0.022, 0.025) |
| AUD Non to NRD Non Fraction[BC] | 0.050 | 0.050 | 0.050 | (0.050, 0.050) |
| AUD Non to NRD Non Fraction[NBC] | 0.050 | 0.050 | 0.050 | (0.050, 0.050) |
| AUD Non to RD Non Fraction[BC] | 0.014 | 0.014 | 0.014 | (0.013, 0.016) |
| AUD Non to RD Non Fraction[NBC] | 0.038 | 0.038 | 0.038 | (0.037, 0.039) |
| AUD Non to Sober AUD Non Fraction[BC] | 0.015 | 0.015 | 0.015 | (0.015, 0.016) |
| AUD Non to Sober AUD Non Fraction[NBC] | 0.015 | 0.015 | 0.015 | (0.015, 0.015) |
| Sober NRD Non to NRD Non Fraction[BC] | 0.250 | 0.250 | 0.250 | (0.249, 0.250) |
| Sober NRD Non to NRD Non Fraction[NBC] | 0.157 | 0.157 | 0.157 | (0.155, 0.159) |
| Sober RD Non to NRD Non Fraction[BC] | 0.200 | 0.200 | 0.200 | (0.199, 0.200) |
| Sober RD Non to NRD Non Fraction[NBC] | 0.200 | 0.200 | 0.200 | (0.199, 0.200) |
| Sober RD Non to RD Non Fraction[BC] | 0.159 | 0.159 | 0.159 | (0.157, 0.160) |
| Sober RD Non to RD Non Fraction[NBC] | 0.200 | 0.200 | 0.200 | (0.199, 0.200) |
| Sober AUD Non to NRD Non Fraction[BC] | 0.090 | 0.090 | 0.090 | (0.089, 0.090) |
| Sober AUD Non to NRD Non Fraction[NBC] | 0.090 | 0.090 | 0.090 | (0.090, 0.090) |
| Sober AUD Non to RD Non Fraction[BC] | 0.020 | 0.020 | 0.020 | (0.020, 0.020) |
| Sober AUD Non to RD Non Fraction[NBC] | 0.070 | 0.070 | 0.070 | (0.070, 0.070) |
| Sober AUD Non to AUD Non Fraction[BC] | 0.162 | 0.162 | 0.162 | (0.160, 0.165) |
| Sober AUD Non to AUD Non Fraction[NBC] | 0.300 | 0.300 | 0.300 | (0.299, 0.300) |
| NRD Non Fraction Returning to Drinking After Birth[BC] | 0.450 | 0.450 | 0.450 | (0.445, 0.454) |
| NRD Non Fraction Returning to Drinking After Birth[NBC] | 0.270 | 0.270 | 0.270 | (0.265, 0.277) |
| RD Non Fraction Returning to Drinking After Birth[BC] | 0.500 | 0.500 | 0.500 | (0.499, 0.500) |
| RD Non Fraction Returning to Drinking After Birth[NBC] | 0.429 | 0.429 | 0.429 | (0.427, 0.431) |
| AUD Non Fraction Returning to Drinking After Birth[BC] | 0.607 | 0.607 | 0.607 | (0.603, 0.611) |
| AUD Non Fraction Returning to Drinking After Birth[NBC] | 0.669 | 0.669 | 0.669 | (0.664, 0.675) |
| NRD Non Time Spent Sober After Giving Birth[BC] | 0.499 | 0.500 | 0.501 | (0.476, 0.518) |
| NRD Non Time Spent Sober After Giving Birth[NBC] | 0.501 | 0.501 | 0.501 | (0.487, 0.521) |
| RD Non Time Spent Sober After Giving Birth[BC] | 0.750 | 0.750 | 0.750 | (0.748, 0.750) |
| RD Non Time Spent Sober After Giving Birth[NBC] | 0.750 | 0.750 | 0.750 | (0.749, 0.750) |
| AUD Non Time Spent Sober After Giving Birth[BC] | 0.749 | 0.750 | 0.750 | (0.748, 0.750) |
| AUD Non Time Spent Sober After Giving Birth[NBC] | 0.749 | 0.750 | 0.750 | (0.748, 0.750) |
| ND NBC to BC Fraction after Giving Birth | 0.849 | 0.849 | 0.850 | (0.847, 0.850) |
| NRD NBC to BC Fraction after Giving Birth | 0.786 | 0.786 | 0.786 | (0.783, 0.788) |
| RD NBC to BC Fraction after Giving Birth | 0.700 | 0.700 | 0.700 | (0.700, 0.701) |
| AUD NBC to BC Fraction after Giving Birth | 0.850 | 0.850 | 0.850 | (0.849, 0.850) |
| Sober NRD NBC to BC Fraction after Giving Birth | 0.850 | 0.850 | 0.850 | (0.849, 0.850) |
| Sober RD NBC to BC Fraction after Giving Birth | 0.850 | 0.850 | 0.850 | (0.849, 0.850) |
| Sober AUD NBC to BC Fraction after Giving Birth | 0.726 | 0.726 | 0.726 | (0.724, 0.729) |
| ND BC to NBC Fraction | 0.046 | 0.046 | 0.046 | (0.035, 0.054) |
| ND NBC to BC Fraction | 0.051 | 0.050 | 0.050 | (0.050, 0.052) |
| NRD BC to NBC Fraction | 0.100 | 0.100 | 0.100 | (0.099, 0.100) |
| NRD NBC to BC Fraction | 0.138 | 0.137 | 0.138 | (0.136, 0.139) |
| RD BC to NBC Fraction | 0.091 | 0.091 | 0.091 | (0.090, 0.092) |
| RD NBC to BC Fraction | 0.050 | 0.050 | 0.050 | (0.050, 0.051) |
| AUD BC to NBC Fraction | 0.090 | 0.090 | 0.090 | (0.089, 0.092) |
| AUD NBC to BC Fraction | 0.050 | 0.050 | 0.050 | (0.050, 0.051) |
| Sober NRD BC to NBC Fraction | 0.072 | 0.072 | 0.072 | (0.070, 0.073) |
| Sober NRD NBC to BC Fraction | 0.174 | 0.174 | 0.174 | (0.172, 0.176) |
| Sober RD BC to NBC Fraction | 0.150 | 0.150 | 0.150 | (0.149, 0.150) |
| Sober RD NBC to BC Fraction | 0.134 | 0.134 | 0.134 | (0.132, 0.136) |
| Sober AUD BC to NBC Fraction | 0.085 | 0.085 | 0.085 | (0.083, 0.087) |
| Sober AUD NBC to BC Fraction | 0.200 | 0.200 | 0.200 | (0.199, 0.200) |
| NRD Preg to Sober NRD Preg with Delay Initial Correction[BC] | 4.659 | 4.676 | 4.638 | (3.742, 5.425) |
| NRD Preg to Sober NRD Preg with Delay Initial Correction[NBC] | 21.308 | 21.291 | 21.303 | (20.236, 22.523) |
| NRD Preg to Sober NRD Preg Immediately Initial Correction[BC] | 0.080 | 0.054 | 0.004 | (0.002, 0.336) |
| NRD Preg to Sober NRD Preg Immediately Initial Correction[NBC] | 0.095 | 0.058 | 0.004 | (0.003, 0.408) |
| ND Aging or Moving In Uncertainty[BC] | 0.307 | 0.307 | 0.307 | (0.297, 0.319) |
| ND Aging or Moving In Uncertainty[NBC] | 0.999 | 0.999 | 1.000 | (0.997, 1.000) |
| ND Aging or Moving Out Uncertainty[BC] | 0.000 | 0.000 | 0.000 | (0.000, 0.002) |
| ND Aging or Moving Out Uncertainty[NBC] | 0.500 | 0.500 | 0.500 | (0.498, 0.500) |
| ND Non to RD Non Uncertainty[BC] | 0.001 | 0.000 | 0.000 | (0.000, 0.003) |
| ND Non to RD Non Uncertainty[NBC] | 0.001 | 0.001 | 0.000 | (0.000, 0.004) |
| ND Non to NRD Non Uncertainty[BC] | 0.001 | 0.000 | 0.000 | (0.000, 0.002) |
| ND Non to NRD Non Uncertainty[NBC] | 0.001 | 0.001 | 0.000 | (0.000, 0.003) |
| Sober NRD Aging or Moving Out Uncertainty[BC] | 0.499 | 0.500 | 0.500 | (0.498, 0.500) |
| Sober NRD Aging or Moving Out Uncertainty[NBC] | 0.332 | 0.332 | 0.332 | (0.325, 0.338) |
| NRD Aging and Moving In Uncertainty[BC] | 0.000 | 0.000 | 0.000 | (0.000, 0.002) |
| NRD Aging and Moving In Uncertainty[NBC] | 0.000 | 0.000 | 0.000 | (0.000, 0.002) |
| ESM1 | 0.054 | 0.055 | 0.054 | (0.044, 0.062) |
| ESM2 | 0.094 | 0.094 | 0.093 | (0.083, 0.104) |
| ESM3 | 0.081 | 0.081 | 0.081 | (0.068, 0.092) |
| ESM4 | 0.113 | 0.112 | 0.112 | (0.105, 0.123) |
| ESM5 | 0.014 | 0.013 | 0.013 | (0.006, 0.026) |
| ESM6 | 0.014 | 0.014 | 0.014 | (0.007, 0.023) |
| ESM7 | 0.155 | 0.155 | 0.155 | (0.144, 0.163) |
| ESM8 | 0.150 | 0.150 | 0.150 | (0.137, 0.162) |
| ESM9 | 0.015 | 0.015 | 0.016 | (0.007, 0.024) |
| ESM10 | 0.018 | 0.018 | 0.018 | (0.009, 0.031) |
| ESM11 | 0.255 | 0.255 | 0.256 | (0.244, 0.265) |
| ESM12 | 0.208 | 0.208 | 0.208 | (0.200, 0.218) |
| ESM13 | 0.031 | 0.031 | 0.030 | (0.021, 0.043) |
| ESM14 | 0.033 | 0.032 | 0.032 | (0.019, 0.047) |
| ESM15 | 0.160 | 0.160 | 0.161 | (0.152, 0.170) |
| ESM16 | 0.160 | 0.160 | 0.160 | (0.149, 0.171) |
| ESM17 | 0.255 | 0.255 | 0.255 | (0.245, 0.263) |
| ESM18 | 0.009 | 0.009 | 0.009 | (0.004, 0.018) |
| ESM19 | 0.086 | 0.086 | 0.086 | (0.074, 0.097) |
| ESM20 | 0.112 | 0.112 | 0.112 | (0.098, 0.122) |
| ESM21 | 0.111 | 0.111 | 0.111 | (0.100, 0.123) |
| ESM22 | 0.006 | 0.006 | 0.005 | (0.003, 0.014) |
| ESM23 | 0.137 | 0.137 | 0.136 | (0.127, 0.146) |
| ESM24 | 0.111 | 0.111 | 0.111 | (0.101, 0.118) |
| ESM25 | 0.386 | 0.386 | 0.386 | (0.375, 0.400) |
| ESM26 | 0.012 | 0.011 | 0.012 | (0.006, 0.020) |
| ESM27 | 0.147 | 0.147 | 0.147 | (0.137, 0.156) |
| ESM28 | 0.218 | 0.218 | 0.218 | (0.209, 0.229) |
| ESM29 | 0.266 | 0.265 | 0.265 | (0.254, 0.281) |
| ESM30 | 0.324 | 0.324 | 0.324 | (0.313, 0.338) |
| ESM31 | 0.143 | 0.142 | 0.142 | (0.134, 0.154) |
| ESM32 | 0.155 | 0.155 | 0.155 | (0.140, 0.164) |

MAP denotes Maximum a Posteriori. We used the mean predicted values to run the simulation model for further analysis.

For most parameters, posterior estimates provide a narrower interval compared to the prior distributions. In addition, the posterior estimates offer relatively confident intervals. The full posterior samples obtained through the Monte Carlo process are reported in MCMC_Gaussian_v01_MCMC_sample.tab. One can use the posterior samples and plot the marginal and bivariate posterior distributions of the parameters. Figure B2.1 shows an example of such an attempt. A qualitative investigation of this specific example indicates no huge correlation between the parameters; however, such cases might be observed through a full analysis of the samples. Because we have a high dimensional problem, we do not show the joint distributions of all parameters and limit our report to these few examples provided in the table and the figure, though we encourage readers to further explore the actual samples provided in the supplementary materials.


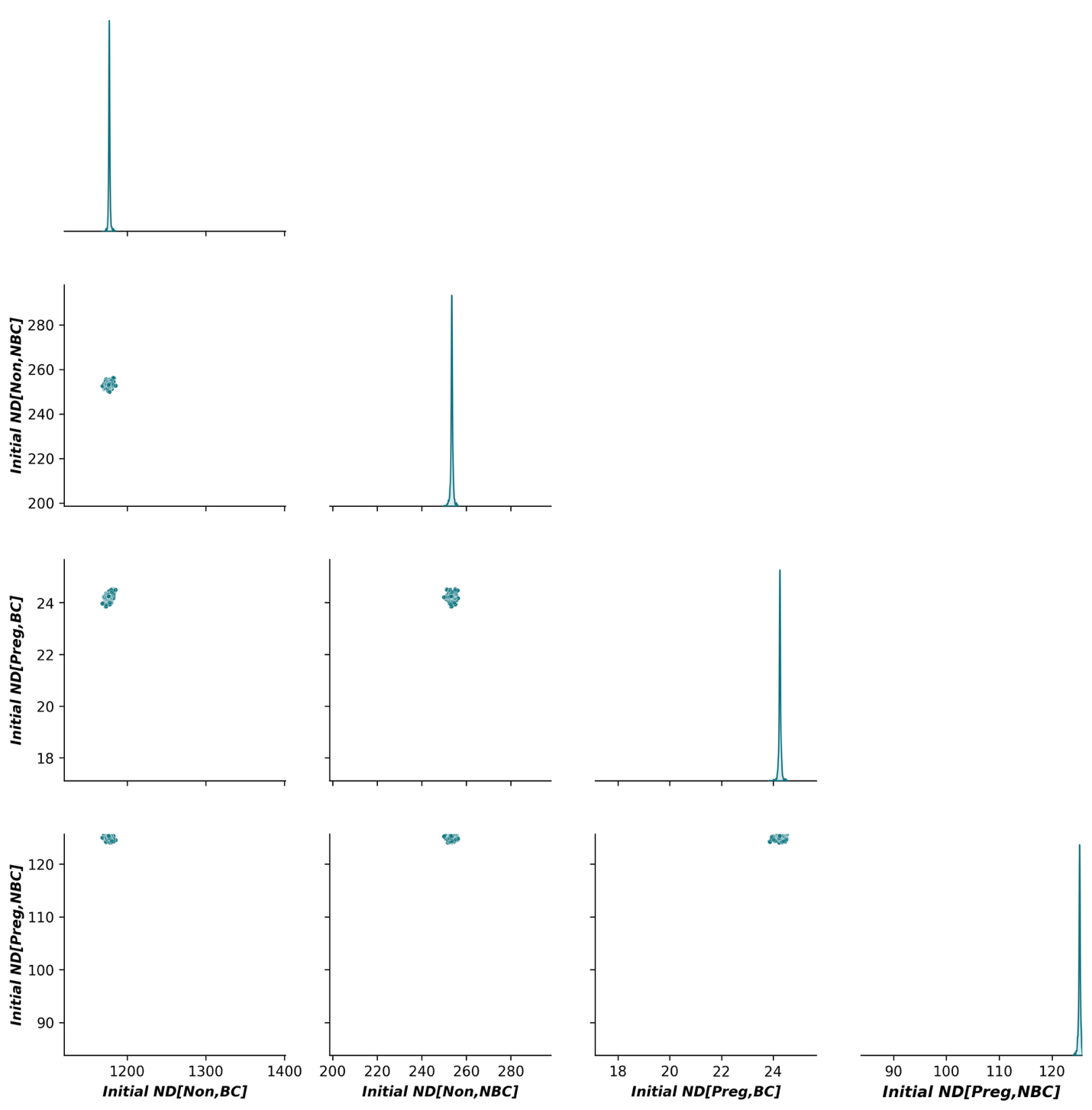


Figure B2.1. Joint posterior distributions using 10,000 posterior samples obtained through MCMC.

# Supplement B3. Posterior predictive checks and fit to data

One can use the posterior samples and run the simulation model iteratively to obtain the posterior predictive checks of the simulation results. Figure 3 shows how well the target data fall within different credible levels of the posterior predictive checks.

Supplement B3 Table 1 documents the predicted mean and standard deviation of the simulated values generated through the posterior samples against the target data for each available time point.

Supplement B3. Table 2 reports goodness-of-fit statistics comparing the model-predicted mean trajectories with the observed targets: Mean Absolute Error (MAE), Root Mean Square Error (RMSE), and Normalized RMSE (NRMSE; RMSE divided by the standard deviation of the simulated series). These complementary metrics provide a scale-aware summary of in-sample fit (lower values indicate closer agreement), allowing us to judge whether the calibrated simulator credibly reproduces the observed levels and trends in the data.


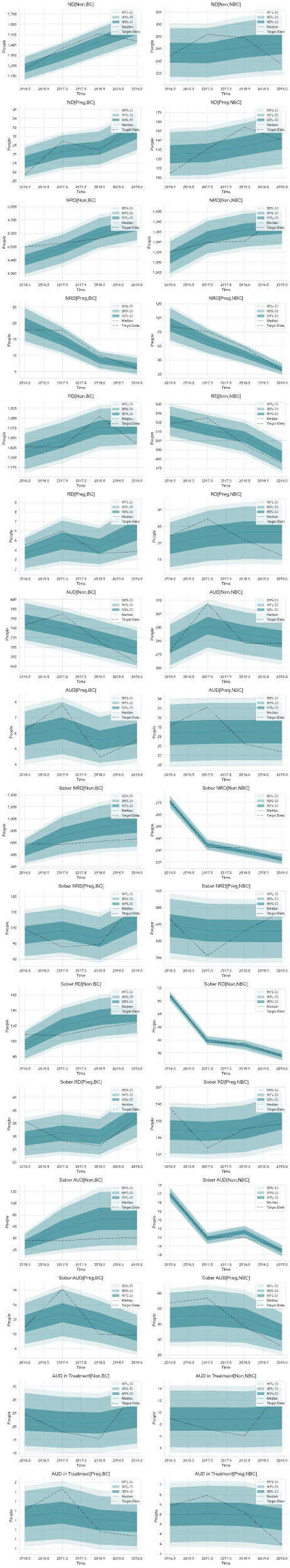


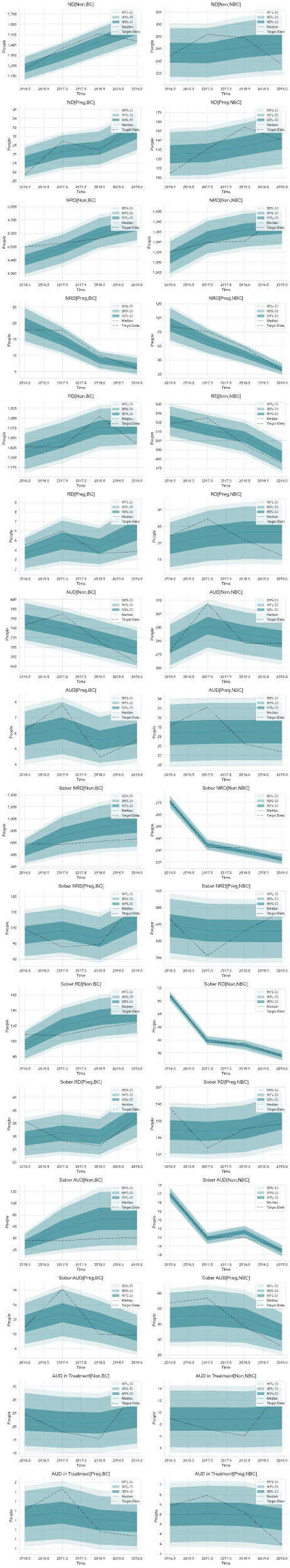


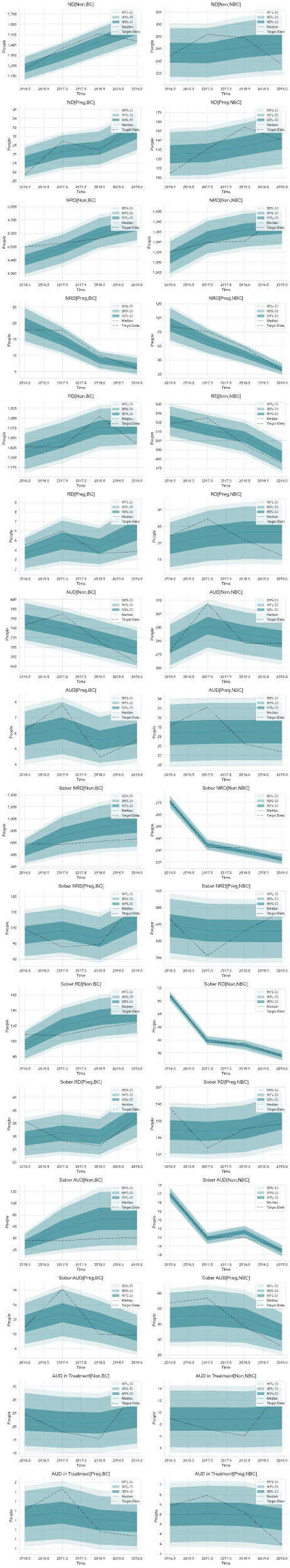


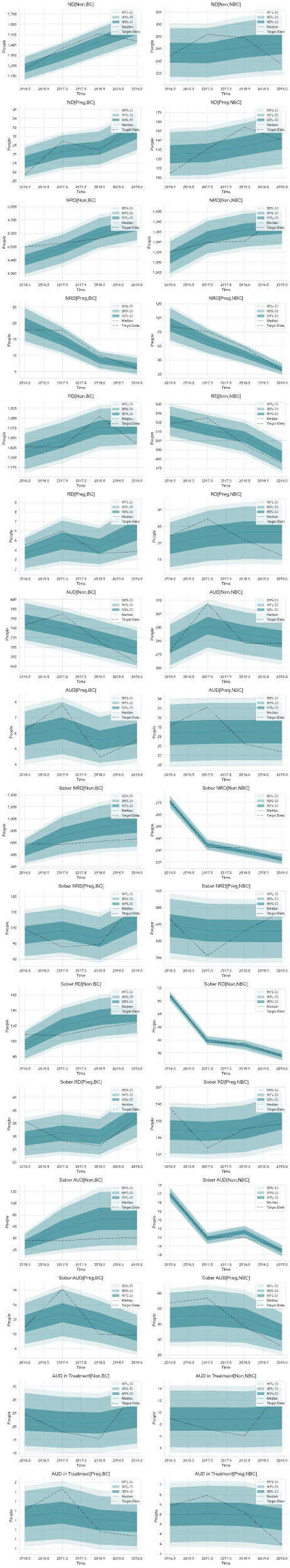


Figure B3.1. Posterior predictive checks obtained from running the simulation model using 10,000 posterior samples.

Supplement B3. Table 1. Comparisons between observed and simulated values

| Variable Name | Time | Observed Data Value | Predicted Simulated Mean | Predicted Simulated Standard Deviation |
| --- | --- | --- | --- | --- |
| ND [Non,BC] | 2016 | 1167.720 | 1176.821 | 64.581 |
| ND [Non,BC] | 2017 | 1369.540 | 1297.206 | 70.750 |
| ND [Non,BC] | 2018 | 1511.740 | 1433.901 | 79.426 |
| ND [Non,BC] | 2019 | 1439.850 | 1559.671 | 85.403 |
| ND [Non,NBC] | 2016 | 248.330 | 253.584 | 23.780 |
| ND [Non,NBC] | 2017 | 283.600 | 253.034 | 24.217 |
| ND [Non,NBC] | 2018 | 277.930 | 263.206 | 25.533 |
| ND [Non,NBC] | 2019 | 234.010 | 269.005 | 26.415 |
| ND [Preg,BC] | 2016 | 21.400 | 24.217 | 1.952 |
| ND [Preg,BC] | 2017 | 28.870 | 26.619 | 2.151 |
| ND [Preg,BC] | 2018 | 26.870 | 27.374 | 2.219 |
| ND [Preg,BC] | 2019 | 33.220 | 31.094 | 2.505 |
| ND [Preg,NBC] | 2016 | 104.730 | 125.195 | 13.958 |
| ND [Preg,NBC] | 2017 | 130.450 | 126.570 | 14.366 |
| ND [Preg,NBC] | 2018 | 154.080 | 134.039 | 14.884 |
| ND [Preg,NBC] | 2019 | 140.330 | 141.479 | 16.063 |
| NRD [Non,BC] | 2016 | 4501.660 | 4401.221 | 64.088 |
| NRD [Non,BC] | 2017 | 4526.250 | 4485.174 | 65.787 |
| NRD [Non,BC] | 2018 | 4562.030 | 4604.504 | 67.212 |
| NRD [Non,BC] | 2019 | 4652.800 | 4660.085 | 68.736 |
| NRD [Non,NBC] | 2016 | 1320.480 | 1309.566 | 19.432 |
| NRD [Non,NBC] | 2017 | 1338.820 | 1349.091 | 19.591 |
| NRD [Non,NBC] | 2018 | 1341.620 | 1363.600 | 20.205 |
| NRD [Non,NBC] | 2019 | 1393.990 | 1369.123 | 20.320 |
| NRD [Preg,BC] | 2016 | 17.960 | 19.564 | 3.035 |
| NRD [Preg,BC] | 2017 | 17.850 | 14.777 | 2.311 |
| NRD [Preg,BC] | 2018 | 10.190 | 8.596 | 1.531 |
| NRD [Preg,BC] | 2019 | 5.970 | 6.749 | 1.556 |
| NRD [Preg,NBC] | 2016 | 87.910 | 90.721 | 13.609 |
| NRD [Preg,NBC] | 2017 | 80.640 | 67.402 | 10.233 |
| NRD [Preg,NBC] | 2018 | 58.430 | 48.182 | 7.282 |
| NRD [Preg,NBC] | 2019 | 25.200 | 27.437 | 4.478 |
| RD [Non,BC] | 2016 | 1828.020 | 1815.489 | 28.327 |
| RD [Non,BC] | 2017 | 1827.600 | 1843.102 | 28.648 |
| RD [Non,BC] | 2018 | 1904.230 | 1876.010 | 29.163 |
| RD [Non,BC] | 2019 | 1834.420 | 1879.362 | 29.108 |
| RD [Non,NBC] | 2016 | 518.410 | 521.392 | 9.847 |
| RD [Non,NBC] | 2017 | 525.110 | 512.049 | 9.556 |
| RD [Non,NBC] | 2018 | 495.240 | 504.052 | 9.684 |
| RD [Non,NBC] | 2019 | 475.950 | 482.752 | 9.303 |
| RD [Preg,BC] | 2016 | 4.540 | 3.697 | 0.946 |
| RD [Preg,BC] | 2017 | 6.020 | 4.979 | 1.278 |
| RD [Preg,BC] | 2018 | 3.650 | 4.407 | 1.135 |
| RD [Preg,BC] | 2019 | 3.850 | 6.092 | 1.550 |
| RD [Preg,NBC] | 2016 | 22.210 | 19.536 | 4.082 |
| RD [Preg,NBC] | 2017 | 27.210 | 22.042 | 4.582 |
| RD [Preg,NBC] | 2018 | 20.930 | 23.187 | 4.760 |
| RD [Preg,NBC] | 2019 | 16.240 | 23.185 | 4.855 |
| AUD [Non,BC] | 2016 | 747.170 | 753.235 | 23.496 |
| AUD [Non,BC] | 2017 | 769.430 | 737.790 | 23.034 |
| AUD [Non,BC] | 2018 | 710.740 | 718.415 | 22.470 |
| AUD [Non,BC] | 2019 | 668.500 | 698.519 | 22.096 |
| AUD [Non,NBC] | 2016 | 272.580 | 276.943 | 9.201 |
| AUD [Non,NBC] | 2017 | 307.530 | 291.303 | 9.834 |
| AUD [Non,NBC] | 2018 | 278.180 | 285.375 | 9.622 |
| AUD [Non,NBC] | 2019 | 278.260 | 281.370 | 9.611 |
| AUD [Preg,BC] | 2016 | 6.210 | 5.851 | 0.937 |
| AUD [Preg,BC] | 2017 | 7.820 | 6.338 | 1.011 |
| AUD [Preg,BC] | 2018 | 4.460 | 5.567 | 0.890 |
| AUD [Preg,BC] | 2019 | 5.550 | 5.993 | 0.966 |
| AUD [Preg,NBC] | 2016 | 30.370 | 28.399 | 4.571 |
| AUD [Preg,NBC] | 2017 | 35.310 | 28.732 | 4.622 |
| AUD [Preg,NBC] | 2018 | 25.570 | 28.795 | 4.579 |
| AUD [Preg,NBC] | 2019 | 23.460 | 28.817 | 4.585 |
| Sober NRD [Non,BC] | 2016 | 580.660 | 463.620 | 119.071 |
| Sober NRD [Non,BC] | 2017 | 572.470 | 724.746 | 184.278 |
| Sober NRD [Non,BC] | 2018 | 625.090 | 857.018 | 216.265 |
| Sober NRD [Non,BC] | 2019 | 661.770 | 931.756 | 237.385 |
| Sober NRD [Non,NBC] | 2016 | 271.310 | 270.570 | 2.794 |
| Sober NRD [Non,NBC] | 2017 | 232.540 | 234.669 | 2.468 |
| Sober NRD [Non,NBC] | 2018 | 232.000 | 228.879 | 2.539 |
| Sober NRD [Non,NBC] | 2019 | 221.250 | 222.730 | 2.600 |
| Sober NRD [Preg,BC] | 2016 | 99.110 | 95.410 | 8.218 |
| Sober NRD [Preg,BC] | 2017 | 87.780 | 98.305 | 8.415 |
| Sober NRD [Preg,BC] | 2018 | 89.310 | 93.359 | 8.116 |
| Sober NRD [Preg,BC] | 2019 | 117.430 | 104.716 | 9.046 |
| Sober NRD [Preg,NBC] | 2016 | 465.110 | 445.732 | 49.622 |
| Sober NRD [Preg,NBC] | 2017 | 356.310 | 431.399 | 48.578 |
| Sober NRD [Preg,NBC] | 2018 | 430.240 | 429.835 | 48.909 |
| Sober NRD [Preg,NBC] | 2019 | 486.440 | 429.204 | 48.234 |
| Sober RD [Non,BC] | 2016 | 108.870 | 95.219 | 10.529 |
| Sober RD [Non,BC] | 2017 | 107.340 | 120.036 | 13.348 |
| Sober RD [Non,BC] | 2018 | 117.210 | 130.914 | 14.601 |
| Sober RD [Non,BC] | 2019 | 124.080 | 135.723 | 15.100 |
| Sober RD [Non,NBC] | 2016 | 50.870 | 50.769 | 0.403 |
| Sober RD [Non,NBC] | 2017 | 43.600 | 43.910 | 0.381 |
| Sober RD [Non,NBC] | 2018 | 43.500 | 43.197 | 0.421 |
| Sober RD [Non,NBC] | 2019 | 41.480 | 41.605 | 0.438 |
| Sober RD [Preg,BC] | 2016 | 35.800 | 29.187 | 3.983 |
| Sober RD [Preg,BC] | 2017 | 28.230 | 31.240 | 4.300 |
| Sober RD [Preg,BC] | 2018 | 26.270 | 29.867 | 4.124 |
| Sober RD [Preg,BC] | 2019 | 37.020 | 38.181 | 5.170 |
| Sober RD [Preg,NBC] | 2016 | 175.240 | 149.310 | 16.713 |
| Sober RD [Preg,NBC] | 2017 | 127.530 | 147.700 | 16.474 |
| Sober RD [Preg,NBC] | 2018 | 150.660 | 150.715 | 16.945 |
| Sober RD [Preg,NBC] | 2019 | 156.400 | 162.288 | 18.027 |
| Sober AUD [Non,BC] | 2016 | 36.290 | 29.046 | 10.843 |
| Sober AUD [Non,BC] | 2017 | 35.780 | 55.969 | 20.986 |
| Sober AUD [Non,BC] | 2018 | 39.070 | 73.267 | 27.346 |
| Sober AUD [Non,BC] | 2019 | 41.360 | 73.637 | 27.370 |
| Sober AUD [Non,NBC] | 2016 | 16.960 | 16.966 | 0.215 |
| Sober AUD [Non,NBC] | 2017 | 14.530 | 14.448 | 0.182 |
| Sober AUD [Non,NBC] | 2018 | 14.500 | 14.824 | 0.194 |
| Sober AUD [Non,NBC] | 2019 | 13.830 | 13.736 | 0.183 |
| Sober AUD [Preg,BC] | 2016 | 10.990 | 11.437 | 1.691 |
| Sober AUD [Preg,BC] | 2017 | 16.150 | 12.915 | 1.915 |
| Sober AUD [Preg,BC] | 2018 | 9.960 | 11.743 | 1.714 |
| Sober AUD [Preg,BC] | 2019 | 9.890 | 10.098 | 1.491 |
| Sober AUD [Preg,NBC] | 2016 | 53.780 | 44.303 | 9.648 |
| Sober AUD [Preg,NBC] | 2017 | 56.820 | 45.634 | 9.996 |
| Sober AUD [Preg,NBC] | 2018 | 38.400 | 43.675 | 9.471 |
| Sober AUD [Preg,NBC] | 2019 | 25.500 | 34.665 | 7.504 |
| AUD in Treatment [Non,BC] | 2016 | 24.550 | 22.637 | 6.035 |
| AUD in Treatment [Non,BC] | 2017 | 17.960 | 21.630 | 5.776 |
| AUD in Treatment [Non,BC] | 2018 | 15.500 | 21.321 | 5.666 |
| AUD in Treatment [Non,BC] | 2019 | 33.790 | 23.853 | 6.230 |
| AUD in Treatment [Non,NBC] | 2016 | 8.960 | 8.891 | 2.871 |
| AUD in Treatment [Non,NBC] | 2017 | 7.180 | 8.889 | 2.870 |
| AUD in Treatment [Non,NBC] | 2018 | 6.070 | 8.834 | 2.848 |
| AUD in Treatment [Non,NBC] | 2019 | 14.060 | 9.174 | 2.931 |
| AUD in Treatment [Preg,BC] | 2016 | 1.210 | 1.142 | 0.162 |
| AUD in Treatment [Preg,BC] | 2017 | 1.430 | 1.180 | 0.169 |
| AUD in Treatment [Preg,BC] | 2018 | 0.980 | 1.102 | 0.158 |
| AUD in Treatment [Preg,BC] | 2019 | 0.930 | 1.080 | 0.154 |
| AUD in Treatment [Preg,NBC] | 2016 | 5.930 | 5.487 | 0.845 |
| AUD in Treatment [Preg,NBC] | 2017 | 6.470 | 5.534 | 0.854 |
| AUD in Treatment [Preg,NBC] | 2018 | 5.600 | 5.495 | 0.853 |
| AUD in Treatment [Preg,NBC] | 2019 | 3.940 | 5.245 | 0.810 |

Supplement B3. Table 2. Goodness-of-fit statistics

| Variable Name | MAE of Predicted Mean | RMSE of Predicted Mean | Normalized RMSE of Predicted Mean |
| --- | --- | --- | --- |
| ND Data[Non,BC] | 69.774 | 80.204 | 0.233 |
| ND Data[Non,NBC] | 21.385 | 24.512 | 0.494 |
| ND Data[Preg,BC] | 1.925 | 2.108 | 0.178 |
| ND Data[Preg,NBC] | 11.384 | 14.464 | 0.293 |
| NRD Data[Non,BC] | 47.819 | 58.379 | 0.386 |
| NRD Data[Non,NBC] | 17.008 | 18.208 | 0.248 |
| NRD Data[Preg,BC] | 1.762 | 1.947 | 0.162 |
| NRD Data[Preg,NBC] | 7.134 | 8.561 | 0.137 |
| RD Data[Non,BC] | 25.298 | 28.344 | 0.370 |
| RD Data[Non,NBC] | 7.914 | 8.709 | 0.177 |
| RD Data[Preg,BC] | 1.221 | 1.360 | 0.574 |
| RD Data[Preg,NBC] | 4.261 | 4.669 | 0.426 |
| AUD Data[Non,BC] | 18.850 | 22.349 | 0.221 |
| AUD Data[Non,NBC] | 7.724 | 9.271 | 0.265 |
| AUD Data[Preg,BC] | 0.848 | 0.968 | 0.288 |
| AUD Data[Preg,NBC] | 4.283 | 4.644 | 0.392 |
| Sober NRD Data[Non,BC] | 192.807 | 202.218 | 2.264 |
| Sober NRD Data[Non,NBC] | 1.867 | 2.062 | 0.041 |
| Sober NRD Data[Preg,BC] | 7.747 | 8.696 | 0.293 |
| Sober NRD Data[Preg,NBC] | 38.027 | 48.192 | 0.370 |
| Sober RD Data[Non,BC] | 12.923 | 12.951 | 0.774 |
| Sober RD Data[Non,NBC] | 0.210 | 0.231 | 0.025 |
| Sober RD Data[Preg,BC] | 3.595 | 4.095 | 0.381 |
| Sober RD Data[Preg,NBC] | 13.011 | 16.688 | 0.350 |
| Sober AUD Data[Non,BC] | 23.477 | 25.842 | 4.631 |
| Sober AUD Data[Non,NBC] | 0.126 | 0.174 | 0.056 |
| Sober AUD Data[Preg,BC] | 1.418 | 1.863 | 0.298 |
| Sober AUD Data[Preg,NBC] | 8.776 | 9.039 | 0.289 |
| AUD in Treatment Data[Non,BC] | 5.335 | 6.119 | 0.335 |
| AUD in Treatment Data[Non,NBC] | 2.357 | 2.934 | 0.367 |
| AUD in Treatment Data[Preg,BC] | 0.148 | 0.162 | 0.323 |
| AUD in Treatment Data[Preg,NBC] | 0.697 | 0.835 | 0.330 |

# References

ACOG (2018) Practice Bulletin No. 200: Early Pregnancy Loss. *Obstetrics Gynecology* 132 e197-e207

BANDOLI, G., KABLE, J.A., COLES, C.D., DEL CAMPO, M., SUTTIE, M. AND CHAMBERS, C.D. 2022. Trajectories of prenatal alcohol exposure and behavioral outcomes: Findings from a community-based sample. *Drug Alcohol Depend*, *233*, p.109351.

BOARD, A., D'ANGELO, D. V., VON ESSEN, B. S., DENNY, C. H., MIELE, K., DUNKLEY, J., PARK, Y. & KIM, S. Y. 2023. The Postpartum Period: An Opportunity for Alcohol Screening and Counseling to Reduce Adverse Health Impacts. *J Addict Med,* 17, 528-535.

DAWSON, D. A., GOLDSTEIN, R. B. & GRANT, B. F. 2007. Rates and correlates of relapse among individuals in remission from DSM‐IV alcohol dependence: a 3‐year follow‐up. *Alcohol Clin Exp Res,,* 31, 2036-2045.

DEUTSCH, A.R., CHAU, E., MOTABAR, N. and JALALI, M.S., 2023. Grounding alcohol simulation models in empirical and theoretical alcohol research: a model for a Northern Plains population in the United States. *System Dynamics Rev*, *39*, 207-238.

DOYLE, O., WOOD, E. K., SULLIVAN, E. L., MACKIEWICZ-SEGHETE, K., GRAHAM, A. & GUSTAFSSON, H. C. 2023. COVID-19 pandemic-related trauma symptoms are associated with postpartum alcohol consumption. *Gen Hosp Psychiatry,* 85, 19-27.

FAN, A. Z., CHOU, S. P., ZHANG, H., JUNG, J. & GRANT, B. F. 2019. Prevalence and Correlates of Past-Year Recovery From DSM-5 Alcohol Use Disorder: Results From National Epidemiologic Survey on Alcohol and Related Conditions-III. *Alcohol Clin Exp Res,* 43, 2406-2420.

FORRAY, A., MERRY, B., LIN, H., RUGER, J. P. & YONKERS, K. A. 2015. Perinatal substance use: a prospective evaluation of abstinence and relapse. *Drug Alcohol Depend,* 150, 147-55.

FORTIN, M., MUCKLE, G., ANASSOUR-LAOUAN-SIDI, E., JACOBSON, S. W., JACOBSON, J. L. & BÉLANGER, R. E. 2016. Trajectories of Alcohol Use and Binge Drinking Among Pregnant Inuit Women. *Alcohol Alcohol,* 51, 339-46.

GOURIEROUX, C., MONFORT, A. & TROGNON, A. 1984. Pseudo maximum likelihood methods: Applications to Poisson models. *Econometrica: J Econometric Society***,** 701-720.

HINGSON, R. W., ZHA, W. & WHITE, A. M. 2017. Drinking Beyond the Binge Threshold: Predictors, Consequences, and Changes in the U.S. *Am J Prev Med,* 52, 717-727.

ILGEN, M. A., WILBOURNE, P. L., MOOS, B. S. & MOOS, R. H. 2008. Problem-free drinking over 16 years among individuals with alcohol use disorders. *Drug Alcohol Depend,* 92, 116-22.

JAGODZINSKI, T. & FLEMING, M. F. 2007. Postpartum and alcohol-related factors associated with the relapse of risky drinking. *J Stud Alcohol Drugs,* 68, 879-85.

KLINE‐SIMON, A. H., FALK, D. E., LITTEN, R. Z., MERTENS, J. R., FERTIG, J., RYAN, M. & WEISNER, C. M. 2013. Posttreatment low‐risk drinking as a predictor of future drinking and problem outcomes among individuals with alcohol use disorders. *Alcohol Clin Exp Res,* 37, E373-E380.

KOENIG, L. B., HABER, J. R. & JACOB, T. 2020. Transitions in alcohol use over time: a survival analysis. *BMC Psychology,* 8, 115.

LEE, M. R., BONESS, C. L., MCDOWELL, Y. E., VERGÉS, A., STEINLEY, D. L. & SHER, K. J. 2018. Desistance and Severity of Alcohol Use Disorder: A Lifespan-Developmental Investigation. *Clin Psychol Sci,* 6, 90-105.

LIU, W., MUMFORD, E. A. & PETRAS, H. 2015. Maternal patterns of postpartum alcohol consumption by age: a longitudinal analysis of adult urban mothers. *Prev Sci,* 16, 353-63.

MAISTO, S. A., HALLGREN, K. A., ROOS, C. R. & WITKIEWITZ, K. 2018. Course of remission from and relapse to heavy drinking following outpatient treatment of alcohol use disorder. *Drug Alcohol Depend,* 187, 319-326.

MARTIN-MARTINEZ, E., SAMSO, R., HOUGHTON, J. & SOLE, J. 2022 PySD: System Dynamics Modeling in Python. *J Open Source Software,* 78, 4329

PALZES, V. A., KLINE-SIMON, A. H., SATRE, D. D., STERLING, S., WEISNER, C. & CHI, F. W. 2020. Remission From Unhealthy Drinking Among Patients With an Alcohol Use Disorder: A Longitudinal Study Using Systematic, Primary Care-Based Alcohol Screening Data. *J Stud Alcohol Drugs,* 81, 436-445.

PALZES, V. A., KLINE-SIMON, A. H., SATRE, D. D., STERLING, S., WEISNER, C. & CHI, F. W. 2022. Predictors of early and sustained cessation of heavy drinking over 5 years among adult primary care patients. *Addiction,* 117, 82-95.

QUENBY, S., GALLOS, ID., DHILLON-SMITH, R.K., PODESEK, M., STEPHENSON, M.D., FISHER, J., BROSENS, J.J., BREWIN, J., RAMHORST, R., LUCAS, E.S., MCCOY, R.C., ANDERSON, R., DAHER, S., REGAN, L., AL-MEMAR, M., BOURNE, T., MACINTYRE, D.A., RAI, R., CHRISTIANSEN, O.B., SUGIURA-OGASAWARA, M., ODENDAAL, J., DEVALL, A.J., BENNETT, P.R., PETROU, S.& COOMARASAMY, A., 2021. Miscarriage matters: the epidemiological, physical, psychological, and economic costs of early pregnancy loss. *Lancet,* *397*, 1658-1667

RAHMANDAD, H. & STERMAN, J.D. 2012. Reporting guidelines for simulation-based research in social sciences. *System Dynamics Rev,* 28: 396-411.

SARTOR, C. E., JACKSON, K. M., MCCUTCHEON, V. V., DUNCAN, A. E., GRANT, J. D., WERNER, K. B. & BUCHOLZ, K. K. 2016. Progression from First Drink, First Intoxication, and Regular Drinking to Alcohol Use Disorder: A Comparison of African American and European American Youth. *A lcohol Clin Exp Res,*40, 1515-1523.

SEELEY, J. R., FARMER, R. F., KOSTY, D. B. & GAU, J. M. 2019. Prevalence, incidence, recovery, and recurrence of alcohol use disorders from childhood to age 30. *Drug Alcohol Depend,* 194, 45-50.

SUNDERMANN, A.C., ZHAO, S., YOUNG, C.L., LAM, L., JONES, S.H., VELEZ EDWARDS, D.R., & HARTMANN, K.E., 2019. Alcohol use in pregnancy and miscarriage: a systematic review and meta‐analysis. *Alcohol Clin Exp Res, 43*, 1606-1616.

TUCKER, J. A., CHEONG, J., JAMES, T. G., JUNG, S. & CHANDLER, S. D. 2020. Preresolution Drinking Problem Severity Profiles Associated with Stable Moderation Outcomes of Natural Recovery Attempts. *Alcohol Clin Exp Res,* 44, 738-745.

TUNG, I., CHUNG, T., KRAFTY, R. T., KEENAN, K. & HIPWELL, A. E. 2020. Alcohol Use Trajectories Before and After Pregnancy Among Adolescent and Young Adult Mothers. *Alcohol Clin Exp Res,* 44, 1675-1685.

WHITE, H. 1982. Maximum likelihood estimation of misspecified models. *Econometrica:*

*J Econometric Society***,** 1-25.
