# Supplementary material for: Linking Reductions in Alcohol and Birth Control Use Risk Behavior to Prevention of Alcohol‐Exposed Pregnancy: A Population‐Level Simulation of Preconceptual Prevention Programs
Source: Alcohol Clin Exp Res (Hoboken). 2026 Jul 22;50(7):e70379. doi: 10.1111/acer.70379 (PMC13392302; doi:10.1111/acer.70379)
Supplement: Supplementary file 2 — Data S2: Supporting Information. [file ACER-50-0-s002.docx]

**Supplement E. Supplemental Tables and Figures**

Supplement E Table 1: Percent difference comparing simulation outputs for original AEP-P policy tests (baseline) and alternative sober/non-risky drinking ratio AEP-P policy tests for the number of total non-pregnant drinking women aged 15-44 becoming pregnant (Total AEP Cases) in 2021

|  | | Percent Difference In Alternative Ratio and Original Policy Tests for Number of Total New AEP Cases | | | | | | | | | | | | | | | |  |
| --- | --- | --- | --- | --- | --- | --- | --- | --- | --- | --- | --- | --- | --- | --- | --- | --- | --- | --- |
| Policy Test: Proportion of behavior change groups for AEP-P participants | | 20% Program-Population Impact | |  | 40% Program-Population Impact | | |  | 60% Program-Population Impact | | | |  | 80% Program-Population Impact | | | |  |
| AO/BCO/BB Proportions | | 50/50 Ratio | Reverse Ratio |  | 50/50 Ratio | Reverse Ratio | |  | 50/50 Ratio | | Reverse Ratio | |  | 50/50 Ratio | | Reverse Ratio | |  |
| 100%  Proportions | 100/0/0 | -0.80 | -1.61 |  | -1.51 | | -3.06 | |  | -2.12 | | -4.36 | |  | -2.12 | | -4.36 | |
|  | 0/100/0 | 0.00 | 0.00 |  | 0.00 | | 0.00 | |  | 0.00 | | 0.00 | |  | 0.00 | | 0.00 | |
|  | 0/0/100 | -0.48 | -1.06 |  | -0.53 | | -1.44 | |  | -0.27 | | -1.30 | |  | -0.27 | | -1.30 | |
| 80% / 20% Proportions | 80/20/0 | -0.65 | -1.32 |  | -1.23 | | -2.50 | |  | -1.69 | | -3.46 | |  | -1.69 | | -3.46 | |
|  | 20/80/0 | -0.15 | -0.30 |  | -0.29 | | -0.57 | |  | -0.46 | | -0.93 | |  | -0.46 | | -0.93 | |
|  | 80/0/20 | -0.74 | -1.50 |  | -1.31 | | -2.73 | |  | -1.71 | | -3.67 | |  | -1.71 | | -3.67 | |
|  | 20/0/80 | -0.54 | -1.17 |  | -0.70 | | -1.73 | |  | -0.62 | | -1.86 | |  | -0.62 | | -1.86 | |
|  | 0/80/20 | -0.12 | -0.24 |  | -0.21 | | -0.44 | |  | -0.32 | | -0.69 | |  | -0.32 | | -0.69 | |
|  | 0/20/80 | -0.42 | -0.92 |  | -0.55 | | -1.36 | |  | -0.44 | | -1.38 | |  | -0.44 | | -1.38 | |
| 60% /40% Proportions | 60/40/0 | -0.51 | -1.02 |  | -0.90 | | -1.83 | |  | -1.31 | | -2.66 | |  | -1.31 | | -2.66 | |
|  | 40/60/0 | -0.30 | -0.60 |  | -0.62 | | -1.26 | |  | -0.86 | | -1.74 | |  | -0.86 | | -1.74 | |
|  | 0/60/40 | -0.22 | -0.46 |  | -0.40 | | -0.88 | |  | -0.47 | | -1.07 | |  | -0.47 | | -1.07 | |
|  | 0/40/60 | -0.36 | -0.76 |  | -0.49 | | -1.13 | |  | -0.52 | | -1.34 | |  | -0.52 | | -1.34 | |
|  | 60/0/40 | -0.67 | -1.39 |  | -1.09 | | -2.38 | |  | -1.33 | | -3.05 | |  | -1.33 | | -3.05 | |
|  | 40/0/60 | -0.60 | -1.27 |  | -0.90 | | -2.06 | |  | -0.95 | | -2.42 | |  | -0.95 | | -2.42 | |
| 60% / 20 % / 20%  Proportions | 60/20/20 | -0.60 | -1.22 |  | -1.06 | | -2.21 | |  | -1.35 | | -2.88 | |  | -1.35 | | -2.88 | |
|  | 20/60/20 | -0.26 | -0.53 |  | -0.52 | | -1.07 | |  | -0.65 | | -1.39 | |  | -0.65 | | -1.39 | |
|  | 20/20/60 | -0.48 | -1.01 |  | -0.72 | | -1.64 | |  | -0.72 | | -1.84 | |  | -0.72 | | -1.84 | |
| 40%/ 40% / 20% proportions | 40/20/40 | -0.54 | -1.12 |  | -0.88 | | -1.91 | |  | -1.04 | | -2.36 | |  | -1.04 | | -2.36 | |
|  | 20/40/40 | -0.41 | -0.85 |  | -0.62 | | -1.35 | |  | -0.78 | | -1.77 | |  | -0.78 | | -1.77 | |
|  | 40/40/20 | -0.46 | -0.93 |  | -0.77 | | -1.59 | |  | -1.03 | | -2.19 | |  | -1.03 | | -2.19 | |

Supplement E Table 2: Percent difference comparing simulation outputs for original AEP-P policy tests (baseline) and alternative sober/non-risky drinking ratio AEP-P policy tests for the number of non-pregnant risky drinking and AUD women aged 15-44 becoming pregnant (risky drinking and AUD AEP Cases) in 2021

|  | | Percent Difference In Alternative Ratio and Original Policy Tests for Number of New Risky Drinking and AUD AEP Cases | | | | | | | | | | | | | | | |  |
| --- | --- | --- | --- | --- | --- | --- | --- | --- | --- | --- | --- | --- | --- | --- | --- | --- | --- | --- |
| Policy Test: Proportion of behavior change groups for AEP-P participants | | 20% Program-Population Impact | |  | 40% Program-Population Impact | | |  | 60% Program-Population Impact | | | |  | 80% Program-Population Impact | | | |  |
| AO/BCO/BB Proportions | | 50/50 Ratio | Reverse Ratio |  | 50/50 Ratio | Reverse Ratio | |  | 50/50 Ratio | | Reverse Ratio | |  | 50/50 Ratio | | Reverse Ratio | |  |
| 100%  Proportions | 100/0/0 | 0.18 | 0.35 |  | 0.34 | | 0.68 | |  | 0.50 | | 1.00 | |  | 0.64 | | 1.28 | |
|  | 0/100/0 | 0.00 | 0.00 |  | 0.00 | | 0.00 | |  | 0.00 | | 0.00 | |  | 0.00 | | 0.00 | |
|  | 0/0/100 | 0.17 | 0.31 |  | 0.30 | | 0.48 | |  | 0.40 | | 0.53 | |  | 0.46 | | 0.51 | |
| 80% / 20% Proportions | 80/20/0 | 0.14 | 0.29 |  | 0.28 | | 0.56 | |  | 0.39 | | 0.78 | |  | 0.51 | | 1.02 | |
|  | 20/80/0 | 0.03 | 0.06 |  | 0.06 | | 0.13 | |  | 0.10 | | 0.21 | |  | 0.13 | | 0.26 | |
|  | 80/0/20 | 0.17 | 0.34 |  | 0.33 | | 0.64 | |  | 0.48 | | 0.89 | |  | 0.60 | | 1.10 | |
|  | 20/0/80 | 0.17 | 0.31 |  | 0.31 | | 0.51 | |  | 0.42 | | 0.62 | |  | 0.49 | | 0.64 | |
|  | 0/80/20 | 0.03 | 0.07 |  | 0.06 | | 0.12 | |  | 0.10 | | 0.20 | |  | 0.13 | | 0.23 | |
|  | 0/20/80 | 0.14 | 0.26 |  | 0.26 | | 0.43 | |  | 0.33 | | 0.49 | |  | 0.39 | | 0.51 | |
| 60% /40% Proportions | 60/40/0 | 0.11 | 0.22 |  | 0.20 | | 0.40 | |  | 0.30 | | 0.60 | |  | 0.38 | | 0.75 | |
|  | 40/60/0 | 0.06 | 0.13 |  | 0.14 | | 0.28 | |  | 0.20 | | 0.39 | |  | 0.26 | | 0.52 | |
|  | 0/60/40 | 0.07 | 0.13 |  | 0.14 | | 0.25 | |  | 0.18 | | 0.32 | |  | 0.24 | | 0.39 | |
|  | 0/40/60 | 0.11 | 0.21 |  | 0.19 | | 0.34 | |  | 0.27 | | 0.44 | |  | 0.31 | | 0.47 | |
|  | 60/0/40 | 0.17 | 0.33 |  | 0.33 | | 0.60 | |  | 0.46 | | 0.80 | |  | 0.56 | | 0.93 | |
|  | 40/0/60 | 0.17 | 0.32 |  | 0.32 | | 0.56 | |  | 0.44 | | 0.70 | |  | 0.53 | | 0.78 | |
| 60% / 20 % / 20%  Proportions | 60/20/20 | 0.14 | 0.28 |  | 0.27 | | 0.53 | |  | 0.38 | | 0.70 | |  | 0.48 | | 0.87 | |
|  | 20/60/20 | 0.07 | 0.13 |  | 0.14 | | 0.27 | |  | 0.19 | | 0.36 | |  | 0.25 | | 0.45 | |
|  | 20/20/60 | 0.14 | 0.27 |  | 0.26 | | 0.46 | |  | 0.35 | | 0.56 | |  | 0.42 | | 0.63 | |
| 40%/ 40% / 20% proportions | 40/20/40 | 0.14 | 0.27 |  | 0.27 | | 0.49 | |  | 0.36 | | 0.63 | |  | 0.45 | | 0.74 | |
|  | 20/40/40 | 0.11 | 0.21 |  | 0.20 | | 0.36 | |  | 0.28 | | 0.49 | |  | 0.34 | | 0.56 | |
|  | 40/40/20 | 0.11 | 0.22 |  | 0.20 | | 0.38 | |  | 0.29 | | 0.54 | |  | 0.36 | | 0.65 | |

Supplement E Table 3: Percent difference comparing simulation outputs for original AEP-P policy tests (baseline) and alternative sober/non-risky drinking ratio AEP-P policy tests for the number of total non-pregnant non-risky drinking women aged 15-44 becoming pregnant (non-risky AEP Cases) in 2021

|  | | Percent Difference In Alternative Ratio and Original Policy Tests for Number of New Non-Risky Drinking AEP Cases | | | | | | | | | | | | | | | |  |
| --- | --- | --- | --- | --- | --- | --- | --- | --- | --- | --- | --- | --- | --- | --- | --- | --- | --- | --- |
| Policy Test: Proportion of behavior change groups for AEP-P participants | | 20% Program-Population Impact | |  | 40% Program-Population Impact | | |  | 60% Program-Population Impact | | | |  | 80% Program-Population Impact | | | |  |
| AO/BCO/BB Proportions | | 50/50 Ratio | Reverse Ratio |  | 50/50 Ratio | Reverse Ratio | |  | 50/50 Ratio | | Reverse Ratio | |  | 50/50 Ratio | | Reverse Ratio | |  |
| 100%  Proportions | 100/0/0 | -1.46 | -2.96 |  | -2.62 | | -5.40 | |  | -3.56 | | -7.41 | |  | -4.32 | | -9.07 | |
|  | 0/100/0 | 0.00 | 0.00 |  | 0.00 | | 0.00 | |  | 0.00 | | 0.00 | |  | 0.00 | | 0.00 | |
|  | 0/0/100 | -0.88 | -1.94 |  | -0.98 | | -2.49 | |  | -0.59 | | -2.19 | |  | 0.09 | | -1.35 | |
| 80% / 20% Proportions | 80/20/0 | -1.20 | -2.42 |  | -2.16 | | -4.43 | |  | -2.86 | | -5.90 | |  | -3.51 | | -7.30 | |
|  | 20/80/0 | -0.28 | -0.56 |  | -0.51 | | -1.03 | |  | -0.80 | | -1.61 | |  | -0.96 | | -1.93 | |
|  | 80/0/20 | -1.34 | -2.75 |  | -2.27 | | -4.77 | |  | -2.86 | | -6.18 | |  | -3.27 | | -7.20 | |
|  | 20/0/80 | -0.99 | -2.12 |  | -1.26 | | -3.00 | |  | -1.12 | | -3.10 | |  | -0.65 | | -2.62 | |
|  | 0/80/20 | -0.23 | -0.46 |  | -0.39 | | -0.80 | |  | -0.57 | | -1.20 | |  | -0.63 | | -1.35 | |
|  | 0/20/80 | -0.79 | -1.69 |  | -1.00 | | -2.38 | |  | -0.83 | | -2.32 | |  | -0.45 | | -1.94 | |
| 60% /40% Proportions | 60/40/0 | -0.93 | -1.88 |  | -1.59 | | -3.24 | |  | -2.22 | | -4.56 | |  | -2.67 | | -5.49 | |
|  | 40/60/0 | -0.55 | -1.11 |  | -1.11 | | -2.24 | |  | -1.48 | | -3.00 | |  | -1.87 | | -3.82 | |
|  | 0/60/40 | -0.42 | -0.86 |  | -0.73 | | -1.56 | |  | -0.83 | | -1.85 | |  | -0.87 | | -2.06 | |
|  | 0/40/60 | -0.66 | -1.40 |  | -0.89 | | -1.98 | |  | -0.93 | | -2.28 | |  | -0.77 | | -2.17 | |
|  | 60/0/40 | -1.23 | -2.55 |  | -1.91 | | -4.13 | |  | -2.26 | | -5.09 | |  | -2.31 | | -5.50 | |
|  | 40/0/60 | -1.10 | -2.32 |  | -1.59 | | -3.57 | |  | -1.65 | | -4.03 | |  | -1.47 | | -4.02 | |
| 60% / 20 % / 20%  Proportions | 60/20/20 | -1.09 | -2.24 |  | -1.86 | | -3.89 | |  | -2.28 | | -4.88 | |  | -2.63 | | -5.74 | |
|  | 20/60/20 | -0.48 | -0.98 |  | -0.92 | | -1.90 | |  | -1.13 | | -2.39 | |  | -1.35 | | -2.90 | |
|  | 20/20/60 | -0.88 | -1.86 |  | -1.28 | | -2.87 | |  | -1.27 | | -3.09 | |  | -1.13 | | -3.10 | |
| 40%/ 40% / 20% proportions | 40/20/40 | -0.99 | -2.06 |  | -1.55 | | -3.35 | |  | -1.77 | | -3.97 | |  | -1.83 | | -4.33 | |
|  | 20/40/40 | -0.76 | -1.57 |  | -1.10 | | -2.37 | |  | -1.34 | | -2.99 | |  | -1.33 | | -3.14 | |
|  | 40/40/20 | -0.84 | -1.72 |  | -1.35 | | -2.81 | |  | -1.75 | | -3.73 | |  | -1.97 | | -4.26 | |

Supplement E Figure 1 Percent difference comparing simulation outputs for baseline status-quo and each AEP-P policy test for the number of total drinkers becoming pregnant (Total AEP Cases) in 2021 (e.g., percent decrease or increase in the number of all drinkers getting pregnant for each AEP-P policy implemented compared to baseline).

Supplement E Figure 2 Percent difference comparing simulation outputs for baseline status-quo and each AEP-P policy test for the number of risky drinking and women with AUD becoming pregnant (Risky/AUD AEP Cases) in 2021 (e.g., percent decrease or increase in the number of risky drinking and AUD people getting pregnant for each AEP-P policy implemented compared to baseline).

Supplement E Figure 3 Percent difference comparing simulation outputs for baseline status-quo and each AEP-P policy test for the number of non-risky drinkers becoming pregnant (Non-Risky AEP Cases) in 2021 (e.g., percent decrease or increase in the number of non-risky drinking people getting pregnant for each AEP-P policy implemented compared to baseline)

Supplement E Figure 4a. Percent difference in simulation outputs for next-year number of all non-pregnant women drinkers aged 15-44 becoming pregnant in 2021, comparing baseline non-intervention (status-quo) baseline simulation and both original and alternative policy test simulations, at 20% program population impact.

Supplement E Figure 4b. Percent difference in simulation outputs for next-year number of all non-pregnant women drinkers aged 15-44 becoming pregnant in 2021, comparing baseline non-intervention (status-quo) baseline simulation and both original and alternative policy test simulations, at 40% program population impact.

Supplement E Figure 4c. Percent difference in simulation outputs for next-year number of all non-pregnant women drinkers aged 15-44 becoming pregnant in 2021, comparing baseline non-intervention (status-quo) baseline simulation and both original and alternative policy test simulations, at 60% program population impact.

Supplement E Figure 4d. Percent difference in simulation outputs for next-year number of all non-pregnant women drinkers aged 15-44 becoming pregnant in 2021, comparing baseline non-intervention (status-quo) baseline simulation and both original and alternative policy test simulations, at 80% program population impact.

Supplement E Figure 5a. Percent difference in simulation outputs for next-year number of all non-pregnant risky drinking and AUD women aged 15-44 becoming pregnant in 2021, comparing baseline non-intervention (status-quo) baseline simulation and both original and alternative policy test simulations, at 20% program population impact.

Supplement E Figure 5b. Percent difference in simulation outputs for next-year number of all non-pregnant risky drinking and AUD women aged 15-44 becoming pregnant in 2021, comparing baseline non-intervention (status-quo) baseline simulation and both original and alternative policy test simulations, at 40% program population impact.

Supplement E Figure 5c. Percent difference in simulation outputs for next-year number of all non-pregnant risky drinking and AUD women aged 15-44 becoming pregnant in 2021, comparing baseline non-intervention (status-quo) baseline simulation and both original and alternative policy test simulations, at 60% program population impact.

Supplement E Figure 5d. Percent difference in simulation outputs for next-year number of all non-pregnant risky drinking and AUD women aged 15-44 becoming pregnant in 2021, comparing baseline non-intervention (status-quo) baseline simulation and both original and alternative policy test simulations, at 80% program population impact.

Supplement E Figure 6a. Percent difference in simulation outputs for next-year number of all non-pregnant non-risky drinking women aged 15-44 becoming pregnant in 2021, comparing baseline non-intervention (status-quo) baseline simulation and both original and alternative policy test simulations, at 20% program population impact.

Supplement E Figure 6b. Percent difference in simulation outputs for next-year number of all non-pregnant non-risky drinking women aged 15-44 becoming pregnant in 2021, comparing baseline non-intervention (status-quo) baseline simulation and both original and alternative policy test simulations, at 40% program population impact.

Supplement E Figure 6c. Percent difference in simulation outputs for next-year number of all non-pregnant non-risky drinking women aged 15-44 becoming pregnant in 2021, comparing baseline non-intervention (status-quo) baseline simulation and both original and alternative policy test simulations, at 60% program population impact.

Supplement E Figure 6d. Percent difference in simulation outputs for next-year number of all non-pregnant non-risky drinking women aged 15-44 becoming pregnant in 2021, comparing baseline non-intervention (status-quo) baseline simulation and both original and alternative policy test simulations, at 80% program population impact.
